# Lyrebird Optimization Algorithm: A New Bio-Inspired Metaheuristic Algorithm for Solving Optimization Problems

**DOI:** 10.3390/biomimetics8060507

**Published:** 2023-10-23

**Authors:** Mohammad Dehghani, Gulnara Bektemyssova, Zeinab Montazeri, Galymzhan Shaikemelev, Om Parkash Malik, Gaurav Dhiman

**Affiliations:** 1 Department of Electrical and Electronics Engineering, Shiraz University of Technology, Shiraz 7155713876, Iran; z.montazeri@sutech.ac.ir; 2 Department of Computer Engineering, International Information Technology University, Almaty 050000, Kazakhstan; 33271@iitu.edu.kz; 3 Department of Electrical and Software Engineering, University of Calgary, Calgary, AB T2N 1N4, Canada; maliko@ucalgary.ca; 4 Department of Electrical and Computer Engineering, Lebanese American University, Byblos 13-5053, Lebanon; gdhiman0001@gmail.com; 5 Department of Computer Science and Engineering, University Centre for Research and Development, Chandigarh University, Mohali 140413, India; 6 Department of Computer Science and Engineering, Graphic Era Deemed to be University, Dehradun 248002, India; 7 Division of Research and Development, Lovely Professional University, Phagwara 144411, India

**Keywords:** optimization, bio-inspired, metaheuristic, lyrebird, exploration, exploitation

## Abstract

In this paper, a new bio-inspired metaheuristic algorithm called the Lyrebird Optimization Algorithm (LOA) that imitates the natural behavior of lyrebirds in the wild is introduced. The fundamental inspiration of LOA is the strategy of lyrebirds when faced with danger. In this situation, lyrebirds scan their surroundings carefully, then either run away or hide somewhere, immobile. LOA theory is described and then mathematically modeled in two phases: (i) exploration based on simulation of the lyrebird escape strategy and (ii) exploitation based on simulation of the hiding strategy. The performance of LOA was evaluated in optimization of the CEC 2017 test suite for problem dimensions equal to 10, 30, 50, and 100. The optimization results show that the proposed LOA approach has high ability in terms of exploration, exploitation, and balancing them during the search process in the problem-solving space. In order to evaluate the capability of LOA in dealing with optimization tasks, the results obtained from the proposed approach were compared with the performance of twelve well-known metaheuristic algorithms. The simulation results show that LOA has superior performance compared to competitor algorithms by providing better results in the optimization of most of the benchmark functions, achieving the rank of first best optimizer. A statistical analysis of the performance of the metaheuristic algorithms shows that LOA has significant statistical superiority in comparison with the compared algorithms. In addition, the efficiency of LOA in handling real-world applications was investigated through dealing with twenty-two constrained optimization problems from the CEC 2011 test suite and four engineering design problems. The simulation results show that LOA has effective performance in handling optimization tasks in real-world applications while providing better results compared to competitor algorithms.

## 1. Introduction

In mathematics, a problem that has more than one feasible solution is known as an optimization problem. According to this definition, the process of obtaining the best solution among all available solutions for an optimization problem is called optimization [1]. Every optimization problem can be mathematically modeled using the three components of decision variables, constraints, and the objective function. The main goal in optimization is to value the decision variables so that the objective function is optimized by respecting the constraints of the problem [2]. There are numerous optimization problems in mathematics, science, industry, engineering, and real-world applications that need to be optimized using appropriate methods. The problem-solving methods in dealing with optimization problems are classified into two groups: deterministic and stochastic approaches [3].

Deterministic approaches have good performance in dealing with linear, differentiable, continuous, low-dimensional, and simple optimization problems [4]. However, with greater complexity of optimization problems and increases in problem dimensions, deterministic approaches lose their efficiency by becoming stuck in local optima [5]. This is despite the fact that many optimization problems in mathematics, engineering, industry, technology, and other branches of science have complex, nonlinear, non-continuous, non-differentiable, high-dimensional natures. The difficulties and disadvantages of deterministic approaches led researchers to develop stochastic approaches in order to deal with practical optimization problems [6].

Metaheuristic algorithms are one of the most effective and widely used stochastic approaches that are able to provide suitable solutions for optimization problems based on random search in the problem-solving space and by using random operators and trial and error processes. The optimization mechanism in metaheuristic algorithms is such that first a number of candidate solutions are randomly generated. Then, during algorithm iterations, these initial solutions are improved based on algorithm update steps. In each iteration, the best candidate solution is updated and saved. After the full implementation of the algorithm, the best candidate solution is available as a solution to the problem [7]. The important point about the solutions obtained from metaheuristic algorithms is that due to the nature of random search in these methods, there is no guarantee that the global optimal solution will be found. However, due to the proximity of the solutions obtained from metaheuristic algorithms to the global optimum, these solutions are called quasi-optimal. Achieving more effective quasi-optimal solutions for optimization problems has been the main motivation of researchers in the development of numerous metaheuristic algorithms [8]. These metaheuristic algorithms have been used to solve optimization problems in sciences such as Internet of Things (IoT) applications [9,10,11,12,13], data mining [14], wireless network systems [15], clustering [16,17], power engineering applications [18,19,20], and feature selection [21,22].

A metaheuristic algorithm can provide effective solutions for optimization problems when it is able to accurately search the problem-solving space at both global and local levels. Global search with the concept of exploration refers to the performance of the metaheuristic algorithm in the comprehensive scanning of the problem-solving space with the aim of discovering the region containing the original optimal solution and preventing the algorithm from becoming stuck in inappropriate local optima. Local search with the concept of exploitation refers to the performance of the metaheuristic algorithm in accurate scanning with small steps around the promising areas of the problem-solving space and the proximity of the discovered solutions. Considering that exploration and exploitation pursue different goals, the key to the success of the metaheuristic algorithm in optimization is balancing them during the search process [23].

The main research question is: According to the numerous metaheuristic algorithms designed so far, is there still a need to introduce new metaheuristic algorithms? In response to this question, the No Free Lunch (NFL) [24] theorem explains that the successful performance of a metaheuristic algorithm in handling a group of optimization problems is not a guarantee for the similar performance of that algorithm in handling other optimization problems. In fact, there is always a possibility that an algorithm may reach the global optimum in solving an optimization problem, but it will fail in solving another problem by becoming stuck in the local optimum. Therefore, there is no presumption of the success or failure of implementing an algorithm on an optimization problem. Based on the NFL theorem, it cannot be claimed that a unique metaheuristic algorithm is the best optimizer to solve all optimization problems. By keeping the studies of metaheuristic algorithms active, the NFL theorem motivates researchers to provide more effective solutions for optimization problems by designing newer algorithms. The authors of this paper are also motivated by the NFL theorem to design a new metaheuristic algorithm to solve optimization problems.

The innovation and novelty of this article is in the introduction of a new bio-inspired metaheuristic algorithm called the Lyrebird Optimization Algorithm (LOA) that has applications in dealing with optimization problems. The contributions of this paper are as follows:

- LOA is introduced as mimicking the natural behavior of lyrebirds in the wild.
- The fundamental inspiration of LOA is derived from the strategies of lyrebirds when they sense danger.
- LOA theory is expressed and mathematically modeled in two phases of (i) exploration based on simulation of the escape strategy and (ii) exploitation based on simulation of the hiding strategy.
- The performance of LOA was evaluated using the CEC 2017 test suite for problem dimensions of 10, 30, 50, and 100.
- The performance of LOA in handling real-world applications was evaluated in handling twenty-two constrained optimization problems from the CEC 2011 test suite and four engineering design problems.
- The results obtained from LOA were compared with the performance of twelve well-known metaheuristic algorithms.

The structure of the paper is as follows: A review of the literature is presented in Section 2. Then, the proposed Lyrebird Optimization Algorithm is introduced and modeled in Section 3. The simulation studies and results are presented in Section 4. The effectiveness of LOA in solving real-world applications is investigated in Section 5. Conclusions and suggestions for future research are provided in Section 6.

## 2. Literature Review

Metaheuristic algorithms are designed with inspiration from various natural phenomena, natural behaviors of living organisms in nature, biological sciences, physical concepts, human lifestyles, rules of games, and other evolutionary processes. Metaheuristic algorithms with respect to the main source of inspiration in the design are classified into five groups: swarm-based, evolutionary-based, physics-based, human-based, and game-based approaches.

Swarm-based metaheuristic algorithms are inspired in their design by the natural behavior of birds, insects, aquatic animals, reptiles, and other living organisms in nature. Among the most famous swarm-based metaheuristic algorithms are Particle Swarm Optimization (PSO) [25], Ant Colony Optimization (ACO) [26], Artificial Bee Colony (ABC) [27], and Firefly Algorithm (FA) [28]. PSO is inspired by the exploratory movement of birds and fish looking for food. ACO was introduced based on modeling the ability of ants to find the shortest communication path between the colony and the food location. ABC was designed based on simulating the activities of honey bees in a colony searching for food sources. FA was proposed inspired by optical communication between fireflies. The Grey Wolf Optimizer (GWO) was designed based on modeling the hierarchical leadership of gray wolves during hunting [29]. The strategy of pelicans while hunting was a source of inspiration in the design of the Pelican Optimization Algorithm (POA) [30]. The coatis’ strategy when hunting iguanas and their behavior when escaping from predators were employed in the design of Coati Optimization Algorithm (COA) [31]. Hunting strategy, foraging, chasing, migration, and digging are among the most characteristic natural behaviors in wildlife and have been the main source of inspiration in the design of swarm-based metaheuristic algorithms such as the Gazelle Optimization Algorithm (GOA) [32], Marine Predator Algorithm (MPA) [33], Nutcracker Optimization (NO) [34], Reptile Search Algorithm (RSA) [35], Sea-Horse Optimizer (SHO) [36], White Shark Optimizer (WSO) [37], Golden Jackal Optimization (GJO) [38], African Vultures Optimization Algorithm (AVOA) [39], Orca Predation Algorithm (OPA) [40], Tunicate Swarm Algorithm (TSA) [41], Whale Optimization Algorithm (WOA) [42], and Honey Badger Algorithm (HBA) [43].

Evolutionarily based metaheuristic algorithms are inspired in their design by biological sciences, genetics, concepts of natural selection, survival of the fittest, and evolutionary stochastic operators. The Genetic Algorithm (GA) [44] and Differential Evolution (DE) [45] are the most well-known algorithms of this group and were designed based on simulation of the reproduction process and the application of genetic concepts, Darwin’s theory of evolution, natural selection, and the random operators of mutation, crossover, and selection. The mechanism of the human body’s defense system against diseases and microbes was the source of inspiration in the design of Artificial Immune Systems (AISs) [46]. Some other evolutionarily based metaheuristic algorithms are the Cultural Algorithm (CA) [47], Genetic Programming (GP) [48], and Evolution Strategy (ES) [49].

Physics-based metaheuristic algorithms are inspired in their design by forces, laws of motion, transformations, and various concepts in physics. Simulated Annealing (SA) is one of the most widely used physics-based algorithms whose main design idea is derived from the metal annealing process in which metals are first melted under heat and then slowly cooled with the aim of achieving an ideal crystal [50]. Physical forces and Newton’s laws of motion were employed in designing algorithms such as the Momentum Search Algorithm (MSA) [51] inspired by momentum force, Gravitational Search Algorithm (GSA) inspired by gravitational attraction force [52], and Spring Search Algorithm (SSA) [53] inspired by the elastic force of a spring and Hooke’s law. Concepts of cosmology have been the source of inspiration in the design of the Multi-Verse Optimizer (MVO) [54] and Black Hole Algorithm (BHA) [55]. Some other physics-based metaheuristic algorithms are Nuclear Reaction Optimization (NRO) [56], Electro-Magnetism Optimization (EMO) [57], the Water Cycle Algorithm (WCA) [58], Equilibrium Optimizer (EO) [59], Thermal Exchange Optimization (TEO) [60], Henry Gas Optimization (HGO) [61], Archimedes Optimization Algorithm (AOA) [62], and Lichtenberg Algorithm (LA) [63].

Human-based metaheuristic algorithms are inspired in their design by human thoughts, choices, decisions, communication, interactions, and other activities. Teaching–Learning Based Optimization (TLBO) is one of the most well-known human-based algorithms whose design is based on the classroom learning environment and interactions between teachers and students and students with each other [64]. The Mother Optimization Algorithm (MOA) was introduced based on the modeling of Eshrat’s care of her children [65]. The holding of elections and the voting process were employed in the design of Election-Based Optimization Algorithm (EBOA) [66]. The process of driving education in school and interactions between instructors and applicants was the main idea in the design of Driving-Training-Based Optimization (DTBO) [67]. Collaboration between team members to address assigned tasks was the source of inspiration in the design of the Teamwork Optimization Algorithm (TOA) [68]. Some other human-based metaheuristic algorithms are the Gaining Sharing Knowledge-based Algorithm (GSK) [69], Skill Optimization Algorithm (SOA) [70], War Strategy Optimization (WSO) [71], Deep Sleep Optimizer (DSO) [72], Ali Baba and the Forty Thieves (AFT) [73], and Coronavirus Herd Immunity Optimizer (CHIO) [74].

Game-based metaheuristic algorithms are inspired in their design by the rules and behavior of players, coaches, referees and other effective people in various individual and team games. The Darts Game Optimizer (DGO) is one of the most famous game-based algorithms, designed with the inspiration of modeling the skill of players in throwing darts and collecting points in a game of darts [75]. Holding club matches in a league was employed in designing algorithms such as Football-Game-Based Optimization (FGBO) [76] and the Volleyball Premier League (VPL) [77]. The skill of the players in putting together the pieces of a puzzle was the main idea in the design of the Puzzle Optimization Algorithm (POA) [78]. Some other game-based metaheuristic algorithms are the Running City Game Optimizer (RCGO) [79], Tug of War Optimization (TWO) [80], Billiards Optimization Algorithm(BOA) [81], and Golf Optimization Algorithm (GOA) [82].

In addition to the mentioned classifications of metaheuristic algorithms, hybrid approaches have been developed based on the combination of two or more metaheuristic algorithms. The main motivation in hybrid approaches is to benefit from the advantages of several algorithms in the form of an integrated algorithm [83]. Among the hybrid approach, of note are hybrid PSO-GA [84], hybrid GA-PSO-TLBO [85], hybrid GWO-WOA [86], and hybrid TSA-PSO [87].

Although a hybrid approach is expected to perform better than the individual versions of its constituent algorithms, based on the NFL theorem, there is no guarantee for this issue. Also, hybrid approaches were developed in order to improve the performance of existing metaheuristic algorithms, but there is always a possibility that based on a different perspective on the types of emerging optimization problems in science and real-world applications, newer algorithms will be designed that have better performance than existing algorithms.

Based on the best knowledge obtained from our literature review, so far, no metaheuristic algorithm has been designed inspired by the natural behavior of lyrebirds in the wild. Meanwhile, the strategy of this bird when it feels danger is an intelligent process that can be the basis for designing an optimizer. Lyrebirds, when faced with danger, decide to run away or hide somewhere based on scanning their surroundings. In order to address this research gap in the studies of meta-heuristic algorithms, in this paper, a new bio-inspired metaheuristic algorithm based on the modeling of lyrebird strategy during their sensing of danger is introduced, which is discussed in the next section.

As explained in the introduction section, in order to manage an effective search process in the problem-solving space, a metaheuristic algorithm must have high ability in exploration, exploitation, and balancing them during the search process. In the design of LOA, taking into account the separate phases of updating based on the powers of exploration and exploitation, as well as how to manage them during iterations of the algorithm, an effort was made to achieve an effective and powerful search process in the problem-solving space in order to achieve suitable solutions for optimization problems.

In the design of the LOA, the exploration capability to manage the global search is modeled based on the simulation of the lyrebird’s escape strategy when faced with danger. In this strategy, the lyrebird flees to a random location in the wild. The modeling of this strategy leads to a global search in order to comprehensively scan the problem-solving space with the aim of preventing becoming stuck in local optima and discovering the main region including the global optima. According to this, LOA is expected to be effective in the exploration for global search in the problem-solving space.

In the LOA design, the exploitation ability to manage local search is modeled based on the simulation of the lyrebird’s hiding strategy when faced with danger. In this strategy, the lyrebird moves to a safe position to hide from the enemy by scanning its surroundings. The modeling of this strategy represents a local search with the aim of achieving better possible solutions for the given problem. Therefore, LOA is expected to perform well in exploitation for local search in the problem-solving space with the aim of achieving better solutions.

On the other hand, in order to balance exploration and exploitation in LOA, priority was given to exploration in the initial iterations so that by making extensive changes in the position of population members, the problem-solving space can be comprehensively scanned and promising areas can be discovered. Then, by increasing the iterations of the algorithm, by reducing the local search scope, priority is given to exploitation, so that based on a detailed scan around the obtained solutions and promising areas, the algorithm converges towards solutions close to the global optimum. Therefore, it is expected that the proposed LOA approach has good performance in exploration, exploitation, and balancing them during the search process in the problem-solving space so that it can achieve suitable solutions for optimization problems by managing an effective search process.

## 3. Lyrebird Optimization Algorithm

In this section, the inspiration of the proposed Lyrebird Optimization Algorithm (LOA) is expressed, then its mathematical modeling is presented in order to be used in optimization applications.

### 3.1. Inspiration of LOA

**Figure 1:**
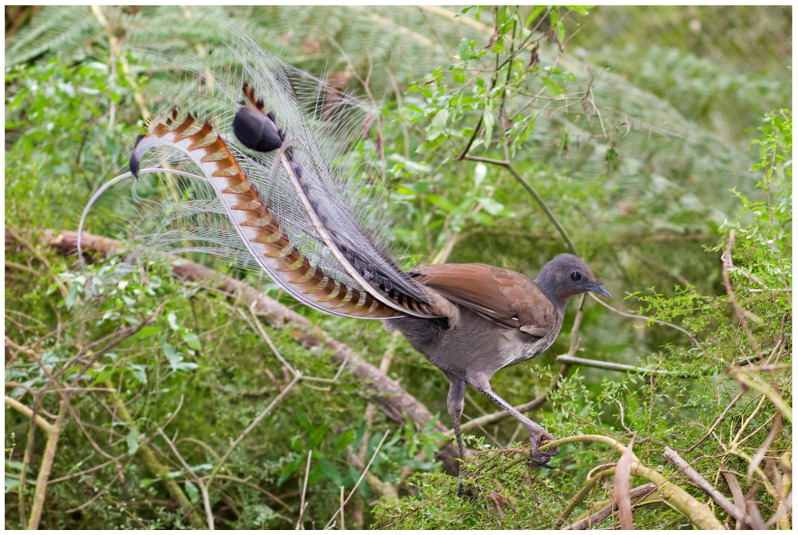
Lyrebird taken from: free media Wikimedia Commons.

The lyrebird is a native Australian bird composed of two species, the Superb lyrebird and Albert’s lyrebird. This amazing bird is a member of the Menuridae family [88]. They are mostly noted for the striking beauty of the male bird’s huge tail when it is fanned out in a courtship display and their excellent ability to imitate artificial and natural sounds from their environment [89]. Lyrebirds have unique plumes of neutral-colored tailfeathers and are among Australia’s best-known native birds. The Superb lyrebird species has a length of 74–84 cm in females and 80–98 cm in males. Meanwhile, Albert’s lyrebird is a little smaller, such that the maximum size of the female is 84 cm and that of the male is 90 cm. Albert’s lyrebird species has smaller, less spectacular lyrate feathers than the Superb lyrebird, but are otherwise similar. They weigh about 0.93 kg, while Superb lyrebirds are slightly heavier at about 0.97 kg. An image of a lyrebird is shown in Figure 1.

One of the behavioral characteristics of the lyrebird is apparent when it senses potential danger. In this situation, this bird pauses, first scans its surroundings carefully, then either escapes from that environment or hides in a suitable place. Mathematical modeling of this strategy of lyrebirds during danger was employed in the design of the proposed LOA approach, which is discussed below.

### 3.2. Algorithm Initialization

The proposed LOA approach is a population-based metaheuristic algorithm where lyrebirds constitute the population. The LOA is able to provide suitable solutions for optimization problems in an iteration-based process by using the searching power of its members in the problem-solving space. Each lyrebird, as a LOA member, determines the values of the decision variables based on its position in the problem-solving space. Therefore, from a mathematical point of view, each lyrebird can be modeled using a vector so that each element of this vector represents a decision variable. LOA members together form the population of the algorithm, which can be mathematically modeled using a matrix according to Equation (1). The initial position of LOA members in the problem-solving space is initialized randomly using Equation (2).

(1) $$X={\left[\begin{array}{c} X_{1}\\ \vdots \\ X_{i}\\ \vdots \\ X_{N} \end{array}\right]}_{N\times m}={\left[\begin{array}{ccccc} x_{1,1} & \cdots & x_{1,d} & \cdots & x_{1,m}\\ \vdots & \ddots & \vdots & ⋰ & \vdots \\ x_{i,1} & \cdots & x_{i,d} & \cdots & x_{i,m}\\ \vdots & ⋰ & \vdots & \ddots & \vdots \\ x_{N,1} & \cdots & x_{N,d} & \cdots & x_{N,m} \end{array}\right]}_{N\times m}$$

(2) $$x_{i,d}=lb_{d}+r\cdot (ub_{d}-lb_{d})$$

Here, X is the LOA population matrix, $X_{i}$ is the ith LOA member (candidate solution), $x_{i,d}$ is its dth dimension in search space (decision variable), N is the number of lyrebirds, m is the number of decision variables, r is a random number in interval $\left[0,1\right]$, $lb_{d}$, and $ub_{d}$ are the lower bound and upper bound of the dth decision variable, respectively.

Considering that each LOA member is a candidate solution to the problem, corresponding to each LOA member, the objective function of the problem can be evaluated. Therefore, equal to the number of population members, the values for the objective function are available. The set of evaluated values for the objective function of the problem can be represented using a vector according to Equation (3).

(3) $$F={\left[\begin{array}{c} F_{1}\\ \vdots \\ F_{i}\\ \vdots \\ F_{N} \end{array}\right]}_{N\times 1}={\left[\begin{array}{c} F(X_{1})\\ \vdots \\ F(X_{i})\\ \vdots \\ F(X_{N}) \end{array}\right]}_{N\times 1}$$

Here, F is the vector of evaluated objective function and $F_{i}$ is the evaluated objective function based on the ith LOA member.

The evaluated values for the objective function are a suitable criterion for measuring the quality of the candidate solutions. According to this, the best evaluated value for the objective function corresponds to the best candidate solution (i.e., the best LOA member), and the worst evaluated value for the objective function corresponds to the worst candidate solution (i.e., the worst LOA member). Also, considering that in each iteration, the position of lyrebirds in the problem-solving space is updated, the best candidate solution should also be updated based on the comparison of the objective function value.

### 3.3. Mathematical Modelling of LOA

In the design of the proposed LOA approach, the position of the population members is updated in each iteration based on the mathematical modeling of the lyrebird strategy when sensing danger. Based on the decision of lyrebird in this situation, the population update process has two phases of (i) escaping and (ii) hiding.

In the design of LOA, the lyrebird’s decision-making process in order to choose one of the escape or hiding strategies during danger is simulated using Equation (4). Therefore, in each iteration, the position of each LOA member is updated only based on one of the first or second phases.

(4) $$\mathrm{Update}\;\mathrm{process}\;\mathrm{for}\;X_{i}:\left\{\begin{array}{l} based\;on\;Phase\;1,\;\;r_{p}\leq 0.5\\ based\;on\;Phase\;2,\;\;else\; \end{array}\right.$$

Here, $r_{p}$ is a random number from the interval $\left[0,\;1\right]$.

#### 3.3.1. Phase 1: Escaping Strategy (Exploration Phase)

In this phase of LOA, the position of the population member is updated in the search space based on the modeling of the lyrebird’s escape from the danger position to the safe areas. Moving the lyrebird to the safe area leads to extensive changes in its position and scanning different areas in the problem-solving space, which indicates the exploration ability of LOA in global search. In LOA design, for each member, the position of other population members who have better objective function value are considered as safe areas. Therefore, the set of safe areas for each LOA member can be determined using Equation (5).

(5) $${SA}_{i}=\left\{X_{k},\;\;\;\;\;\;\;F_{k}<F_{i}\mathrm{and}k\in \left\{1,2,..,N\right\}\right\},\;\;\;\;\;\;\;\mathrm{where}i=1,2,\ldots ,N,$$

Here, ${SA}_{i}$ is the set of safe areas for the ith lyrebird and $X_{k}$ is the kth row of X matrix, which has a better objective function value (i.e., $F_{k}$) than the ith LOA member (i.e., $F_{k}<F_{i}$).

In the LOA design, it is assumed that the lyrebird randomly escapes to one of these safe areas. Based on the lyrebird displacement modeling in this phase, a new position is calculated for each LOA member using Equation (6). Then, if the value of the objective function is improved, this new position replaces the previous position of the corresponding member according to Equation (7).

(6) $$x_{i,j}^{P1}=x_{i,j}+r_{i,j}\cdot (S{SA}_{i,j}-I_{i,j}\cdot x_{i,j}),$$

(7) $$X_{i}=\left\{\begin{array}{cc} X_{i}^{P1}, & F_{i}^{P1}\leq F_{i},\\ X_{i}, & else\;, \end{array}\right.$$

Here, $S{SA}_{i}$ is the selected safe area for ith lyrebird, $S{SA}_{i,j}$ is its jth dimension, $X_{i}^{P1}$ is the new position calculated for the ith lyrebird based on escaping strategy of the proposed LOA, $x_{i,j}^{P1}$ is its jth dimension, $F_{i}^{P1}$ is its objective function value, $r_{i,j}$ are random numbers from the interval $\left[0,\;1\right]$, and $I_{i,j}$ are numbers that are randomly selected as 1 or 2.

#### 3.3.2. Phase 2: Hiding Strategy (Exploitation Phase)

In this phase of LOA, the position of the population member is updated in the search space based on the modeling strategy of the lyrebird to hide in its surrounding safe area. Accurately scanning the surrounding environment and moving with small steps in order to reach a suitable area for hiding leads to small changes in the lyrebird’s position, which indicates the exploitation ability of LOA in local search.

In LOA design, based on the modeling of the lyrebird’s movement towards the near-suitable area for hiding, a new position is calculated for each LOA member using Equation (8). This new position replaces the previous position of the corresponding member if it improves the value of the objective function according to Equation (9).

(8) $$x_{i,j}^{P2}=x_{i,j}+\left(1-2r_{i,j}\right)\cdot \frac{ub_{j}-lb_{j}}{t}$$

(9) $$X_{i}=\left\{\begin{array}{cc} X_{i}^{P2}, & F_{i}^{P2}\leq F_{i}\\ X_{i}, & else \end{array}\right.$$

Here, $X_{i}^{P2}$ is the new position calculated for the ith lyrebird based on the hiding strategy of the proposed LOA, $x_{i,j}^{P2}$ is its jth dimension, $F_{i}^{P2}$ is its objective function value, $r_{i,j}$ are random numbers from the interval $\left[0,\;1\right]$, and t is the iteration counter.

### 3.4. Repetition Process, Pseudocode, and Flowchart of LOA

**Figure 2:**
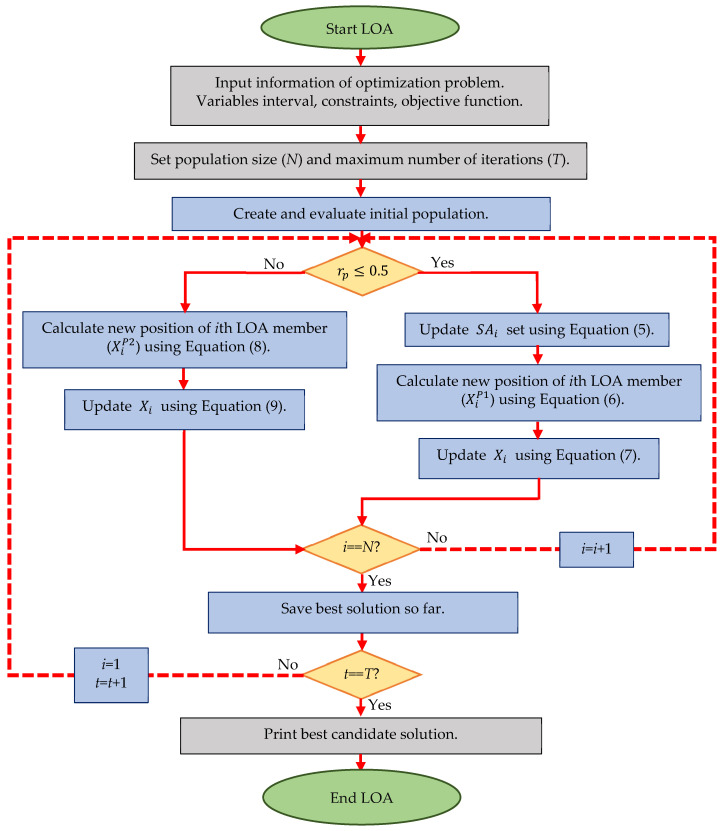
Flowchart of LOA.

By updating the position of all lyrebirds, the first LOA iteration is completed. Then the algorithm enters the next iteration and the process of updating the LOA population based on Equations (4)–(9) continues until the last iteration of the algorithm. In each iteration, the best candidate solution is updated and saved. After the full implementation of LOA, the best candidate solution stored during the iterations of the algorithm is output as a solution to the problem. The implementation steps of LOA are presented as a flowchart in Figure 2 and its pseudocode in Algorithm 1. Based on the LOA flowchart, the work process is as follows: first, the problem information about the objective function, constraints, and decision variables is placed in the input of the algorithm. Then the number of population members and the number of iterations required to solve the given problem are set. The first step is the random generation of the initial population of the algorithm and its evaluation in the objective function of the problem. After the initialization stage, the algorithm enters the first iteration. Then the process of updating the position of the first lyrebird in the problem-solving space starts. As described in LOA modeling, the lyrebird has two strategies when faced with danger: (i) escape and (ii) hide. In LOA design, it is assumed that with equal probability each lyrebird chooses one of these two strategies randomly based on Equation (4). If the lyrebird chooses the escape strategy, its position in the problem-solving space is updated based on Equations (5)–(7). If the lyrebird chooses the hiding strategy, its position in the problem-solving space is updated based on Equations (8) and (9). So far, the position of the first lyrebird (i.e., population member) is successfully updated. Then, the position of other lyrebirds is updated in the problem-solving space in the same way as the process mentioned for updating the first lyrebird. After updating the position of all lyrebirds in the problem-solving space, at this stage, the first iteration of the algorithm is completed. Based on the comparison of the evaluated values for the objective function, the best candidate solution is obtained until this iteration is saved. Then the algorithm enters the next iteration and the process of updating lyrebirds in the problem-solving space proceeds similarly to the process mentioned for the first iteration and continues until the last iteration of the algorithm. After completing all the iterations of the algorithm, the best solution obtained during the iterations of the algorithm is placed in the output as a solution for the given problem. Here, the implementation of the algorithm ends successfully.
**Algorithm 1.** Pseudocode of LOA.Start LOA.1.Input problem information: variables, objective function, and constraints.2.Set LOA population size (*N*) and iterations (*T*).3.Generate the initial population matrix at random using Equation (2). $x_{i,d}\leftarrow lb_{d}+r\cdot (ub_{d}-lb_{d})$4.Evaluate the objective function.5.Determine the best candidate solution.6. **For ** t=1 **to** ***T***7.  **For** i=1 **to** N8.  Determine the type of lyrebird defense strategy against predator attack using Equation (4). $X_{i}\leftarrow \left\{\begin{array}{c} based\;on\;Phase\;1,\;\;r_{p}\leq 0.5\\ based\;on\;Phase\;2,\;\;else\; \end{array}\right.$9.   **if** $r_{p}\leq 0.5$ **(chose Phase 1)**10.   Determine candidate safe areas for *i*th lyrebird based on Equation (5). ${SA}_{i}\leftarrow \left\{X_{k},\;F_{k}<F_{i}\;\;\mathrm{and}\;k\;\in \;\left\{1,2,..,N\right\}\right\}$11.   Calculate the new position of the ith LOA member using Equation (6). $x_{i,j}^{P1}\leftarrow x_{i,j}+r_{i,j}\;\cdot (S{SA}_{i,j}-I_{i,j}\cdot x_{i,j}),\;\;$12.    Update *i*th LOA member using Equation (7). $X_{i}\leftarrow \left\{\begin{array}{cc} X_{i}^{P1}, & F_{i}^{P1}<F_{i}\\ X_{i}, & else \end{array}\right.$13.
   **else (chose Phase 2)**14.    Calculate the new position of the ith LOA member using Equation (8). $x_{i,j}^{P2}\leftarrow x_{i,j}+\left(1-2\;r_{i,j}\right)\cdot \;\frac{ub_{j}-lb_{j}}{t}\;\;$15.   Update *i*th LOA member using Equation (9). $X_{i}\leftarrow \left\{\begin{array}{cc} X_{i}^{P2}, & F_{i}^{P2}<F_{i}\\ X_{i}, & else \end{array}\right.$16.   **end (if)**17.  **end (For ** i=1 **to** N**)**18.  Save the best candidate solution so far.19. **end (For ** t=1 **to *T*)**20. Output the best quasi-optimal solution obtained with the LOA.End LOA.

### 3.5. Computational Complexity of LOA

In this subsection, the computational complexity of the proposed LOA approach, including the time complexity and space complexity, is evaluated.

The time complexity of LOA is affected by the initialization process, calculating the objective function, and population updating as follows:

- The preparation and initialization steps of LOA have a time complexity equal to *O*(*Nm*), where *N* is the number of lyrebirds in the population and *m* is the number of decision variables of the problem.
- In each iteration, the objective function is calculated corresponding to each lyrebird. Therefore, calculating the objective function has a time complexity equal to *O*(*NT*), where *T* is the maximum number of LOA iterations.
- In each iteration, each lyrebird is randomly updated based on one of the escape or hiding phases. Therefore, the lyrebird update process has a time complexity equal to *O*(*NmT*).

Therefore, the total time complexity of the proposed LOA approach is equal to *O*(*N*(*T*(1 + *m*) + *m*)), which can be simplified to *O*(*Nm*(1 + *T*)).

The space complexity of the LOA is *O*(*Nm*), which is considered the maximum amount of space in its initialization process.

## 4. Simulation Studies and Results

In this section, the capability of the proposed LOA approach was tested in dealing with optimization issues. In this regard, the CEC 2017 test suite was employed for problem dimensions of 10, 30, 50, and 100.

### 4.1. Performance Comparison and Experimental Setting

With the aim of measuring the effectiveness of LOA in providing suitable solutions for optimization problems, the results obtained from it were compared with the performance of twelve famous metaheuristic algorithms: GA [44], PSO [25], GSA [52], TLBO [64], MVO [54], GWO [29], WOA [42], MPA [33], TSA [41], RSA [35], AVOA [39], and WSO [37]. The values of the control parameters of the metaheuristic algorithms are specified in Table 1. In handling the CEC 2017 test suite, the LOA approach and each of the competitor algorithms were implemented in 51 independent runs, where each independent run included 10,000·m (m is the number of variables) of FEs and a population size of 30. The simulation results are reported using six statistical indicators: mean, best, worst, standard deviation (std), median, and rank. In order to rank the metaheuristic algorithms in handling each of the benchmark functions, the mean criterion is used.

### 4.2. Evaluation CEC 2017 Test Suite

In this subsection, the performance of LOA and competitor algorithms was evaluated in solving the CEC 2017 test suite for problem dimensions equal to 10, 30, 50, and 100. The CEC 2017 test suite has thirty benchmark functions consisting of (i) three unimodal functions of C17-F1 to C17-F3, (ii) seven multimodal functions of C17-F4 to C17-F10, (iii) ten hybrid functions of C17-F11 to C17-F20, and (iv) ten composition functions of C17-F21 to C17-F30. From this test suite, function C17-F2 was excluded from the simulation studies due to its unstable behavior. Comprehensive and detailed information on the CEC 2017 test suite is available in [90].

**Figure 3:**
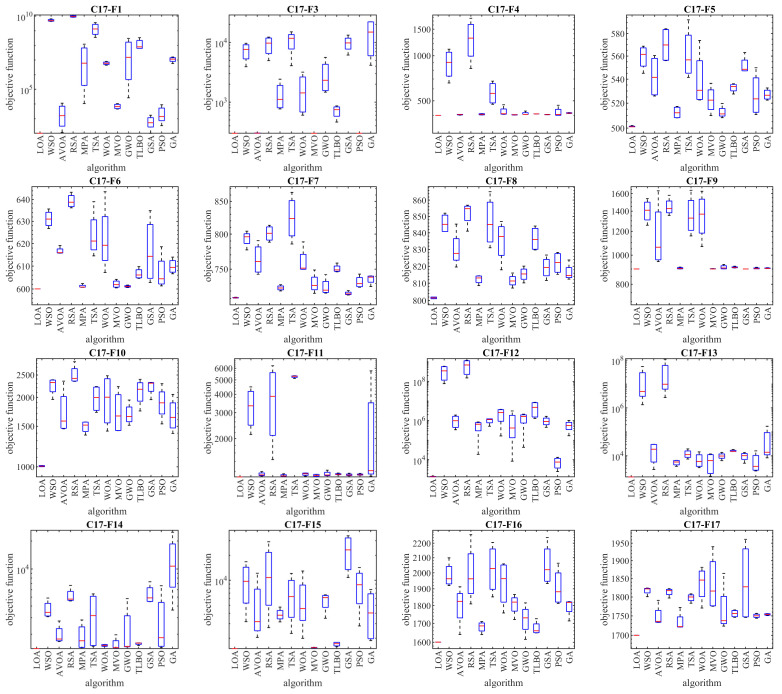

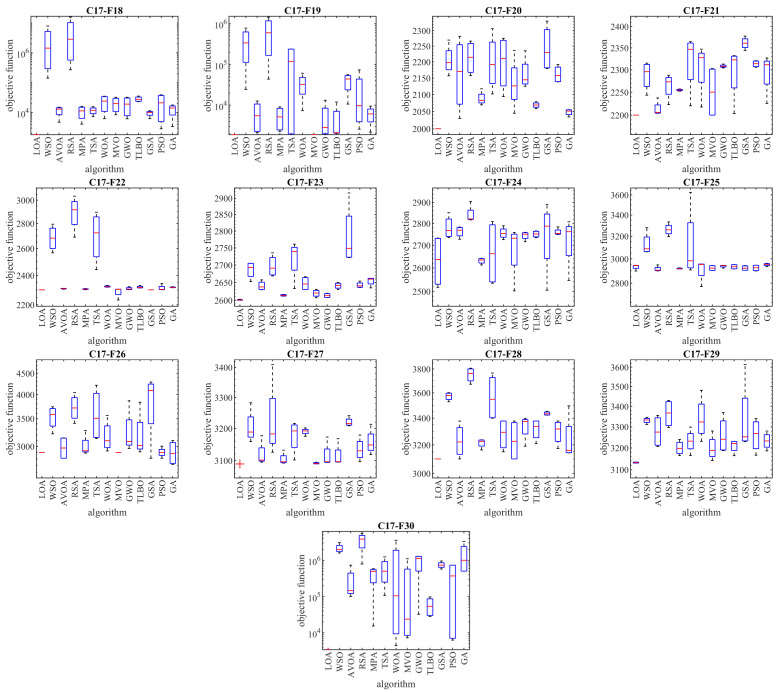
Boxplot diagrams of LOA and competitor algorithm performances on CEC 2017 test suite (dimension = 10).

**Figure 4:**
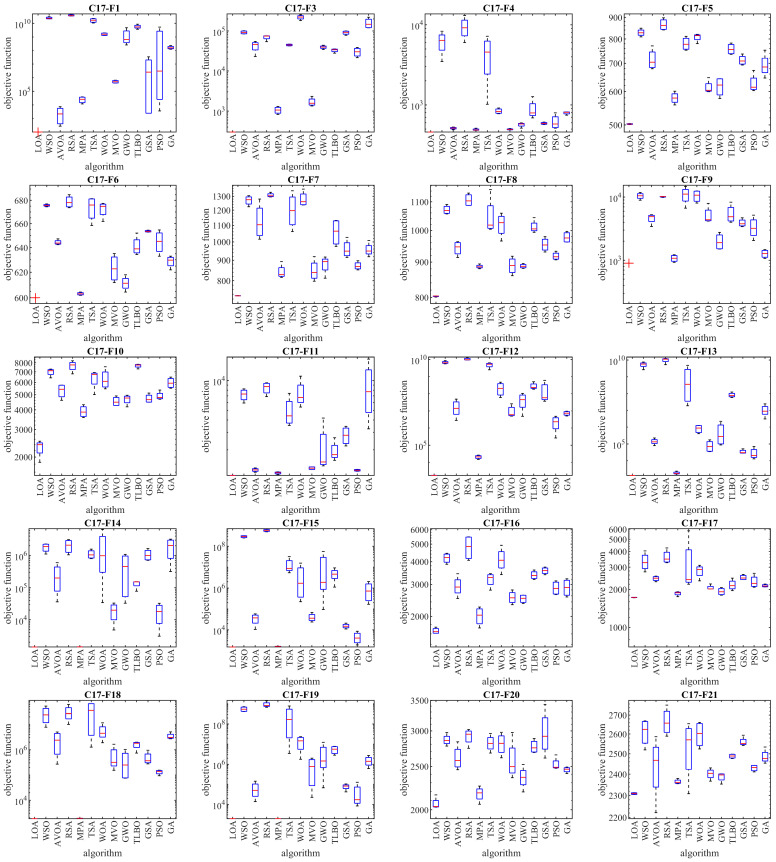

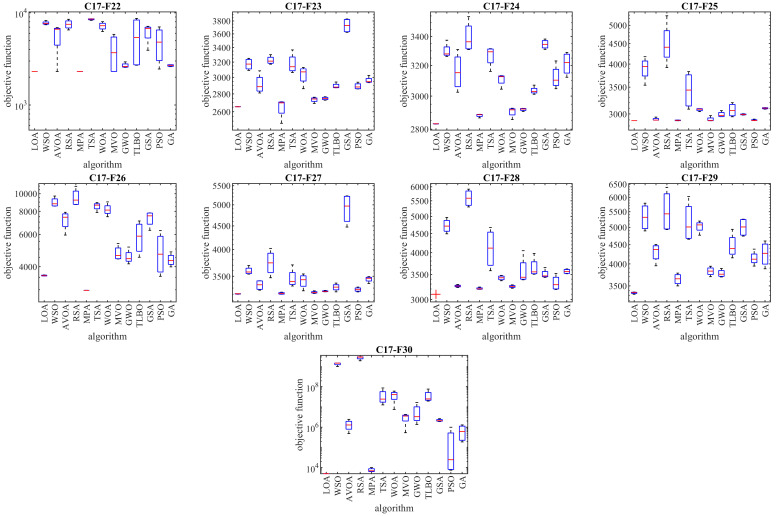
Boxplot diagrams of LOA and competitor algorithm performances on CEC 2017 test suite (dimension = 30).

**Figure 5:**
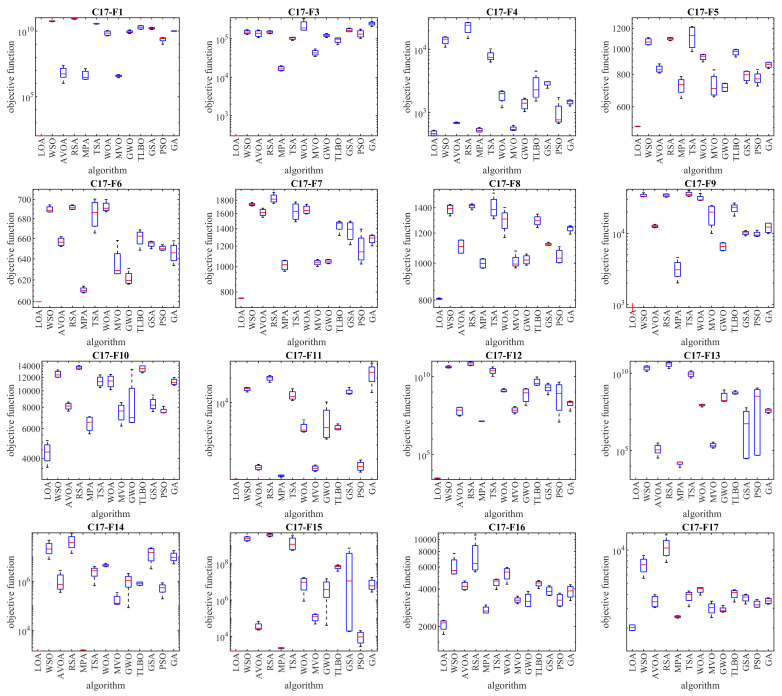

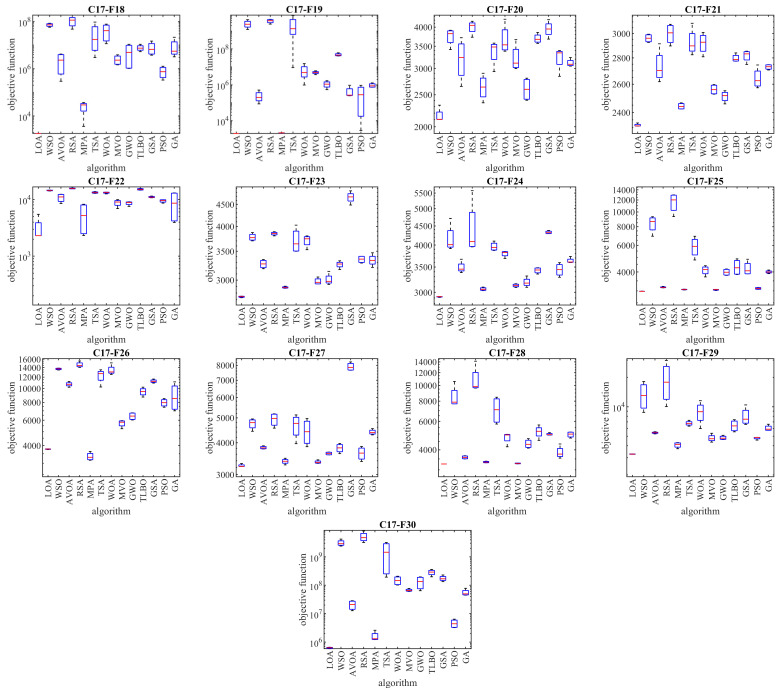
Boxplot diagrams of LOA and competitor algorithm performances on CEC 2017 test suite (dimension = 50).

**Figure 6:**
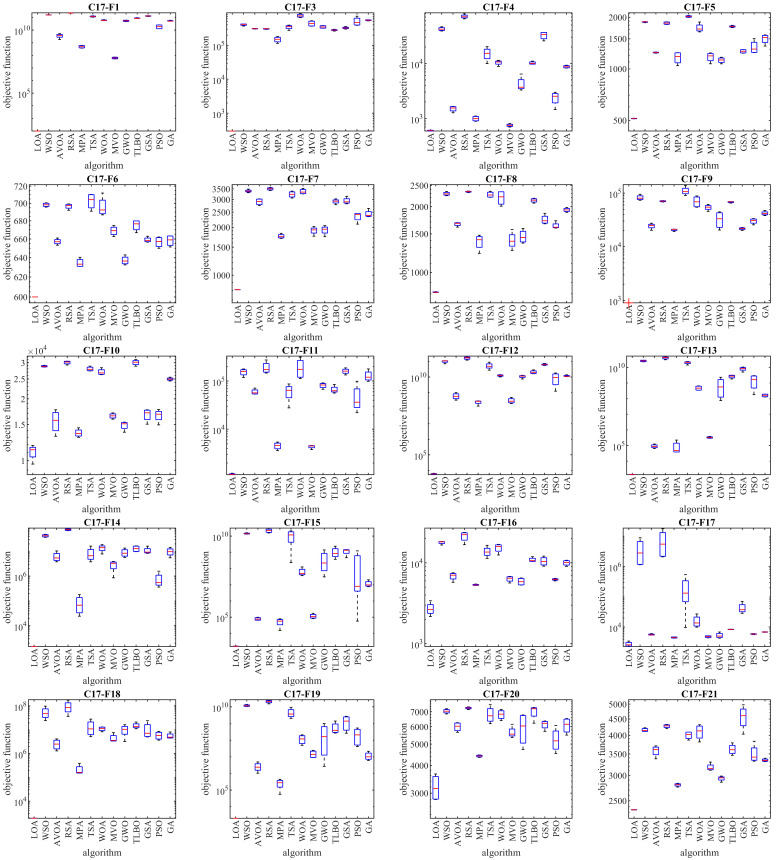

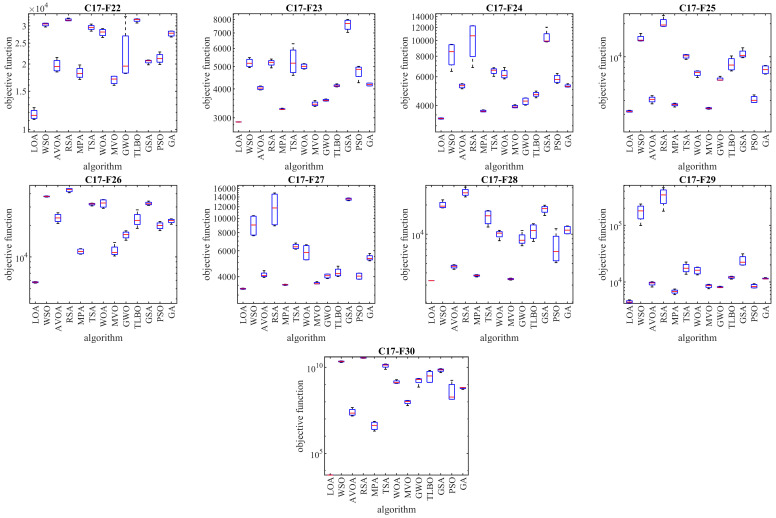
Boxplot diagrams of LOA and competitor algorithm performances on CEC 2017 test suite (dimension = 100).

The optimization results of the CEC 2017 test suite using the proposed LOA approach and competitor algorithms are reported in Table 2, Table 3, Table 4 and Table 5. The boxplot diagrams extracted from the performance of the metaheuristic algorithms are plotted in Figure 3, Figure 4, Figure 5 and Figure 6. Based on the obtained simulation results, in handling the CEC 2017 test suite for the problem dimension equal to 10 (*m* = 10), the proposed LOA approach is the first best optimizer in order to solve the functions C17-F1, C17-F3 to C17-F21, C17-F23, C17-F24, and C17-F27 to C17-F30. For a problem dimension equal to 30 (*m* = 30), the proposed LOA approach is the first best optimizer for functions C17-F1, C17-F3 to C17-F22, C17-F24, C17-F25, and C17-F27 to C17-F30. For a problem dimension equal to 50 (*m* = 50), the proposed LOA approach is the first best optimizer for functions C17-F1, C17-F3 to C17-F25, and C17-F27 to C17-F30. For a problem dimension equal to 100 (*m* = 100), the proposed LOA approach is the first best optimizer for functions C17-F1, and C17-F3 to C17-F30.

What can be concluded from the optimization results is that LOA is able to provide an effective solution for the CEC 2017 test suite with proper efficiency in exploration, exploitation, and balancing them during the search process. The simulation results show that LOA provides better results in most of the benchmark functions and obtains the rank of the first best optimizer, indicating superior performance compared to the competitor algorithms in handling the CEC 2017 test suite for problem dimensions equal to 10, 30, 50, and 100.

### 4.3. Statistical Analysis

In this subsection, we investigated whether the proposed LOA approach has a significant statistical superiority compared to the competitor algorithms or not by implementing an analytical analysis on the results obtained from the metaheuristic algorithms. In this regard, the Wilcoxon rank sum test [91], which is a non-parametric test used to determine the significant difference between the averages of two data samples, was employed. In this test, the presence or absence of a significant difference is determined using a criterion called *p*-value.

The results of applying the Wilcoxon rank sum test on the performance of LOA and competitor algorithms are reported in Table 6. Based on the results obtained from the statistical analysis, in cases where the *p*-value is less than 0.5, the proposed LOA approach has a significant statistical superiority in competition with the corresponding algorithms. The Wilcoxon rank sum test results shows that LOA has significant statistical superiority in handling the CEC 2017 test suite for problem dimensions equal to 10, 30, 50, and 100 compared to all twelve competitor algorithms.

### 4.4. Discussion

The proposed LOA approach is a population-based meta-heuristic algorithm of random problem-solving approaches for solving optimization problems. LOA is able to provide suitable solutions for optimization problems in an iteration-based process based on the searching power of its members in the problem-solving space. In order to manage an optimal random search process, LOA must have adequate power in exploitation, exploration, and also balancing them during the search process.

Because unimodal functions lack local optima, they are suitable options for evaluating the exploitation ability of metaheuristic algorithms in local search management. The C17-F1 and C17-F3 functions in the CEC 2017 test suite are the unimodal type. Based on the optimization results of these functions for problem dimensions equal to 10, 30, 50, and 100 reported in Table 2, Table 3, Table 4 and Table 5, LOA was the first best optimization for these functions. An analysis of the simulation results shows that LOA provided superior performance in competition with the competitor algorithms by achieving better results in handling unimodal functions. The analysis of the simulation results and the performance of metaheuristic algorithms in handling unimodal functions shows that LOA with high exploitation ability was able to search the problem-solving space well at the local level and achieve suitable solutions for these functions.

Because multimodal functions have several local optima, they are suitable options to evaluate the exploration ability of metaheuristic algorithms in global search management. Functions C17-F4 to C17-F10 in the CEC 2017 test suite are of the multimodal type. Based on the optimization results of these functions presented in Table 2, Table 3, Table 4 and Table 5, LOA provided effective performance in handling multimodal functions for problem dimensions equal to 10, 30, 50, and 100. Based on the simulation results, it is evident that LOA provided superior performance compared to the competitor algorithms by providing better results for solving multimodal functions. What is evident from the analysis of the simulation results and the performance of the metaheuristic algorithms in handling multimodal functions is that LOA, with its high exploration ability, is able to manage the global search in the problem-solving space well, and while avoiding becoming stuck in local optima, it can achieve suitable solutions for optimization problems.

Functions C17-F11 to C17-F30 in the CEC 2017 test suite were selected from the type of complex optimization problems in order to evaluate the ability of metaheuristic algorithms in balancing exploration and exploitation during the search process in the problem-solving space. The simulation results of the C17-F11 to C17-F30 functions, which are reported in Table 2, Table 3, Table 4 and Table 5, show that LOA, by creating a balance between exploration and exploitation, firstly by managing the global search was able to find the main region containing the main optimum, and secondly by managing local search converged towards suitable solutions close to the global optimum. Based on the optimization results of these functions for problem dimensions equal to 10, 30, 50, and 100, LOA was the first best optimizer in most of these functions. What is concluded from the analysis of the simulation results is that LOA, with high ability in balancing exploration and exploitation, provided superior performance in handling the complex optimization problems C17-F4 to C17-F30 in competition with competitor algorithms.

The main findings from the analysis of the simulation results are that LOA has high ability in exploitation in managing the local search based on the optimization results of the C17-F1 and C17-F3 functions, has high ability in exploration in managing the global search based on the optimization results of the functions C17-F4 to C17-F10, and has a high ability to balance exploration and exploitation based on the optimization results of the C17-F11 to C17-F30 functions.

Although the analysis and comparison of the performance of metaheuristic algorithms using statistical indicators provides valuable information, the statistical analysis clearly shows whether the superiority of an algorithm over other algorithms is significant from a statistical point of view. The results of the statistical analysis obtained from the Wilcoxon rank sum test reported in Table 6 confirm that the proposed LOA approach has significant statistical superiority in handling the CEC 2017 test suite for problem dimensions equal to 10, 30, 50, and 100 in competition with all twelve competitor algorithms.

## 5. LOA for Real-World Applications

In this section, the capability of the proposed LOA approach in solving optimization tasks in real-world applications is challenged. For this purpose, twenty-two real-world constrained optimization problems from the CEC 2011 test suite and four engineering design problems were selected.

### 5.1. Evaluation of CEC 2011 Test Suite

In this subsection, the effectiveness of LOA and competitor algorithms in handling the CEC 2011 test suite is analyzed. This test suite consists of twenty-two constrained optimization problems from real-world applications. A full description and detailed details of the CEC 2011 test suite are provided in [92].

**Figure 7:**
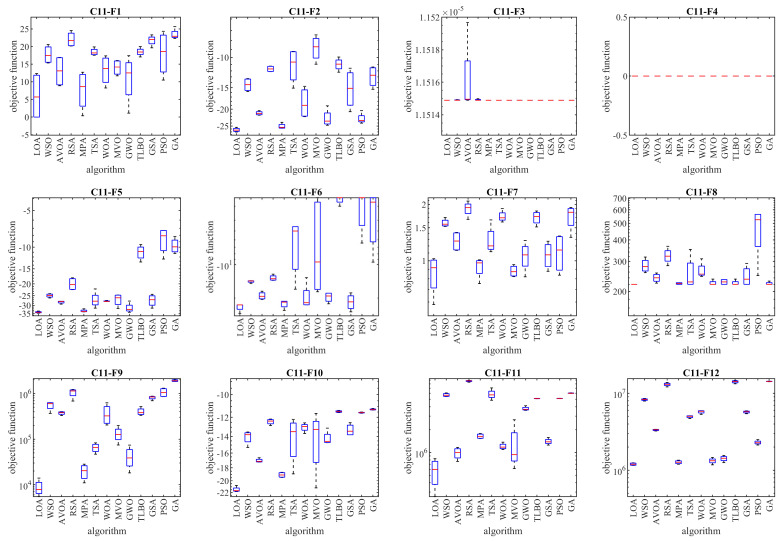

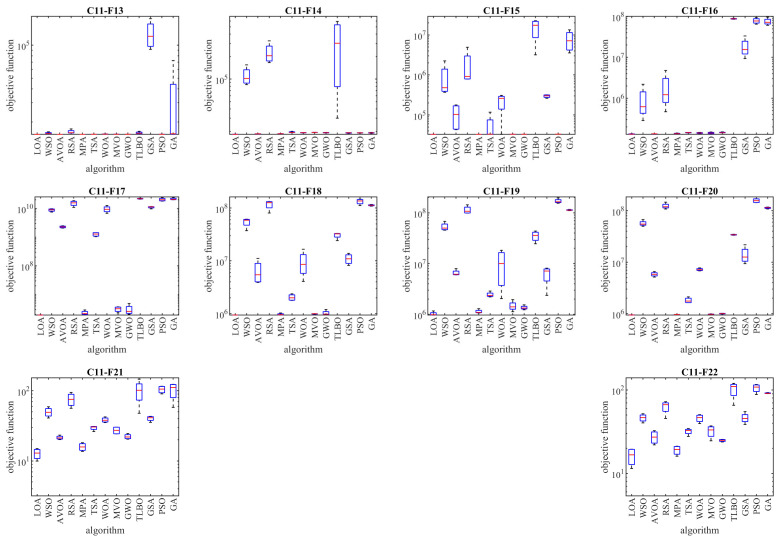
Boxplot diagrams of LOA and competitor algorithms performances on CEC 2011 test suite.

The optimization results of the CEC 2011 test suite using LOA and competitor algorithms are reported in Table 7. The boxplot diagrams obtained from the performance of the mentioned algorithms are plotted in Figure 7. Based on the optimization results, it is evident that the LOA, with high ability in exploration, exploitation, and balancing them during the search process was able to provide effective solutions for the CEC 2011 test suite. Also, the analysis of the simulation results shows that LOA, by providing better results to solve the optimization problems C11-F1 to C11-F22 and in total becoming the rank of the first best optimizer, provided superior performance compared to the competitor algorithms. The results obtained from the Wilcoxon rank sum test confirm that LOA has significant statistical superiority compared to the competitor algorithms in handling the CEC 2011 test suite.

### 5.2. Pressure Vessel Design Problem

**Figure 8:**
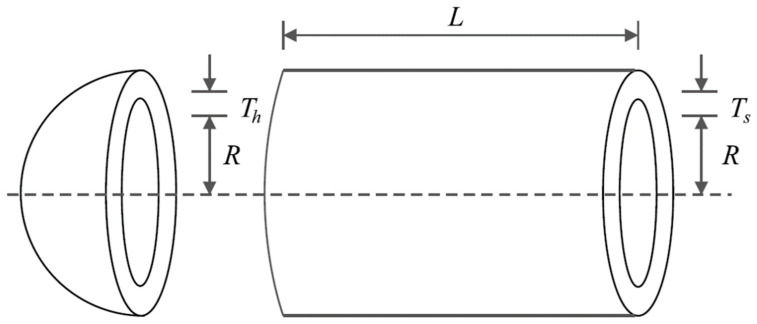
Schematic of pressure vessel design.

Pressure vessel design is a real-world application with the schematic shown in Figure 8, where the main goal in this design is to minimize construction cost. The mathematical model of pressure vessel design is as follows [93]:

*Consider: $X=\left[x_{1},\;x_{2},\;x_{3},\;x_{4}\right]=\left[T_{s},\;T_{h},\;R,\;L\right]$.*

*Minimize:* $f\left(x\right)=0.6224x_{1}x_{3}x_{4}+1.778x_{2}x_{3}^{2}+3.1661x_{1}^{2}x_{4}+19.84x_{1}^{2}x_{3}.$

*Subject to:*

$$g_{1}\left(x\right)=-x_{1}+0.0193x_{3}\;\leq \;0,\;g_{2}\left(x\right)=-x_{2}+0.00954x_{3}\leq \;0,$$

$$g_{3}\left(x\right)=-\pi x_{3}^{2}x_{4}-\frac{4}{3}\pi x_{3}^{3}+1296000\leq \;0,\;g_{4}\left(x\right)=x_{4}-240\;\leq \;0.$$

with

$$0\leq x_{1},x_{2}\leq 100\;{\mathrm{and}\;10\leq x}_{3},x_{4}\leq 200.$$

**Figure 9:**
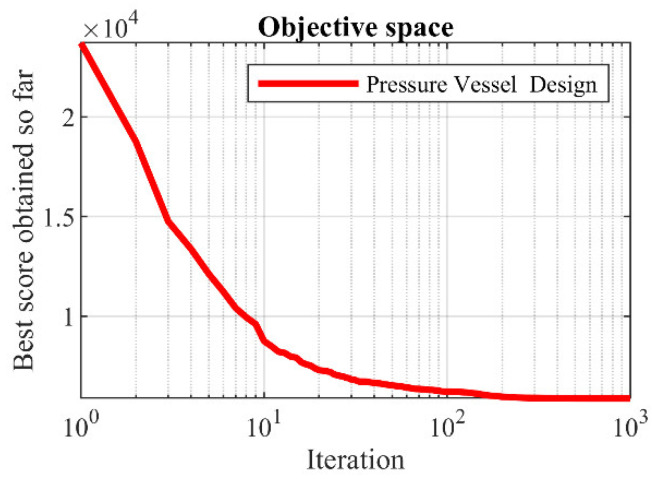
LOA’s performance convergence curve on pressure vessel design.

The optimization results of pressure vessel design using the proposed LOA approach and competitor algorithms are reported in Table 8 and Table 9. The convergence curve of LOA during pressure vessel design optimization is presented in Figure 9. Based on the simulation results, LOA provided the optimal design with design variable values equal to (0.7780271, 0.3845792, 40.312284, 200) and the value of the objective function equal to (5882.9013). An analysis of the simulation results shows that LOA provided more effective performance in pressure vessel design by providing better results for design variables and statistical indicators.

### 5.3. Speed Reducer Design Problem

**Figure 10:**
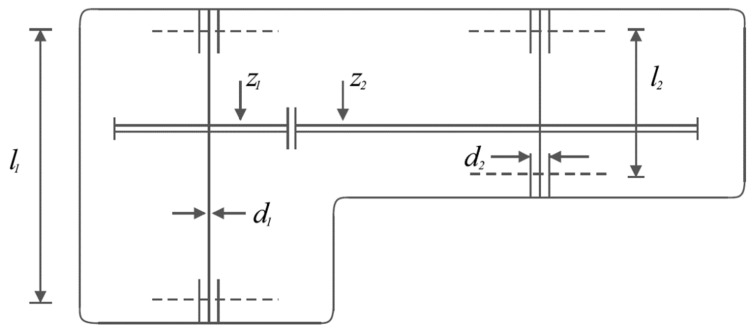
Schematic of speed reducer design.

Speed reducer design is a real-world subject with the schematic shown in Figure 10, where the main goal in this design is to minimize the weight of the speed reducer. The mathematical model of speed reducer design is as follows [94,95]:

*Consider*: $X=\left[x_{1,}x_{2},x_{3},x_{4},x_{5}{,x}_{6},x_{7}\right]=\left[b,m,p,l_{1},l_{2},d_{1},d_{2}\right]$.

*Minimize*: $f\left(x\right)=0.7854x_{1}x_{2}^{2}\left(3.3333x_{3}^{2}+14.9334x_{3}-43.0934\right)-1.508x_{1}\left(x_{6}^{2}+x_{7}^{2}\right)+7.4777\left(x_{6}^{3}+x_{7}^{3}\right)+0.7854(x_{4}x_{6}^{2}+x_{5}x_{7}^{2})$.

*Subject to*:

$$g_{1}\left(x\right)=\frac{27}{x_{1}x_{2}^{2}x_{3}}-1\leq 0,g_{2}\left(x\right)=\frac{397.5}{x_{1}x_{2}^{2}x_{3}}-1\leq 0,$$

$$g_{3}\left(x\right)=\frac{1.93x_{4}^{3}}{x_{2}x_{3}x_{6}^{4}}-1\leq 0,g_{4}\left(x\right)=\frac{1.93x_{5}^{3}}{x_{2}x_{3}x_{7}^{4}}-1\leq 0,$$

$$g_{5}\left(x\right)=\frac{1}{110x_{6}^{3}}\sqrt{{\left(\frac{745x_{4}}{x_{2}x_{3}}\right)}^{2}+16.9\times 10^{6}}-1\leq 0,$$

$$g_{6}(x)=\frac{1}{85x_{7}^{3}}\sqrt{{\left(\frac{745x_{5}}{x_{2}x_{3}}\right)}^{2}+157.5\times 10^{6}}-1\leq 0,$$

$$g_{7}\left(x\right)=\frac{x_{2}x_{3}}{40}-1\leq 0,g_{8}\left(x\right)=\frac{{5x}_{2}}{x_{1}}-1\leq 0,$$

$$g_{9}\left(x\right)=\frac{x_{1}}{12x_{2}}-1\leq 0,g_{10}\left(x\right)=\frac{{1.5x}_{6}+1.9}{x_{4}}-1\leq 0,$$

with

$$2.6\leq x_{1}\leq 3.6,0.7\leq x_{2}\leq 0.8,17\leq x_{3}\leq 28,7.3\leq x_{4}\leq 8.3,7.8\leq x_{5}\leq 8.3,2.9\leq x_{6}\leq 3.9,\mathrm{and}5\leq x_{7}\leq 5.5.$$

**Figure 11:**
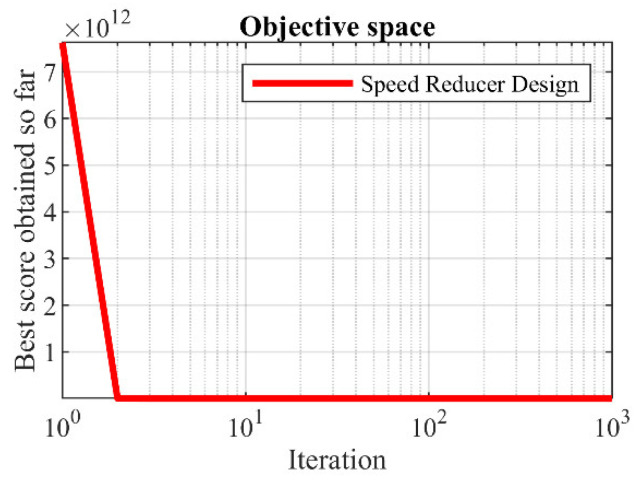
LOA’s performance convergence curve on speed reducer design.

The results of using the proposed LOA approach and competitor algorithms in dealing with the speed reducer design problem are published in Table 10 and Table 11. The convergence process of LOA towards the optimal solution for the speed reducer design problem is presented in Figure 11. Based on the simulation results, LOA provided the optimal design with the design variable values equal to (3.5, 0.7, 17, 7.3, 7.8, 3.3502147, 5.2866832) and the value of the objective function equal to (2996.3482). What is evident from the comparison of the simulation results is that LOA, by providing better results while obtaining the rank of the first best optimizer, provided superior performance compared to the competitor algorithms in optimizing speed reducer design.

### 5.4. Welded Beam Design

**Figure 12:**
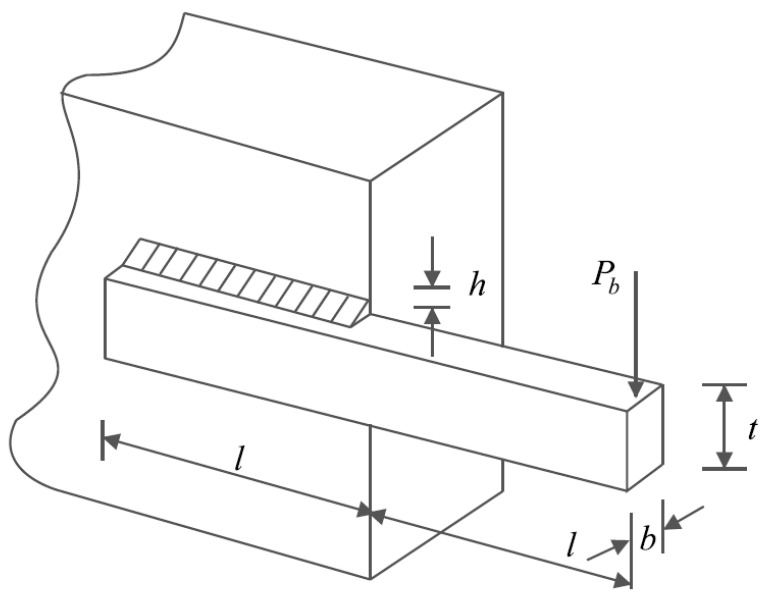
Schematic of welded beam design.

Welded beam design is a real-world constrained optimization problem with the schematic shown in Figure 12, where the main goal in this design is to minimize the fabrication cost of the welded beam. The mathematical model of welded beam design is as follows [42]:

*Consider: $X=\left[x_{1},\;x_{2},\;x_{3},\;x_{4}\right]=\left[h,\;l,\;t,\;b\right]$.*

*Minimize: $f(x)=1.10471x_{1}^{2}x_{2}+0.04811x_{3}x_{4}\;(14.0+x_{2})$.*

*Subject to:*

$$g_{1}\left(x\right)=\tau \left(x\right)-13600\;\leq \;0,\;g_{2}\left(x\right)=\sigma \left(x\right)-30000\;\leq \;0,$$

$$g_{3}\left(x\right)=x_{1}-x_{4}\leq \;0,\;g_{4}(x)=0.10471x_{1}^{2}+0.04811x_{3}x_{4}\;(14+x_{2})-5.0\;\leq \;0,$$

$$g_{5}\left(x\right)=0.125-x_{1}\leq \;0,\;g_{6}\left(x\right)=\delta \;\left(x\right)-0.25\;\leq \;0,$$

$$g_{7}\left(x\right)=6000-p_{c}\;\left(x\right)\leq \;0.$$

*where*

$$\tau \left(x\right)=\sqrt{{\left(\tau^{\prime }\right)}^{2}+\left(2\tau \tau^{\prime }\right)\frac{x_{2}}{2R}+{\left(\tau^{\prime \prime }\right)}^{2}\;},\;\tau^{\prime }=\frac{6000}{\sqrt{2}x_{1}x_{2}},\;\tau^{\prime \prime }=\frac{MR}{J},$$

$$M=6000\left(14+\frac{x_{2}}{2}\right),\;R=\sqrt{\frac{x_{2}^{2}}{4}+{\left(\frac{x_{1}+x_{3}}{2}\right)}^{2}},$$

$$J=2\left\{x_{1}x_{2}\sqrt{2}\left[\frac{x_{2}^{2}}{12}+{\left(\frac{x_{1}+x_{3}}{2}\right)}^{2}\right]\right\}\;,\;\sigma \left(x\right)=\frac{504000}{x_{4}x_{3}^{2}}\;,$$

$$\delta \;\left(x\right)=\frac{65856000}{\left(30\cdot 10^{6}\right)x_{4}x_{3}^{3}}\;,\;p_{c}\;\left(x\right)=\frac{4.013\left(30\cdot 10^{6}\right)\sqrt{\frac{x_{3}^{2}x_{4}^{6}}{36}}}{196}\left(1-\frac{x_{3}}{28}\sqrt{\frac{30\cdot 10^{6}}{4(12\cdot 10^{6})}}\right)\;.$$

with

$$0.1\leq x_{1},\;x_{4}\leq 2\;\mathrm{and}0.1\leq x_{2},\;x_{3}\leq 10.$$

**Figure 13:**
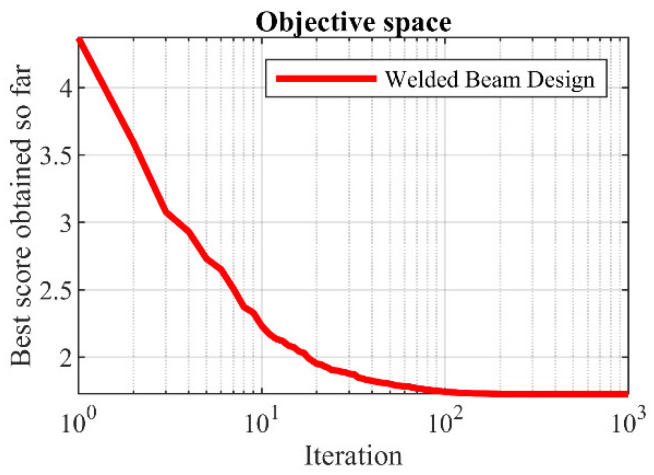
LOA’s performance convergence curve on welded beam design.

The results of employing the proposed LOA approach and competitor algorithms to deal with welded beam design are presented in Table 12 and Table 13. The convergence curve of LOA while achieving the optimal design for welded beam problem is drawn in Figure 13. Based on the simulation results, LOA has provided the optimal design with the design variable values equal to (0.2057296, 3.4704887, 9.0366239, 0.2057296) and the value of the objective function equal to (1.7246798). The comparison of the simulation results indicates that LOA, by providing better results for design variables and statistical indicators, provided superior performance compared to competitor algorithms in addressing welded beam design.

### 5.5. Tension/Compression Spring Design

**Figure 14:**
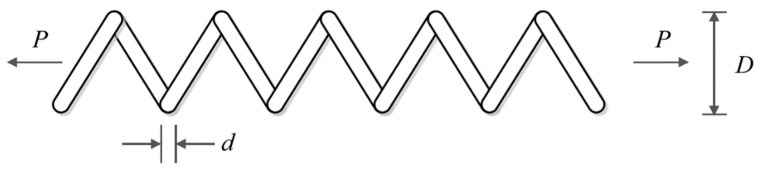
Schematic of tension/compression spring design.

Tension/compression spring design is a real-world application with the schematic shown in Figure 14, where the main goal in this design is to minimize the weight of a tension/compression spring. The mathematical model of a tension/compression spring design is as follows [42]:

*Consider:* $X=\left[x_{1},\;x_{2},\;x_{3}\;\right]=\left[d,\;D,\;P\right].$

*Minimize:* $f\left(x\right)=\left(x_{3}+2\right)x_{2}x_{1}^{2}.$

*Subject to:*

$$g_{1}\left(x\right)=1-\frac{x_{2}^{3}x_{3}}{71785x_{1}^{4}}\;\leq \;0,\;g_{2}\left(x\right)=\frac{4x_{2}^{2}-x_{1}x_{2}}{12566(x_{2}x_{1}^{3})}+\frac{1}{5108x_{1}^{2}}-1\leq \;0,$$

$$g_{3}\left(x\right)=1-\frac{140.45x_{1}}{x_{2}^{2}x_{3}}\leq \;0,g_{4}\left(x\right)=\frac{x_{1}+x_{2}}{1.5}-1\;\leq \;0.$$

with

$$0.05\leq x_{1}\leq 2,\;{0.25\leq x}_{2}\leq 1.3\;\mathrm{and}\;2\leq \;x_{3}\leq 15$$

**Figure 15:**
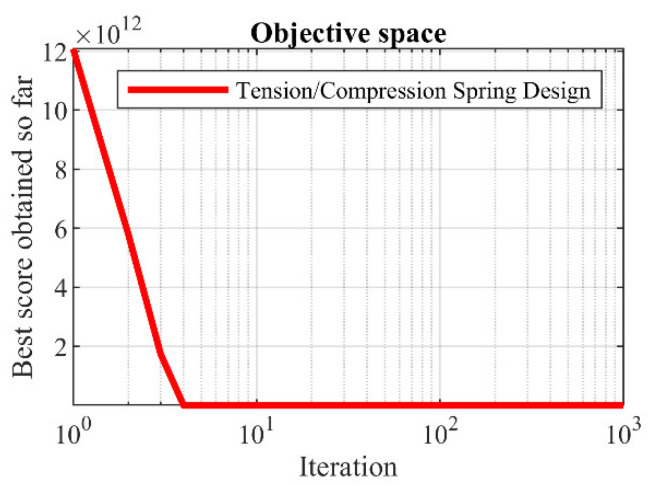
LOA’s performance convergence curve on tension/compression spring.

The implementation results of the proposed LOA approach and competitor algorithms on the tension/compression spring design problem are reported in Table 14 and Table 15. The convergence process of LOA while achieving the optimal design for the tension/compression spring problem is shown in Figure 15. Based on the simulation results, LOA provided the optimal design with the design variable values equal to (0.0516891, 0.3567177, 11.288966) and the value of the objective function equal to (0.0126019). What can be concluded from the simulation results is that the proposed LOA approach, by providing better results, provided superior performance compared to competitor algorithms in dealing with the tension/compression spring design problem.

## 6. Conclusions and Future Works

In this paper, a new bio-inspired metaheuristic algorithm named the Lyrebird Optimization Algorithm (LOA) that imitates the natural behavior of lyrebirds in the wild was introduced. The fundamental inspiration of LOA is derived from the strategy of lyrebirds when faced with danger, where this bird decides to run away or hide somewhere by examining its surroundings. LOA theory was expressed and mathematically modeled in two phases: (i) exploration based on simulation of the lyrebird escape strategy and (ii) exploitation based on simulation of the lyrebird hiding strategy. The efficiency of LOA in dealing with optimization tasks was challenged in order to optimize the CEC 2017 test suite for problem dimensions equal to 10, 30, 50, and 100. The optimization results indicate the high power of LOA in managing exploration, exploitation, and balancing them in the search process. In order to measure the quality of LOA in optimization, its performance was compared with the performance of twelve well-known metaheuristic algorithms. The simulation results show that LOA has superior performance compared to competitor algorithms by providing better results in most of the benchmark functions. A statistical analysis shows that this superiority of LOA is also significant from a statistical point of view. In addition, the implementation of LOA on twenty-two constrained optimization problems from the CEC 2011 test suite and four engineering design problems shows the capability of the proposed approach in handling real-world applications.

After the introduction of LOA, several research tasks are proposed for future studies. The development of binary and multi-objective versions of the proposed LOA approach are the most significant suggestions of this study for future work. Employing LOA in order to solve optimization problems in different sciences and real-world applications are other research potentials of the proposed approach for future works.

## Figures and Tables

**Table 1:** Control parameter values.

Algorithm	Parameter	Value
GA		
	Type	Real coded
	Selection	Roulette wheel (proportionate)
	Crossover	Whole arithmetic (probability = 0.8, $\alpha \in \left[-0.5,\;1.5\right]$)
	Mutation	Gaussian (probability = 0.05)
PSO		
	Topology	Fully connected
	Cognitive and social constant	(*C*_1_, *C*_2_) =(2, 2)
	Inertia weight	Linear reduction from 0.9 to 0.1.
	Velocity limit	10% of dimension range
GSA		
	Alpha, *G*_0_, *R_norm_*, *R_power_*	20, 100, 2, 1
TLBO		
	*T_F_*: teaching factor	*T_F_* = round $\left[\left(1+rand\right)\right]$
	random number	*rand* is a random number between $\left[0–1\right].$
GWO		
	Convergence parameter (*a*)	*a*: linear reduction from 2 to 0.
MVO		
	Wormhole existence probability (WEP)	Min(WEP) = 0.2 and Max(WEP) = 1.
	Exploitation accuracy over the iterations (*p*)	p=6.
WOA		
	Convergence parameter (*a*)	*a*: linear reduction from 2 to 0.
	*r* is a random vector in $\left[0–1\right].$	
	*l* is a random number in $\left[-1,1\right].$	
TSA		
	P_min_ and P_max_	1, 4
	*c1, c2, c3*	Random numbers lie in the range of $\left[0–1\right].$
MPA		
	Constant number	*P* = 0.5
	Random vector	*R* is a vector of uniform random numbers in $\left[0,\;1\right].$
	Fish Aggregating Devices (*FADs*)	*FADs* = 0.2
	Binary vector	*U* = 0 or 1
RSA		
	Sensitive parameter	β=0.01
	Sensitive parameter	α=0.1
	Evolutionary Sense (ES)	ES: randomly decreasing values between 2 and −2.
AVOA		
	L_1_, L_2_	0.8, 0.2
	w	2.5
	P_1_, P_2_, P_3_	0.6, 0.4, 0.6
WSO		
	F_min_ and F_max_	0.07, 0.75
	*τ*, *a_o_*, *a*_1_, *a*_2_	4.125, 6.25, 100, 0.0005

**Table 2:** Optimization results of CEC 2017 test suite (dimension = 10).

		LOA	WSO	AVOA	RSA	MPA	TSA	WOA	MVO	GWO	TLBO	GSA	PSO	GA
C17-F1	Mean	1.00E+02	5.18E+09	3.66E+03	9.70E+09	3.35E+07	1.65E+09	6.13E+06	7.15E+03	8.38E+07	1.40E+08	7.14E+02	2.99E+03	1.13E+07
	Best	1.00E+02	4.35E+09	1.15E+02	8.39E+09	1.07E+04	3.54E+08	4.46E+06	4.55E+03	2.64E+04	6.23E+07	1.00E+02	3.33E+02	5.83E+06
	Worst	1.00E+02	6.50E+09	1.13E+04	1.16E+10	1.22E+08	3.60E+09	8.07E+06	1.05E+04	3.05E+08	3.37E+08	1.71E+03	8.85E+03	1.62E+07
	Std	0.00E+00	1.01E+09	5.70E+03	1.56E+09	6.43E+07	1.57E+09	1.66E+06	3.05E+03	1.61E+08	1.44E+08	7.56E+02	4.29E+03	4.70E+06
	Median	1.00E+02	4.94E+09	1.59E+03	9.43E+09	6.15E+06	1.33E+09	5.99E+06	6.76E+03	1.54E+07	7.99E+07	5.26E+02	1.39E+03	1.15E+07
	Rank	1	12	4	13	8	11	6	5	9	10	2	3	7
C17-F3	Mean	3.00E+02	7.22E+03	3.02E+02	9.18E+03	1.35E+03	1.07E+04	1.66E+03	3.00E+02	2.93E+03	7.05E+02	9.76E+03	3.00E+02	1.40E+04
	Best	3.00E+02	3.92E+03	3.00E+02	4.96E+03	7.67E+02	4.07E+03	6.03E+02	3.00E+02	1.47E+03	4.63E+02	6.15E+03	3.00E+02	4.15E+03
	Worst	3.00E+02	9.67E+03	3.04E+02	1.23E+04	2.42E+03	1.51E+04	3.18E+03	3.00E+02	5.61E+03	8.63E+02	1.33E+04	3.00E+02	2.22E+04
	Std	0.00E+00	2.75E+03	2.27E+00	3.64E+03	8.32E+02	5.08E+03	1.32E+03	5.07E-02	2.08E+03	1.91E+02	3.19E+03	7.14E-14	1.03E+04
	Median	3.00E+02	7.64E+03	3.02E+02	9.74E+03	1.11E+03	1.18E+04	1.43E+03	3.00E+02	2.32E+03	7.47E+02	9.82E+03	3.00E+02	1.49E+04
	Rank	1	9	4	10	6	12	7	3	8	5	11	2	13
C17-F4	Mean	4.00E+02	8.93E+02	4.05E+02	1.30E+03	4.06E+02	5.68E+02	4.24E+02	4.03E+02	4.11E+02	4.09E+02	4.04E+02	4.19E+02	4.14E+02
	Best	4.00E+02	6.59E+02	4.01E+02	8.23E+02	4.02E+02	4.74E+02	4.06E+02	4.02E+02	4.06E+02	4.08E+02	4.03E+02	4.00E+02	4.11E+02
	Worst	4.00E+02	1.10E+03	4.06E+02	1.78E+03	4.11E+02	6.77E+02	4.70E+02	4.05E+02	4.27E+02	4.09E+02	4.06E+02	4.67E+02	4.18E+02
	Std	0.00E+00	2.18E+02	2.58E+00	4.42E+02	4.56E+00	1.08E+02	3.35E+01	1.78E+00	1.15E+01	5.68E-01	1.19E+00	3.49E+01	3.06E+00
	Median	4.00E+02	9.04E+02	4.05E+02	1.31E+03	4.06E+02	5.60E+02	4.10E+02	4.03E+02	4.06E+02	4.09E+02	4.04E+02	4.05E+02	4.14E+02
	Rank	1	12	4	13	5	11	10	2	7	6	3	9	8
C17-F5	Mean	5.01E+02	5.59E+02	5.42E+02	5.70E+02	5.12E+02	5.62E+02	5.39E+02	5.23E+02	5.13E+02	5.33E+02	5.52E+02	5.27E+02	5.27E+02
	Best	5.01E+02	5.45E+02	5.26E+02	5.56E+02	5.08E+02	5.42E+02	5.23E+02	5.10E+02	5.08E+02	5.27E+02	5.47E+02	5.11E+02	5.22E+02
	Worst	5.02E+02	5.69E+02	5.60E+02	5.84E+02	5.17E+02	5.93E+02	5.74E+02	5.37E+02	5.20E+02	5.36E+02	5.63E+02	5.50E+02	5.32E+02
	Std	5.41E-01	1.15E+01	1.97E+01	1.72E+01	5.29E+00	2.47E+01	2.62E+01	1.21E+01	5.31E+00	4.14E+00	8.29E+00	1.96E+01	4.93E+00
	Median	5.01E+02	5.61E+02	5.42E+02	5.70E+02	5.12E+02	5.57E+02	5.31E+02	5.22E+02	5.11E+02	5.34E+02	5.48E+02	5.23E+02	5.26E+02
	Rank	1	11	9	13	2	12	8	4	3	7	10	5	6
C17-F6	Mean	6.00E+02	6.31E+02	6.17E+02	6.39E+02	6.01E+02	6.24E+02	6.22E+02	6.02E+02	6.01E+02	6.07E+02	6.17E+02	6.07E+02	6.10E+02
	Best	6.00E+02	6.27E+02	6.16E+02	6.36E+02	6.01E+02	6.15E+02	6.07E+02	6.00E+02	6.01E+02	6.05E+02	6.03E+02	6.01E+02	6.07E+02
	Worst	6.00E+02	6.36E+02	6.19E+02	6.43E+02	6.02E+02	6.39E+02	6.44E+02	6.04E+02	6.02E+02	6.10E+02	6.35E+02	6.19E+02	6.14E+02
	Std	0.00E+00	4.19E+00	1.79E+00	3.52E+00	8.45E-01	1.15E+01	1.66E+01	1.81E+00	4.88E-01	2.58E+00	1.61E+01	8.52E+00	3.53E+00
	Median	6.00E+02	6.31E+02	6.16E+02	6.39E+02	6.01E+02	6.21E+02	6.19E+02	6.02E+02	6.01E+02	6.06E+02	6.14E+02	6.04E+02	6.09E+02
	Rank	1	12	9	13	3	11	10	4	2	5	8	6	7
C17-F7	Mean	7.11E+02	7.93E+02	7.64E+02	8.01E+02	7.24E+02	8.24E+02	7.60E+02	7.30E+02	7.25E+02	7.51E+02	7.17E+02	7.32E+02	7.36E+02
	Best	7.11E+02	7.77E+02	7.43E+02	7.88E+02	7.20E+02	7.86E+02	7.50E+02	7.17E+02	7.17E+02	7.46E+02	7.15E+02	7.25E+02	7.26E+02
	Worst	7.12E+02	8.04E+02	7.90E+02	8.13E+02	7.28E+02	8.64E+02	7.89E+02	7.49E+02	7.42E+02	7.58E+02	7.20E+02	7.43E+02	7.40E+02
	Std	5.57E-01	1.26E+01	2.39E+01	1.28E+01	3.80E+00	3.72E+01	2.07E+01	1.45E+01	1.26E+01	5.93E+00	2.72E+00	8.95E+00	7.34E+00
	Median	7.11E+02	7.96E+02	7.61E+02	8.01E+02	7.24E+02	8.24E+02	7.51E+02	7.27E+02	7.21E+02	7.49E+02	7.16E+02	7.30E+02	7.39E+02
	Rank	1	11	10	12	3	13	9	5	4	8	2	6	7
C17-F8	Mean	8.01E+02	8.46E+02	8.30E+02	8.52E+02	8.12E+02	8.47E+02	8.35E+02	8.11E+02	8.15E+02	8.36E+02	8.19E+02	8.22E+02	8.16E+02
	Best	8.01E+02	8.41E+02	8.20E+02	8.41E+02	8.09E+02	8.31E+02	8.18E+02	8.07E+02	8.10E+02	8.30E+02	8.12E+02	8.15E+02	8.12E+02
	Worst	8.02E+02	8.52E+02	8.45E+02	8.57E+02	8.14E+02	8.65E+02	8.47E+02	8.16E+02	8.20E+02	8.44E+02	8.27E+02	8.28E+02	8.24E+02
	Std	6.26E-01	6.36E+00	1.18E+01	7.96E+00	2.90E+00	1.66E+01	1.35E+01	3.97E+00	4.53E+00	8.00E+00	6.98E+00	7.06E+00	5.55E+00
	Median	8.01E+02	8.45E+02	8.28E+02	8.55E+02	8.13E+02	8.45E+02	8.38E+02	8.11E+02	8.16E+02	8.36E+02	8.19E+02	8.22E+02	8.14E+02
	Rank	1	11	8	13	3	12	9	2	4	10	6	7	5
C17-F9	Mean	9.00E+02	1.40E+03	1.18E+03	1.45E+03	9.05E+02	1.36E+03	1.36E+03	9.01E+02	9.12E+02	9.11E+02	9.00E+02	9.04E+02	9.05E+02
	Best	9.00E+02	1.26E+03	9.52E+02	1.35E+03	9.00E+02	1.16E+03	1.07E+03	9.00E+02	9.01E+02	9.07E+02	9.00E+02	9.01E+02	9.03E+02
	Worst	9.00E+02	1.54E+03	1.63E+03	1.58E+03	9.13E+02	1.64E+03	1.63E+03	9.03E+02	9.32E+02	9.19E+02	9.00E+02	9.12E+02	9.09E+02
	Std	0.00E+00	1.33E+02	3.44E+02	1.04E+02	6.15E+00	2.28E+02	2.57E+02	1.62E+00	1.60E+01	5.89E+00	0.00E+00	5.72E+00	2.98E+00
	Median	9.00E+02	1.41E+03	1.06E+03	1.43E+03	9.03E+02	1.33E+03	1.37E+03	9.00E+02	9.07E+02	9.10E+02	9.00E+02	9.02E+02	9.04E+02
	Rank	1	11	8	12	5	10	9	2	7	6	1	3	4
C17-F10	Mean	1.01E+03	2.25E+03	1.74E+03	2.51E+03	1.49E+03	1.99E+03	1.98E+03	1.75E+03	1.69E+03	2.12E+03	2.22E+03	1.90E+03	1.68E+03
	Best	1.00E+03	1.96E+03	1.46E+03	2.35E+03	1.37E+03	1.72E+03	1.43E+03	1.44E+03	1.52E+03	1.75E+03	1.95E+03	1.54E+03	1.40E+03
	Worst	1.01E+03	2.38E+03	2.35E+03	2.85E+03	1.57E+03	2.23E+03	2.48E+03	2.23E+03	1.95E+03	2.39E+03	2.32E+03	2.29E+03	2.06E+03
	Std	7.24E+00	2.14E+02	4.55E+02	2.57E+02	9.79E+01	2.89E+02	5.53E+02	4.16E+02	2.00E+02	3.00E+02	1.94E+02	3.38E+02	3.10E+02
	Median	1.01E+03	2.32E+03	1.58E+03	2.42E+03	1.52E+03	2.00E+03	2.00E+03	1.66E+03	1.65E+03	2.17E+03	2.30E+03	1.89E+03	1.64E+03
	Rank	1	12	5	13	2	9	8	6	4	10	11	7	3
C17-F11	Mean	1.10E+03	3.32E+03	1.15E+03	3.85E+03	1.13E+03	5.26E+03	1.15E+03	1.13E+03	1.15E+03	1.15E+03	1.14E+03	1.14E+03	2.32E+03
	Best	1.10E+03	2.13E+03	1.12E+03	1.44E+03	1.11E+03	5.12E+03	1.11E+03	1.11E+03	1.12E+03	1.14E+03	1.12E+03	1.13E+03	1.11E+03
	Worst	1.10E+03	4.48E+03	1.20E+03	6.23E+03	1.16E+03	5.34E+03	1.17E+03	1.15E+03	1.22E+03	1.17E+03	1.17E+03	1.16E+03	5.76E+03
	Std	0.00E+00	1.15E+03	3.87E+01	2.34E+03	2.24E+01	1.06E+02	2.88E+01	2.25E+01	5.17E+01	1.55E+01	2.17E+01	1.53E+01	2.49E+03
	Median	1.10E+03	3.34E+03	1.14E+03	3.87E+03	1.12E+03	5.29E+03	1.16E+03	1.13E+03	1.13E+03	1.14E+03	1.13E+03	1.14E+03	1.21E+03
	Rank	1	11	6	12	2	13	8	3	9	7	4	5	10
C17-F12	Mean	1.35E+03	3.38E+08	1.05E+06	6.75E+08	5.43E+05	9.95E+05	2.25E+06	9.85E+05	1.35E+06	4.83E+06	9.76E+05	7.80E+03	5.79E+05
	Best	1.32E+03	7.61E+07	3.41E+05	1.50E+08	1.91E+04	5.16E+05	1.64E+05	8.51E+03	4.35E+04	1.29E+06	4.54E+05	2.47E+03	1.68E+05
	Worst	1.44E+03	5.91E+08	1.91E+06	1.18E+09	8.50E+05	1.22E+06	3.74E+06	3.09E+06	2.12E+06	8.56E+06	1.65E+06	1.34E+04	1.02E+06
	Std	6.24E+01	2.83E+08	7.99E+05	5.67E+08	3.98E+05	3.62E+05	1.81E+06	1.55E+06	9.96E+05	4.19E+06	5.51E+05	5.41E+03	3.82E+05
	Median	1.33E+03	3.43E+08	9.81E+05	6.85E+08	6.52E+05	1.12E+06	2.55E+06	4.19E+05	1.63E+06	4.74E+06	9.00E+05	7.68E+03	5.63E+05
	Rank	1	12	8	13	3	7	10	6	9	11	5	2	4
C17-F13	Mean	1.31E+03	1.65E+07	1.76E+04	3.29E+07	5.26E+03	1.22E+04	7.31E+03	6.49E+03	9.91E+03	1.61E+04	9.69E+03	6.39E+03	5.22E+04
	Best	1.30E+03	1.37E+06	2.66E+03	2.73E+06	3.62E+03	7.32E+03	3.20E+03	1.38E+03	6.28E+03	1.52E+04	4.89E+03	2.33E+03	8.23E+03
	Worst	1.31E+03	5.46E+07	3.01E+04	1.09E+08	6.42E+03	1.94E+04	1.46E+04	1.19E+04	1.38E+04	1.82E+04	1.36E+04	1.60E+04	1.72E+05
	Std	2.47E+00	2.77E+07	1.54E+04	5.55E+07	1.45E+03	5.66E+03	5.64E+03	5.93E+03	3.36E+03	1.60E+03	4.02E+03	7.08E+03	8.72E+04
	Median	1.30E+03	4.91E+06	1.88E+04	9.82E+06	5.50E+03	1.11E+04	5.74E+03	6.35E+03	9.77E+03	1.54E+04	1.01E+04	3.59E+03	1.40E+04
	Rank	1	12	10	13	2	8	5	4	7	9	6	3	11
C17-F14	Mean	1.40E+03	3.70E+03	2.00E+03	5.18E+03	1.92E+03	3.30E+03	1.51E+03	1.56E+03	2.31E+03	1.58E+03	5.39E+03	2.93E+03	1.25E+04
	Best	1.40E+03	3.08E+03	1.67E+03	4.54E+03	1.43E+03	1.48E+03	1.48E+03	1.42E+03	1.46E+03	1.51E+03	4.47E+03	1.43E+03	3.63E+03
	Worst	1.40E+03	4.88E+03	2.77E+03	6.67E+03	2.84E+03	5.41E+03	1.55E+03	1.97E+03	4.81E+03	1.61E+03	7.29E+03	6.61E+03	2.48E+04
	Std	5.41E-01	8.97E+02	5.64E+02	1.09E+03	7.18E+02	2.27E+03	4.10E+01	2.93E+02	1.82E+03	5.22E+01	1.44E+03	2.70E+03	9.76E+03
	Median	1.40E+03	3.43E+03	1.77E+03	4.76E+03	1.70E+03	3.16E+03	1.51E+03	1.43E+03	1.48E+03	1.60E+03	4.90E+03	1.83E+03	1.07E+04
	Rank	1	10	6	11	5	9	2	3	7	4	12	8	13
C17-F15	Mean	1.50E+03	9.84E+03	5.14E+03	1.34E+04	3.87E+03	6.77E+03	6.02E+03	1.54E+03	5.63E+03	1.70E+03	2.29E+04	8.68E+03	4.42E+03
	Best	1.50E+03	3.17E+03	2.05E+03	2.68E+03	3.15E+03	2.28E+03	1.99E+03	1.52E+03	3.48E+03	1.58E+03	1.08E+04	2.81E+03	1.87E+03
	Worst	1.50E+03	1.67E+04	1.22E+04	2.91E+04	4.75E+03	1.21E+04	1.29E+04	1.55E+03	6.67E+03	1.79E+03	3.44E+04	1.42E+04	7.74E+03
	Std	2.56E-01	6.36E+03	5.13E+03	1.26E+04	7.22E+02	4.58E+03	5.19E+03	1.27E+01	1.59E+03	1.10E+02	1.23E+04	5.19E+03	3.17E+03
	Median	1.50E+03	9.72E+03	3.17E+03	1.08E+04	3.79E+03	6.36E+03	4.57E+03	1.54E+03	6.19E+03	1.72E+03	2.33E+04	8.84E+03	4.04E+03
	Rank	1	11	6	12	4	9	8	2	7	3	13	10	5
C17-F16	Mean	1.60E+03	1.99E+03	1.80E+03	2.00E+03	1.68E+03	2.03E+03	1.94E+03	1.81E+03	1.72E+03	1.67E+03	2.05E+03	1.91E+03	1.79E+03
	Best	1.60E+03	1.92E+03	1.64E+03	1.81E+03	1.64E+03	1.85E+03	1.76E+03	1.72E+03	1.62E+03	1.65E+03	1.93E+03	1.81E+03	1.71E+03
	Worst	1.60E+03	2.10E+03	1.91E+03	2.26E+03	1.71E+03	2.21E+03	2.06E+03	1.87E+03	1.82E+03	1.73E+03	2.24E+03	2.06E+03	1.82E+03
	Std	3.44E-01	8.71E+01	1.25E+02	2.07E+02	3.28E+01	1.75E+02	1.55E+02	6.67E+01	9.01E+01	3.90E+01	1.52E+02	1.26E+02	5.82E+01
	Median	1.60E+03	1.96E+03	1.82E+03	1.96E+03	1.69E+03	2.03E+03	1.96E+03	1.82E+03	1.73E+03	1.66E+03	2.02E+03	1.88E+03	1.82E+03
	Rank	1	10	6	11	3	12	9	7	4	2	13	8	5
C17-F17	Mean	1.70E+03	1.82E+03	1.75E+03	1.81E+03	1.73E+03	1.80E+03	1.84E+03	1.84E+03	1.77E+03	1.76E+03	1.84E+03	1.75E+03	1.75E+03
	Best	1.70E+03	1.80E+03	1.73E+03	1.80E+03	1.72E+03	1.78E+03	1.77E+03	1.78E+03	1.72E+03	1.75E+03	1.75E+03	1.74E+03	1.75E+03
	Worst	1.70E+03	1.82E+03	1.79E+03	1.82E+03	1.77E+03	1.81E+03	1.88E+03	1.94E+03	1.86E+03	1.77E+03	1.96E+03	1.76E+03	1.76E+03
	Std	1.69E-01	1.20E+01	3.07E+01	1.21E+01	2.72E+01	1.17E+01	5.24E+01	8.49E+01	7.20E+01	1.04E+01	1.20E+02	5.94E+00	2.62E+00
	Median	1.70E+03	1.82E+03	1.74E+03	1.82E+03	1.72E+03	1.80E+03	1.85E+03	1.82E+03	1.74E+03	1.76E+03	1.83E+03	1.75E+03	1.75E+03
	Rank	1	10	3	9	2	8	11	12	7	6	13	4	5
C17-F18	Mean	1.81E+03	2.73E+06	1.14E+04	5.44E+06	1.06E+04	1.16E+04	2.23E+04	2.01E+04	1.91E+04	2.83E+04	9.36E+03	2.10E+04	1.23E+04
	Best	1.80E+03	1.41E+05	4.71E+03	2.70E+05	4.05E+03	7.21E+03	6.24E+03	8.39E+03	6.12E+03	2.30E+04	6.19E+03	2.83E+03	3.36E+03
	Worst	1.82E+03	7.91E+06	1.50E+04	1.58E+07	1.59E+04	1.56E+04	3.51E+04	3.23E+04	3.22E+04	3.53E+04	1.14E+04	3.90E+04	1.77E+04
	Std	1.10E+01	3.92E+06	5.01E+03	7.83E+06	5.84E+03	3.81E+03	1.51E+04	1.22E+04	1.44E+04	6.17E+03	2.42E+03	2.03E+04	6.83E+03
	Median	1.80E+03	1.43E+06	1.30E+04	2.85E+06	1.13E+04	1.18E+04	2.40E+04	1.98E+04	1.90E+04	2.74E+04	9.92E+03	2.10E+04	1.41E+04
	Rank	1	12	4	13	3	5	10	8	7	11	2	9	6
C17-F19	Mean	1.90E+03	3.70E+05	6.49E+03	6.72E+05	5.43E+03	1.20E+05	3.33E+04	1.91E+03	5.23E+03	4.57E+03	3.87E+04	2.39E+04	5.99E+03
	Best	1.90E+03	2.45E+04	2.16E+03	4.38E+04	2.30E+03	1.95E+03	7.40E+03	1.91E+03	1.94E+03	2.04E+03	1.07E+04	2.59E+03	2.20E+03
	Worst	1.90E+03	7.80E+05	1.27E+04	1.44E+06	9.08E+03	2.40E+05	6.10E+04	1.92E+03	1.33E+04	1.20E+04	5.61E+04	7.35E+04	9.53E+03
	Std	8.10E-01	3.59E+05	5.59E+03	6.88E+05	3.76E+03	1.48E+05	2.39E+04	7.32E+00	5.90E+03	5.40E+03	2.21E+04	3.64E+04	3.29E+03
	Median	1.90E+03	3.38E+05	5.54E+03	6.01E+05	5.18E+03	1.19E+05	3.25E+04	1.91E+03	2.85E+03	2.12E+03	4.40E+04	9.77E+03	6.12E+03
	Rank	1	12	7	13	5	11	9	2	4	3	10	8	6
C17-F20	Mean	2.00E+03	2.21E+03	2.16E+03	2.21E+03	2.09E+03	2.20E+03	2.20E+03	2.13E+03	2.16E+03	2.07E+03	2.24E+03	2.16E+03	2.05E+03
	Best	2.00E+03	2.16E+03	2.03E+03	2.16E+03	2.07E+03	2.10E+03	2.09E+03	2.04E+03	2.13E+03	2.06E+03	2.18E+03	2.14E+03	2.03E+03
	Worst	2.00E+03	2.27E+03	2.28E+03	2.27E+03	2.12E+03	2.31E+03	2.28E+03	2.24E+03	2.24E+03	2.08E+03	2.33E+03	2.19E+03	2.06E+03
	Std	0.00E+00	5.10E+01	1.23E+02	5.83E+01	2.23E+01	9.43E+01	9.42E+01	8.56E+01	5.39E+01	9.35E+00	8.04E+01	2.89E+01	1.06E+01
	Median	2.00E+03	2.20E+03	2.17E+03	2.21E+03	2.08E+03	2.19E+03	2.21E+03	2.13E+03	2.14E+03	2.07E+03	2.23E+03	2.16E+03	2.05E+03
	Rank	1	11	8	12	4	10	9	5	7	3	13	6	2
C17-F21	Mean	2.20E+03	2.29E+03	2.21E+03	2.26E+03	2.25E+03	2.32E+03	2.31E+03	2.25E+03	2.31E+03	2.30E+03	2.36E+03	2.31E+03	2.29E+03
	Best	2.20E+03	2.24E+03	2.20E+03	2.22E+03	2.25E+03	2.22E+03	2.22E+03	2.20E+03	2.30E+03	2.20E+03	2.34E+03	2.31E+03	2.23E+03
	Worst	2.20E+03	2.32E+03	2.24E+03	2.29E+03	2.26E+03	2.36E+03	2.35E+03	2.30E+03	2.31E+03	2.33E+03	2.38E+03	2.32E+03	2.33E+03
	Std	0.00E+00	3.55E+01	1.75E+01	3.12E+01	2.21E+00	7.33E+01	6.42E+01	6.38E+01	3.93E+00	6.70E+01	1.51E+01	7.99E+00	5.03E+01
	Median	2.20E+03	2.30E+03	2.21E+03	2.27E+03	2.25E+03	2.35E+03	2.33E+03	2.25E+03	2.31E+03	2.32E+03	2.36E+03	2.31E+03	2.31E+03
	Rank	1	6	2	5	4	12	9	3	10	8	13	11	7
C17-F22	Mean	2.30E+03	2.68E+03	2.31E+03	2.89E+03	2.30E+03	2.70E+03	2.32E+03	2.29E+03	2.31E+03	2.32E+03	2.30E+03	2.31E+03	2.32E+03
	Best	2.30E+03	2.57E+03	2.30E+03	2.69E+03	2.30E+03	2.44E+03	2.32E+03	2.23E+03	2.30E+03	2.31E+03	2.30E+03	2.30E+03	2.31E+03
	Worst	2.30E+03	2.79E+03	2.31E+03	3.04E+03	2.31E+03	2.89E+03	2.33E+03	2.31E+03	2.32E+03	2.33E+03	2.30E+03	2.34E+03	2.32E+03
	Std	1.58E-01	1.09E+02	3.25E+00	1.59E+02	3.69E+00	2.20E+02	5.72E+00	3.91E+01	1.01E+01	8.57E+00	1.72E-02	2.24E+01	3.28E+00
	Median	2.30E+03	2.68E+03	2.31E+03	2.92E+03	2.30E+03	2.72E+03	2.32E+03	2.30E+03	2.31E+03	2.32E+03	2.30E+03	2.30E+03	2.32E+03
	Rank	3	11	6	13	4	12	10	1	5	9	2	7	8
C17-F23	Mean	2.60E+03	2.69E+03	2.64E+03	2.70E+03	2.61E+03	2.72E+03	2.65E+03	2.62E+03	2.61E+03	2.64E+03	2.78E+03	2.64E+03	2.65E+03
	Best	2.60E+03	2.65E+03	2.63E+03	2.67E+03	2.61E+03	2.63E+03	2.63E+03	2.61E+03	2.61E+03	2.63E+03	2.72E+03	2.64E+03	2.63E+03
	Worst	2.60E+03	2.70E+03	2.66E+03	2.74E+03	2.62E+03	2.76E+03	2.67E+03	2.63E+03	2.62E+03	2.65E+03	2.92E+03	2.65E+03	2.66E+03
	Std	1.44E+00	2.69E+01	1.44E+01	3.39E+01	2.50E+00	6.29E+01	2.15E+01	1.12E+01	6.77E+00	9.38E+00	9.97E+01	8.99E+00	1.41E+01
	Median	2.60E+03	2.69E+03	2.64E+03	2.69E+03	2.61E+03	2.74E+03	2.65E+03	2.62E+03	2.61E+03	2.64E+03	2.75E+03	2.64E+03	2.66E+03
	Rank	1	10	5	11	3	12	8	4	2	6	13	7	9
C17-F24	Mean	2.63E+03	2.78E+03	2.76E+03	2.84E+03	2.63E+03	2.67E+03	2.76E+03	2.68E+03	2.74E+03	2.75E+03	2.74E+03	2.76E+03	2.72E+03
	Best	2.52E+03	2.73E+03	2.73E+03	2.82E+03	2.61E+03	2.53E+03	2.73E+03	2.50E+03	2.72E+03	2.73E+03	2.50E+03	2.75E+03	2.55E+03
	Worst	2.73E+03	2.85E+03	2.78E+03	2.90E+03	2.64E+03	2.81E+03	2.79E+03	2.76E+03	2.76E+03	2.77E+03	2.89E+03	2.78E+03	2.81E+03
	Std	1.27E+02	5.74E+01	2.78E+01	4.60E+01	1.44E+01	1.60E+02	2.89E+01	1.31E+02	2.12E+01	1.64E+01	1.81E+02	1.77E+01	1.28E+02
	Median	2.64E+03	2.77E+03	2.77E+03	2.82E+03	2.63E+03	2.66E+03	2.75E+03	2.73E+03	2.75E+03	2.75E+03	2.79E+03	2.75E+03	2.76E+03
	Rank	1	12	11	13	2	3	9	4	7	8	6	10	5
C17-F25	Mean	2.93E+03	3.13E+03	2.91E+03	3.26E+03	2.92E+03	3.13E+03	2.91E+03	2.92E+03	2.94E+03	2.93E+03	2.92E+03	2.92E+03	2.95E+03
	Best	2.90E+03	3.06E+03	2.90E+03	3.20E+03	2.92E+03	2.91E+03	2.77E+03	2.90E+03	2.92E+03	2.92E+03	2.90E+03	2.90E+03	2.94E+03
	Worst	2.95E+03	3.28E+03	2.95E+03	3.33E+03	2.92E+03	3.63E+03	2.96E+03	2.94E+03	2.95E+03	2.95E+03	2.94E+03	2.95E+03	2.96E+03
	Std	2.51E+01	1.11E+02	2.52E+01	6.14E+01	4.63E+00	3.67E+02	9.89E+01	2.47E+01	1.18E+01	2.15E+01	2.30E+01	2.80E+01	1.19E+01
	Median	2.94E+03	3.09E+03	2.90E+03	3.26E+03	2.92E+03	2.98E+03	2.95E+03	2.92E+03	2.94E+03	2.93E+03	2.92E+03	2.92E+03	2.95E+03
	Rank	7	12	2	13	3	11	1	4	9	8	5	6	10
C17-F26	Mean	2.90E+03	3.53E+03	2.98E+03	3.72E+03	3.01E+03	3.59E+03	3.17E+03	2.90E+03	3.25E+03	3.19E+03	3.82E+03	2.90E+03	2.90E+03
	Best	2.90E+03	3.22E+03	2.81E+03	3.41E+03	2.89E+03	3.13E+03	2.93E+03	2.90E+03	2.97E+03	2.91E+03	2.81E+03	2.81E+03	2.72E+03
	Worst	2.90E+03	3.74E+03	3.15E+03	4.04E+03	3.28E+03	4.21E+03	3.56E+03	2.90E+03	3.86E+03	3.83E+03	4.29E+03	3.00E+03	3.10E+03
	Std	4.04E-13	2.51E+02	2.08E+02	2.97E+02	1.97E+02	5.74E+02	3.04E+02	3.73E-02	4.50E+02	4.68E+02	7.45E+02	8.62E+01	2.12E+02
	Median	2.90E+03	3.58E+03	2.97E+03	3.71E+03	2.93E+03	3.51E+03	3.10E+03	2.90E+03	3.08E+03	3.01E+03	4.09E+03	2.90E+03	2.89E+03
	Rank	2	10	5	12	6	11	7	3	9	8	13	4	1
C17-F27	Mean	3.09E+03	3.20E+03	3.12E+03	3.23E+03	3.10E+03	3.18E+03	3.19E+03	3.09E+03	3.11E+03	3.11E+03	3.22E+03	3.13E+03	3.16E+03
	Best	3.09E+03	3.16E+03	3.10E+03	3.13E+03	3.09E+03	3.10E+03	3.18E+03	3.09E+03	3.09E+03	3.10E+03	3.21E+03	3.10E+03	3.12E+03
	Worst	3.09E+03	3.28E+03	3.18E+03	3.41E+03	3.13E+03	3.22E+03	3.20E+03	3.09E+03	3.17E+03	3.17E+03	3.24E+03	3.18E+03	3.21E+03
	Std	2.86E-13	5.86E+01	4.25E+01	1.37E+02	2.04E+01	5.64E+01	1.20E+01	2.58E+00	4.22E+01	3.91E+01	1.56E+01	3.78E+01	4.39E+01
	Median	3.09E+03	3.19E+03	3.10E+03	3.18E+03	3.10E+03	3.19E+03	3.19E+03	3.09E+03	3.10E+03	3.10E+03	3.22E+03	3.13E+03	3.15E+03
	Rank	1	11	6	13	3	9	10	2	5	4	12	7	8
C17-F28	Mean	3.10E+03	3.57E+03	3.23E+03	3.75E+03	3.21E+03	3.57E+03	3.28E+03	3.23E+03	3.33E+03	3.32E+03	3.44E+03	3.30E+03	3.24E+03
	Best	3.10E+03	3.53E+03	3.10E+03	3.67E+03	3.16E+03	3.40E+03	3.15E+03	3.10E+03	3.19E+03	3.21E+03	3.42E+03	3.17E+03	3.14E+03
	Worst	3.10E+03	3.60E+03	3.38E+03	3.81E+03	3.24E+03	3.77E+03	3.38E+03	3.38E+03	3.40E+03	3.38E+03	3.45E+03	3.38E+03	3.50E+03
	Std	0.00E+00	3.59E+01	1.34E+02	6.85E+01	3.69E+01	2.07E+02	1.27E+02	1.67E+02	1.05E+02	8.78E+01	1.53E+01	1.01E+02	1.86E+02
	Median	3.10E+03	3.58E+03	3.22E+03	3.76E+03	3.23E+03	3.55E+03	3.29E+03	3.23E+03	3.37E+03	3.34E+03	3.43E+03	3.32E+03	3.16E+03
	Rank	1	12	3	13	2	11	6	4	9	8	10	7	5
C17-F29	Mean	3.13E+03	3.33E+03	3.28E+03	3.37E+03	3.20E+03	3.23E+03	3.34E+03	3.20E+03	3.26E+03	3.21E+03	3.34E+03	3.26E+03	3.23E+03
	Best	3.13E+03	3.31E+03	3.21E+03	3.30E+03	3.16E+03	3.16E+03	3.23E+03	3.14E+03	3.19E+03	3.16E+03	3.23E+03	3.17E+03	3.19E+03
	Worst	3.13E+03	3.35E+03	3.36E+03	3.43E+03	3.24E+03	3.30E+03	3.48E+03	3.28E+03	3.37E+03	3.23E+03	3.61E+03	3.34E+03	3.28E+03
	Std	2.70E+00	1.62E+01	8.33E+01	7.45E+01	3.61E+01	5.98E+01	1.14E+02	6.35E+01	9.40E+01	3.42E+01	2.02E+02	8.57E+01	4.29E+01
	Median	3.13E+03	3.33E+03	3.27E+03	3.37E+03	3.20E+03	3.23E+03	3.32E+03	3.19E+03	3.24E+03	3.22E+03	3.25E+03	3.27E+03	3.23E+03
	Rank	1	10	9	13	3	5	12	2	7	4	11	8	6
C17-F30	Mean	3.42E+03	2.15E+06	2.81E+05	3.51E+06	3.96E+05	5.86E+05	9.47E+05	2.89E+05	8.93E+05	5.80E+04	7.47E+05	3.70E+05	1.46E+06
	Best	3.39E+03	1.59E+06	1.00E+05	7.90E+05	1.54E+04	1.07E+05	4.42E+03	7.25E+03	3.22E+04	2.81E+04	5.74E+05	6.26E+03	5.02E+05
	Worst	3.44E+03	3.08E+06	7.33E+05	5.54E+06	5.84E+05	1.24E+06	3.57E+06	1.10E+06	1.29E+06	9.72E+04	9.54E+05	7.33E+05	3.32E+06
	Std	3.02E+01	7.02E+05	3.28E+05	2.16E+06	2.81E+05	5.24E+05	1.91E+06	5.90E+05	6.44E+05	3.67E+04	1.72E+05	4.56E+05	1.45E+06
	Median	3.42E+03	1.98E+06	1.46E+05	3.85E+06	4.92E+05	4.99E+05	1.04E+05	2.37E+04	1.12E+06	5.33E+04	7.30E+05	3.70E+05	1.00E+06
	Rank	1	12	3	13	6	7	10	4	9	2	8	5	11
Sum rank		38	318	177	350	106	286	239	116	188	191	238	183	197
Mean rank		1.31E+00	1.10E+01	6.10E+00	1.21E+01	3.66E+00	9.86E+00	8.24E+00	4.00E+00	6.48E+00	6.59E+00	8.21E+00	6.31E+00	6.79E+00
Total rank		1	12	4	13	2	11	10	3	6	7	9	5	8

**Table 3:** Optimization results of CEC 2017 test suite (dimension = 30).

		LOA	WSO	AVOA	RSA	MPA	TSA	WOA	MVO	GWO	TLBO	GSA	PSO	GA
C17-F1	Mean	1.00E+02	2.49E+10	2.96E+03	3.90E+10	2.54E+04	1.70E+10	1.61E+09	5.10E+05	1.58E+09	5.86E+09	9.97E+06	1.33E+09	1.69E+08
	Best	1.00E+02	2.15E+10	2.71E+02	3.48E+10	1.17E+04	1.07E+10	1.27E+09	3.97E+05	2.61E+08	3.70E+09	2.41E+03	3.56E+03	1.26E+08
	Worst	1.00E+02	3.12E+10	7.27E+03	4.80E+10	3.87E+04	2.32E+10	2.00E+09	6.49E+05	4.77E+09	8.73E+09	3.48E+07	5.33E+09	2.33E+08
	Std	8.93E-15	4.94E+09	3.57E+03	6.62E+09	1.42E+04	6.36E+09	4.07E+08	1.36E+05	2.32E+09	2.29E+09	1.82E+07	2.90E+09	5.04E+07
	Median	1.00E+02	2.35E+10	2.15E+03	3.67E+10	2.57E+04	1.71E+10	1.59E+09	4.98E+05	6.53E+08	5.50E+09	2.54E+06	3.03E+06	1.59E+08
	Rank	1	12	2	13	3	11	9	4	8	10	5	7	6
C17-F3	Mean	3.00E+02	9.24E+04	4.24E+04	6.99E+04	1.06E+03	4.48E+04	2.20E+05	1.70E+03	3.96E+04	3.29E+04	9.10E+04	3.03E+04	1.59E+05
	Best	3.00E+02	8.44E+04	2.31E+04	5.41E+04	8.23E+02	4.25E+04	1.82E+05	1.34E+03	3.46E+04	2.80E+04	7.84E+04	2.16E+04	1.20E+05
	Worst	3.00E+02	1.01E+05	5.49E+04	7.59E+04	1.30E+03	4.72E+04	2.53E+05	2.33E+03	4.42E+04	3.57E+04	1.00E+05	3.89E+04	2.21E+05
	Std	0.00E+00	9.17E+03	1.49E+04	1.15E+04	2.35E+02	2.60E+03	3.20E+04	4.78E+02	4.29E+03	3.74E+03	1.07E+04	8.57E+03	5.19E+04
	Median	3.00E+02	9.19E+04	4.59E+04	7.48E+04	1.06E+03	4.48E+04	2.23E+05	1.57E+03	3.98E+04	3.40E+04	9.27E+04	3.03E+04	1.47E+05
	Rank	1	11	7	9	2	8	13	3	6	5	10	4	12
C17-F4	Mean	4.59E+02	6.14E+03	5.12E+02	9.35E+03	4.91E+02	4.34E+03	8.37E+02	4.95E+02	5.66E+02	8.85E+02	5.88E+02	6.16E+02	7.94E+02
	Best	4.59E+02	3.46E+03	4.90E+02	6.00E+03	4.81E+02	1.02E+03	7.75E+02	4.87E+02	5.13E+02	6.89E+02	5.69E+02	5.13E+02	7.45E+02
	Worst	4.59E+02	8.31E+03	5.29E+02	1.31E+04	5.12E+02	7.20E+03	9.14E+02	5.08E+02	5.96E+02	1.27E+03	6.10E+02	7.95E+02	8.17E+02
	Std	0.00E+00	2.19E+03	1.76E+01	3.19E+03	1.53E+01	2.84E+03	6.90E+01	9.75E+00	3.95E+01	2.81E+02	1.97E+01	1.41E+02	3.68E+01
	Median	4.59E+02	6.40E+03	5.14E+02	9.17E+03	4.86E+02	4.57E+03	8.29E+02	4.92E+02	5.78E+02	7.94E+02	5.86E+02	5.78E+02	8.08E+02
	Rank	1	12	4	13	2	11	9	3	5	10	6	7	8
C17-F5	Mean	5.02E+02	8.28E+02	7.15E+02	8.66E+02	5.79E+02	7.80E+02	8.08E+02	6.14E+02	6.16E+02	7.57E+02	7.12E+02	6.26E+02	6.93E+02
	Best	5.01E+02	8.09E+02	6.80E+02	8.41E+02	5.58E+02	7.53E+02	7.80E+02	6.00E+02	5.78E+02	7.36E+02	6.94E+02	6.03E+02	6.46E+02
	Worst	5.04E+02	8.49E+02	7.70E+02	8.98E+02	6.01E+02	8.12E+02	8.21E+02	6.47E+02	6.44E+02	7.82E+02	7.37E+02	6.73E+02	7.53E+02
	Std	1.40E+00	1.78E+01	4.48E+01	2.98E+01	1.98E+01	3.04E+01	2.03E+01	2.44E+01	3.55E+01	2.45E+01	2.11E+01	3.48E+01	4.81E+01
	Median	5.02E+02	8.28E+02	7.04E+02	8.62E+02	5.79E+02	7.77E+02	8.15E+02	6.04E+02	6.22E+02	7.56E+02	7.09E+02	6.14E+02	6.86E+02
	Rank	1	12	8	13	2	10	11	3	4	9	7	5	6
C17-F6	Mean	6.00E+02	6.76E+02	6.44E+02	6.79E+02	6.03E+02	6.73E+02	6.72E+02	6.23E+02	6.11E+02	6.41E+02	6.54E+02	6.44E+02	6.29E+02
	Best	6.00E+02	6.74E+02	6.42E+02	6.74E+02	6.02E+02	6.58E+02	6.62E+02	6.12E+02	6.04E+02	6.34E+02	6.53E+02	6.33E+02	6.22E+02
	Worst	6.00E+02	6.77E+02	6.47E+02	6.85E+02	6.04E+02	6.82E+02	6.77E+02	6.35E+02	6.18E+02	6.52E+02	6.54E+02	6.55E+02	6.33E+02
	Std	7.14E-14	1.12E+00	2.27E+00	5.71E+00	1.20E+00	1.18E+01	7.69E+00	1.19E+01	6.07E+00	8.49E+00	7.90E-01	1.05E+01	5.23E+00
	Median	6.00E+02	6.76E+02	6.44E+02	6.78E+02	6.03E+02	6.76E+02	6.75E+02	6.23E+02	6.11E+02	6.39E+02	6.53E+02	6.45E+02	6.30E+02
	Rank	1	12	7	13	2	11	10	4	3	6	9	8	5
C17-F7	Mean	7.33E+02	1.27E+03	1.13E+03	1.31E+03	8.41E+02	1.20E+03	1.28E+03	8.48E+02	8.78E+02	1.06E+03	9.59E+02	8.71E+02	9.56E+02
	Best	7.33E+02	1.22E+03	1.02E+03	1.30E+03	8.16E+02	1.06E+03	1.24E+03	7.98E+02	8.12E+02	9.75E+02	9.15E+02	8.51E+02	9.18E+02
	Worst	7.35E+02	1.30E+03	1.28E+03	1.33E+03	8.93E+02	1.34E+03	1.35E+03	9.19E+02	9.17E+02	1.13E+03	1.03E+03	8.97E+02	1.01E+03
	Std	8.21E-01	3.78E+01	1.27E+02	1.70E+01	3.79E+01	1.33E+02	5.97E+01	5.64E+01	5.01E+01	8.91E+01	5.34E+01	2.18E+01	4.09E+01
	Median	7.33E+02	1.27E+03	1.10E+03	1.30E+03	8.29E+02	1.20E+03	1.26E+03	8.39E+02	8.93E+02	1.06E+03	9.48E+02	8.69E+02	9.49E+02
	Rank	1	11	9	13	2	10	12	3	5	8	7	4	6
C17-F8	Mean	8.03E+02	1.07E+03	9.43E+02	1.11E+03	8.87E+02	1.05E+03	1.02E+03	8.89E+02	8.88E+02	1.01E+03	9.54E+02	9.18E+02	9.77E+02
	Best	8.01E+02	1.06E+03	9.14E+02	1.09E+03	8.80E+02	1.00E+03	9.65E+02	8.60E+02	8.81E+02	9.94E+02	9.31E+02	9.07E+02	9.62E+02
	Worst	8.04E+02	1.09E+03	9.63E+02	1.13E+03	8.94E+02	1.15E+03	1.06E+03	9.18E+02	8.96E+02	1.04E+03	9.79E+02	9.33E+02	9.97E+02
	Std	1.55E+00	1.69E+01	2.44E+01	2.51E+01	6.38E+00	7.25E+01	4.35E+01	2.74E+01	6.77E+00	2.36E+01	2.34E+01	1.28E+01	1.92E+01
	Median	8.04E+02	1.07E+03	9.47E+02	1.10E+03	8.86E+02	1.02E+03	1.03E+03	8.90E+02	8.87E+02	1.01E+03	9.53E+02	9.16E+02	9.75E+02
	Rank	1	12	6	13	2	11	10	4	3	9	7	5	8
C17-F9	Mean	9.00E+02	1.05E+04	4.63E+03	1.01E+04	1.08E+03	1.10E+04	1.05E+04	5.22E+03	2.02E+03	5.53E+03	3.92E+03	3.41E+03	1.27E+03
	Best	9.00E+02	8.94E+03	3.43E+03	9.89E+03	9.28E+02	6.69E+03	8.05E+03	4.17E+03	1.51E+03	4.00E+03	3.41E+03	2.06E+03	1.07E+03
	Worst	9.00E+02	1.19E+04	5.26E+03	1.03E+04	1.22E+03	1.48E+04	1.25E+04	7.97E+03	2.77E+03	8.32E+03	4.71E+03	5.18E+03	1.47E+03
	Std	7.14E-14	1.33E+03	8.94E+02	1.84E+02	1.47E+02	3.64E+03	2.45E+03	1.99E+03	6.65E+02	2.13E+03	6.22E+02	1.44E+03	2.05E+02
	Median	9.00E+02	1.05E+04	4.90E+03	1.02E+04	1.08E+03	1.12E+04	1.07E+04	4.38E+03	1.90E+03	4.89E+03	3.78E+03	3.21E+03	1.27E+03
	Rank	1	11	7	10	2	13	12	8	4	9	6	5	3
C17-F10	Mean	2.29E+03	6.98E+03	5.30E+03	7.63E+03	3.91E+03	6.36E+03	6.29E+03	4.54E+03	4.67E+03	7.65E+03	4.73E+03	4.91E+03	5.96E+03
	Best	1.85E+03	6.41E+03	4.61E+03	6.80E+03	3.57E+03	5.01E+03	5.45E+03	4.27E+03	4.18E+03	7.31E+03	4.48E+03	4.68E+03	5.50E+03
	Worst	2.53E+03	7.29E+03	5.76E+03	8.24E+03	4.32E+03	6.93E+03	7.54E+03	4.91E+03	4.96E+03	7.83E+03	5.12E+03	5.36E+03	6.48E+03
	Std	3.27E+02	4.27E+02	6.04E+02	6.61E+02	3.74E+02	9.84E+02	1.01E+03	3.48E+02	3.70E+02	2.54E+02	3.31E+02	3.33E+02	5.02E+02
	Median	2.40E+03	7.12E+03	5.42E+03	7.75E+03	3.87E+03	6.74E+03	6.09E+03	4.48E+03	4.76E+03	7.74E+03	4.65E+03	4.80E+03	5.93E+03
	Rank	1	11	7	12	2	10	9	3	4	13	5	6	8
C17-F11	Mean	1.10E+03	7.19E+03	1.25E+03	8.43E+03	1.17E+03	4.94E+03	7.49E+03	1.30E+03	2.14E+03	1.95E+03	2.81E+03	1.24E+03	8.78E+03
	Best	1.10E+03	5.93E+03	1.19E+03	6.87E+03	1.12E+03	3.52E+03	5.40E+03	1.26E+03	1.38E+03	1.57E+03	2.19E+03	1.21E+03	3.25E+03
	Worst	1.11E+03	8.23E+03	1.31E+03	9.48E+03	1.20E+03	7.43E+03	1.11E+04	1.34E+03	4.18E+03	2.65E+03	3.45E+03	1.27E+03	1.64E+04
	Std	2.34E+00	1.10E+03	5.67E+01	1.30E+03	3.64E+01	1.91E+03	2.69E+03	4.98E+01	1.48E+03	5.20E+02	6.48E+02	2.89E+01	6.15E+03
	Median	1.10E+03	7.31E+03	1.25E+03	8.68E+03	1.17E+03	4.40E+03	6.75E+03	1.30E+03	1.51E+03	1.78E+03	2.80E+03	1.24E+03	7.71E+03
	Rank	1	10	4	12	2	9	11	5	7	6	8	3	13
C17-F12	Mean	1.74E+03	6.69E+09	1.99E+07	1.04E+10	2.07E+04	4.83E+09	2.36E+08	1.07E+07	5.00E+07	2.88E+08	1.90E+08	2.44E+06	7.32E+06
	Best	1.72E+03	5.53E+09	2.80E+06	9.26E+09	1.48E+04	2.49E+09	6.03E+07	4.97E+06	4.86E+06	1.84E+08	3.67E+07	2.64E+05	5.07E+06
	Worst	1.76E+03	8.50E+09	4.85E+07	1.31E+10	2.64E+04	6.31E+09	4.71E+08	2.59E+07	1.05E+08	5.00E+08	6.06E+08	4.85E+06	9.58E+06
	Std	2.19E+01	1.38E+09	2.19E+07	1.97E+09	5.37E+03	1.80E+09	2.06E+08	1.10E+07	4.75E+07	1.56E+08	3.02E+08	2.15E+06	2.23E+06
	Median	1.75E+03	6.37E+09	1.41E+07	9.61E+09	2.08E+04	5.26E+09	2.06E+08	5.97E+06	4.52E+07	2.34E+08	5.81E+07	2.32E+06	7.32E+06
	Rank	1	12	6	13	2	11	9	5	7	10	8	3	4
C17-F13	Mean	1.32E+03	5.44E+09	1.43E+05	1.00E+10	1.86E+03	1.39E+09	8.61E+05	8.67E+04	7.19E+05	8.40E+07	3.48E+04	3.09E+04	1.13E+07
	Best	1.31E+03	2.65E+09	7.89E+04	5.27E+09	1.60E+03	1.88E+07	4.06E+05	3.47E+04	8.69E+04	5.83E+07	2.82E+04	1.28E+04	3.08E+06
	Worst	1.32E+03	7.62E+09	2.25E+05	1.23E+10	2.37E+03	4.84E+09	1.27E+06	1.74E+05	2.23E+06	1.24E+08	5.09E+04	6.97E+04	2.44E+07
	Std	2.11E+00	2.24E+09	6.62E+04	3.51E+09	3.81E+02	2.52E+09	4.92E+05	7.12E+04	1.11E+06	3.08E+07	1.18E+04	2.85E+04	9.95E+06
	Median	1.31E+03	5.75E+09	1.33E+05	1.13E+10	1.74E+03	3.59E+08	8.83E+05	6.90E+04	2.79E+05	7.69E+07	3.00E+04	2.05E+04	8.95E+06
	Rank	1	12	6	13	2	11	8	5	7	10	4	3	9
C17-F14	Mean	1.42E+03	1.80E+06	2.58E+05	2.09E+06	1.44E+03	1.12E+06	2.11E+06	1.94E+04	5.07E+05	1.33E+05	1.09E+06	1.79E+04	1.91E+06
	Best	1.42E+03	1.11E+06	3.61E+04	1.05E+06	1.44E+03	7.99E+05	3.42E+04	4.82E+03	3.27E+04	7.74E+04	7.06E+05	3.09E+03	3.16E+05
	Worst	1.42E+03	2.28E+06	5.97E+05	3.11E+06	1.44E+03	1.58E+06	6.46E+06	3.30E+04	1.09E+06	1.53E+05	1.64E+06	3.27E+04	3.22E+06
	Std	8.80E-01	5.96E+05	2.69E+05	1.08E+06	3.87E+00	3.89E+05	3.21E+06	1.32E+04	5.82E+05	4.04E+04	4.80E+05	1.41E+04	1.46E+06
	Median	1.42E+03	1.91E+06	1.99E+05	2.10E+06	1.44E+03	1.05E+06	9.82E+05	1.99E+04	4.54E+05	1.51E+05	1.00E+06	1.80E+04	2.05E+06
	Rank	1	10	6	12	2	9	13	4	7	5	8	3	11
C17-F15	Mean	1.50E+03	2.89E+08	3.57E+04	5.68E+08	1.61E+03	1.37E+07	4.79E+06	4.07E+04	1.50E+07	4.88E+06	1.53E+04	4.62E+03	9.08E+05
	Best	1.50E+03	2.50E+08	1.05E+04	4.90E+08	1.58E+03	5.38E+06	2.21E+05	2.36E+04	9.35E+04	1.11E+06	1.09E+04	1.89E+03	1.67E+05
	Worst	1.50E+03	3.20E+08	5.79E+04	6.27E+08	1.63E+03	3.18E+07	1.56E+07	6.73E+04	5.63E+07	9.18E+06	2.08E+04	8.52E+03	2.04E+06
	Std	9.31E-01	3.78E+07	2.18E+04	7.31E+07	2.61E+01	1.33E+07	7.92E+06	2.06E+04	3.00E+07	3.60E+06	4.49E+03	3.19E+03	9.30E+05
	Median	1.50E+03	2.93E+08	3.72E+04	5.78E+08	1.62E+03	8.74E+06	1.69E+06	3.60E+04	1.88E+06	4.61E+06	1.48E+04	4.03E+03	7.16E+05
	Rank	1	12	5	13	2	10	8	6	11	9	4	3	7
C17-F16	Mean	1.66E+03	4.19E+03	2.94E+03	4.81E+03	2.01E+03	3.19E+03	4.12E+03	2.54E+03	2.50E+03	3.38E+03	3.57E+03	2.87E+03	2.89E+03
	Best	1.61E+03	3.87E+03	2.51E+03	4.07E+03	1.73E+03	2.79E+03	3.40E+03	2.32E+03	2.36E+03	3.19E+03	3.39E+03	2.64E+03	2.56E+03
	Worst	1.74E+03	4.45E+03	3.43E+03	5.48E+03	2.25E+03	3.44E+03	4.93E+03	2.79E+03	2.62E+03	3.60E+03	3.73E+03	3.13E+03	3.22E+03
	Std	6.74E+01	2.89E+02	4.13E+02	8.20E+02	2.56E+02	3.12E+02	6.86E+02	2.23E+02	1.44E+02	1.95E+02	1.67E+02	2.75E+02	3.50E+02
	Median	1.65E+03	4.21E+03	2.90E+03	4.85E+03	2.03E+03	3.27E+03	4.07E+03	2.53E+03	2.52E+03	3.36E+03	3.57E+03	2.85E+03	2.89E+03
	Rank	1	12	7	13	2	8	11	4	3	9	10	5	6
C17-F17	Mean	1.73E+03	3.33E+03	2.44E+03	3.62E+03	1.86E+03	3.20E+03	2.80E+03	2.07E+03	1.93E+03	2.17E+03	2.49E+03	2.31E+03	2.14E+03
	Best	1.72E+03	2.76E+03	2.30E+03	3.26E+03	1.75E+03	2.20E+03	2.34E+03	2.02E+03	1.80E+03	1.96E+03	2.39E+03	2.08E+03	2.09E+03
	Worst	1.73E+03	4.04E+03	2.55E+03	4.26E+03	1.92E+03	5.82E+03	3.11E+03	2.21E+03	2.07E+03	2.46E+03	2.63E+03	2.69E+03	2.21E+03
	Std	7.30E+00	5.95E+02	1.19E+02	4.96E+02	7.92E+01	1.91E+03	3.57E+02	1.04E+02	1.38E+02	2.31E+02	1.28E+02	2.94E+02	5.59E+01
	Median	1.73E+03	3.26E+03	2.46E+03	3.48E+03	1.88E+03	2.39E+03	2.87E+03	2.02E+03	1.92E+03	2.14E+03	2.46E+03	2.23E+03	2.13E+03
	Rank	1	12	8	13	2	11	10	4	3	6	9	7	5
C17-F18	Mean	1.83E+03	2.70E+07	2.52E+06	3.11E+07	1.89E+03	3.45E+07	5.61E+06	6.08E+05	3.99E+05	1.58E+06	4.89E+05	1.30E+05	3.46E+06
	Best	1.82E+03	7.78E+06	2.68E+05	1.00E+07	1.87E+03	1.27E+06	1.89E+06	1.53E+05	7.46E+04	7.35E+05	2.74E+05	9.29E+04	2.70E+06
	Worst	1.83E+03	5.25E+07	5.02E+06	6.10E+07	1.91E+03	6.54E+07	1.16E+07	1.65E+06	1.02E+06	1.99E+06	9.53E+05	1.55E+05	5.08E+06
	Std	2.94E+00	2.15E+07	2.43E+06	2.35E+07	1.66E+01	3.88E+07	4.53E+06	7.58E+05	4.86E+05	6.28E+05	3.41E+05	2.95E+04	1.18E+06
	Median	1.83E+03	2.39E+07	2.39E+06	2.66E+07	1.90E+03	3.57E+07	4.48E+06	3.17E+05	2.48E+05	1.80E+06	3.65E+05	1.37E+05	3.04E+06
	Rank	1	11	8	12	2	13	10	6	4	7	5	3	9
C17-F19	Mean	1.91E+03	5.52E+08	6.44E+04	9.30E+08	1.92E+03	2.80E+08	1.36E+07	8.93E+05	3.83E+06	5.47E+06	7.78E+04	4.24E+04	1.54E+06
	Best	1.91E+03	4.13E+08	1.38E+04	6.72E+08	1.92E+03	3.48E+06	1.77E+06	2.26E+04	6.74E+04	2.84E+06	4.22E+04	8.42E+03	6.09E+05
	Worst	1.91E+03	7.18E+08	1.43E+05	1.41E+09	1.93E+03	7.75E+08	2.35E+07	2.01E+06	1.24E+07	7.77E+06	1.05E+05	1.27E+05	2.74E+06
	Std	2.10E+00	1.67E+08	6.14E+04	3.56E+08	3.45E+00	3.88E+08	1.08E+07	1.05E+06	6.23E+06	2.64E+06	2.83E+04	6.14E+04	9.77E+05
	Median	1.91E+03	5.38E+08	5.02E+04	8.20E+08	1.92E+03	1.70E+08	1.46E+07	7.71E+05	1.45E+06	5.63E+06	8.21E+04	1.72E+04	1.41E+06
	Rank	1	12	4	13	2	11	10	6	8	9	5	3	7
C17-F20	Mean	2.07E+03	2.86E+03	2.61E+03	2.91E+03	2.17E+03	2.82E+03	2.80E+03	2.58E+03	2.36E+03	2.77E+03	2.97E+03	2.53E+03	2.46E+03
	Best	2.03E+03	2.77E+03	2.46E+03	2.74E+03	2.06E+03	2.68E+03	2.61E+03	2.36E+03	2.19E+03	2.68E+03	2.61E+03	2.48E+03	2.41E+03
	Worst	2.16E+03	2.97E+03	2.84E+03	3.02E+03	2.26E+03	2.96E+03	2.98E+03	2.97E+03	2.52E+03	2.88E+03	3.43E+03	2.65E+03	2.49E+03
	Std	6.93E+01	8.89E+01	1.78E+02	1.32E+02	9.10E+01	1.28E+02	1.70E+02	2.94E+02	1.47E+02	1.02E+02	3.75E+02	9.11E+01	3.82E+01
	Median	2.04E+03	2.85E+03	2.58E+03	2.95E+03	2.18E+03	2.81E+03	2.81E+03	2.50E+03	2.36E+03	2.75E+03	2.92E+03	2.49E+03	2.46E+03
	Rank	1	11	7	12	2	10	9	6	3	8	13	5	4
C17-F21	Mean	2.31E+03	2.61E+03	2.44E+03	2.66E+03	2.36E+03	2.53E+03	2.60E+03	2.40E+03	2.39E+03	2.49E+03	2.56E+03	2.43E+03	2.49E+03
	Best	2.30E+03	2.52E+03	2.22E+03	2.59E+03	2.35E+03	2.31E+03	2.52E+03	2.37E+03	2.35E+03	2.48E+03	2.54E+03	2.41E+03	2.45E+03
	Worst	2.31E+03	2.67E+03	2.59E+03	2.75E+03	2.38E+03	2.65E+03	2.66E+03	2.43E+03	2.40E+03	2.50E+03	2.59E+03	2.44E+03	2.53E+03
	Std	4.85E+00	7.71E+01	1.67E+02	7.80E+01	1.19E+01	1.66E+02	7.36E+01	2.86E+01	2.49E+01	1.17E+01	2.55E+01	1.70E+01	3.74E+01
	Median	2.31E+03	2.62E+03	2.47E+03	2.66E+03	2.36E+03	2.57E+03	2.60E+03	2.40E+03	2.40E+03	2.49E+03	2.55E+03	2.43E+03	2.48E+03
	Rank	1	12	6	13	2	9	11	4	3	8	10	5	7
C17-F22	Mean	2.30E+03	7.75E+03	5.63E+03	7.51E+03	2.30E+03	8.50E+03	7.19E+03	3.89E+03	2.69E+03	5.54E+03	6.16E+03	4.78E+03	2.69E+03
	Best	2.30E+03	7.42E+03	2.30E+03	6.53E+03	2.30E+03	8.28E+03	6.28E+03	2.31E+03	2.56E+03	2.71E+03	3.93E+03	2.45E+03	2.61E+03
	Worst	2.30E+03	8.25E+03	6.91E+03	8.51E+03	2.30E+03	8.61E+03	8.01E+03	5.85E+03	2.94E+03	8.69E+03	7.14E+03	7.03E+03	2.74E+03
	Std	0.00E+00	3.87E+02	2.42E+03	9.26E+02	1.28E+00	1.67E+02	7.85E+02	2.01E+03	1.88E+02	3.55E+03	1.63E+03	2.29E+03	6.86E+01
	Median	2.30E+03	7.66E+03	6.65E+03	7.51E+03	2.30E+03	8.56E+03	7.24E+03	3.69E+03	2.62E+03	5.39E+03	6.78E+03	4.81E+03	2.69E+03
	Rank	1	12	8	11	2	13	10	5	4	7	9	6	3
C17-F23	Mean	2.66E+03	3.17E+03	2.92E+03	3.23E+03	2.65E+03	3.18E+03	3.03E+03	2.73E+03	2.75E+03	2.90E+03	3.73E+03	2.89E+03	2.96E+03
	Best	2.65E+03	3.09E+03	2.81E+03	3.17E+03	2.48E+03	3.06E+03	2.86E+03	2.69E+03	2.73E+03	2.87E+03	3.62E+03	2.86E+03	2.94E+03
	Worst	2.66E+03	3.25E+03	3.08E+03	3.30E+03	2.71E+03	3.37E+03	3.13E+03	2.76E+03	2.77E+03	2.94E+03	3.83E+03	2.94E+03	3.03E+03
	Std	1.80E+00	8.26E+01	1.30E+02	6.07E+01	1.22E+02	1.46E+02	1.29E+02	3.30E+01	1.84E+01	3.57E+01	1.20E+02	4.10E+01	4.48E+01
	Median	2.65E+03	3.17E+03	2.89E+03	3.21E+03	2.70E+03	3.14E+03	3.07E+03	2.74E+03	2.75E+03	2.88E+03	3.73E+03	2.88E+03	2.95E+03
	Rank	2	10	7	12	1	11	9	3	4	6	13	5	8
C17-F24	Mean	2.83E+03	3.30E+03	3.16E+03	3.39E+03	2.88E+03	3.27E+03	3.11E+03	2.90E+03	2.92E+03	3.03E+03	3.35E+03	3.12E+03	3.21E+03
	Best	2.83E+03	3.26E+03	3.02E+03	3.31E+03	2.87E+03	3.16E+03	3.04E+03	2.86E+03	2.91E+03	3.01E+03	3.31E+03	3.05E+03	3.12E+03
	Worst	2.83E+03	3.37E+03	3.31E+03	3.54E+03	2.89E+03	3.31E+03	3.13E+03	2.93E+03	2.92E+03	3.07E+03	3.38E+03	3.23E+03	3.29E+03
	Std	1.25E+00	5.57E+01	1.36E+02	1.19E+02	1.11E+01	7.88E+01	4.52E+01	3.42E+01	9.27E+00	2.69E+01	3.48E+01	8.56E+01	8.47E+01
	Median	2.83E+03	3.28E+03	3.15E+03	3.36E+03	2.89E+03	3.29E+03	3.12E+03	2.92E+03	2.92E+03	3.03E+03	3.35E+03	3.10E+03	3.22E+03
	Rank	1	11	8	13	2	10	6	3	4	5	12	7	9
C17-F25	Mean	2.89E+03	3.90E+03	2.91E+03	4.50E+03	2.89E+03	3.45E+03	3.07E+03	2.91E+03	2.99E+03	3.07E+03	2.99E+03	2.89E+03	3.10E+03
	Best	2.89E+03	3.54E+03	2.89E+03	3.92E+03	2.88E+03	3.08E+03	3.04E+03	2.88E+03	2.95E+03	2.95E+03	2.98E+03	2.89E+03	3.08E+03
	Worst	2.89E+03	4.17E+03	2.95E+03	5.28E+03	2.90E+03	3.83E+03	3.09E+03	2.97E+03	3.06E+03	3.20E+03	3.00E+03	2.91E+03	3.11E+03
	Std	8.28E-03	2.89E+02	2.74E+01	6.16E+02	6.14E+00	3.95E+02	2.75E+01	4.50E+01	5.30E+01	1.30E+02	1.05E+01	1.21E+01	1.34E+01
	Median	2.89E+03	3.95E+03	2.90E+03	4.41E+03	2.89E+03	3.44E+03	3.08E+03	2.89E+03	2.97E+03	3.06E+03	2.99E+03	2.89E+03	3.10E+03
	Rank	1	12	4	13	2	11	9	5	6	8	7	3	10
C17-F26	Mean	3.58E+03	8.97E+03	7.19E+03	9.53E+03	2.97E+03	8.54E+03	8.19E+03	4.74E+03	4.53E+03	5.84E+03	7.33E+03	4.80E+03	4.36E+03
	Best	3.56E+03	8.56E+03	5.95E+03	8.73E+03	2.97E+03	7.90E+03	7.49E+03	4.41E+03	4.15E+03	4.50E+03	6.31E+03	3.55E+03	3.99E+03
	Worst	3.61E+03	9.71E+03	7.92E+03	1.10E+04	2.98E+03	8.94E+03	9.02E+03	5.35E+03	5.12E+03	7.10E+03	7.85E+03	6.29E+03	4.82E+03
	Std	2.48E+01	5.83E+02	9.42E+02	1.14E+03	2.85E+00	4.85E+02	6.85E+02	4.77E+02	4.51E+02	1.30E+03	7.82E+02	1.40E+03	3.77E+02
	Median	3.57E+03	8.80E+03	7.44E+03	9.22E+03	2.97E+03	8.66E+03	8.14E+03	4.60E+03	4.42E+03	5.87E+03	7.57E+03	4.68E+03	4.32E+03
	Rank	2	12	8	13	1	11	10	5	4	7	9	6	3
C17-F27	Mean	3.21E+03	3.60E+03	3.35E+03	3.75E+03	3.21E+03	3.46E+03	3.42E+03	3.23E+03	3.25E+03	3.31E+03	4.91E+03	3.28E+03	3.45E+03
	Best	3.20E+03	3.54E+03	3.27E+03	3.47E+03	3.20E+03	3.33E+03	3.26E+03	3.21E+03	3.24E+03	3.24E+03	4.47E+03	3.24E+03	3.38E+03
	Worst	3.21E+03	3.69E+03	3.42E+03	4.02E+03	3.23E+03	3.70E+03	3.54E+03	3.26E+03	3.26E+03	3.38E+03	5.23E+03	3.32E+03	3.49E+03
	Std	5.06E+00	7.48E+01	8.95E+01	2.56E+02	1.64E+01	1.80E+02	1.33E+02	1.99E+01	1.10E+01	6.52E+01	4.01E+02	3.71E+01	5.62E+01
	Median	3.21E+03	3.58E+03	3.36E+03	3.74E+03	3.21E+03	3.41E+03	3.44E+03	3.23E+03	3.25E+03	3.32E+03	4.97E+03	3.27E+03	3.47E+03
	Rank	1	11	7	12	2	10	8	3	4	6	13	5	9
C17-F28	Mean	3.10E+03	4.72E+03	3.26E+03	5.60E+03	3.21E+03	4.12E+03	3.43E+03	3.25E+03	3.58E+03	3.65E+03	3.51E+03	3.32E+03	3.57E+03
	Best	3.10E+03	4.49E+03	3.23E+03	5.30E+03	3.19E+03	3.58E+03	3.37E+03	3.22E+03	3.39E+03	3.50E+03	3.44E+03	3.19E+03	3.52E+03
	Worst	3.10E+03	4.97E+03	3.29E+03	5.91E+03	3.24E+03	4.67E+03	3.48E+03	3.28E+03	4.05E+03	3.98E+03	3.65E+03	3.52E+03	3.62E+03
	Std	2.86E-13	2.22E+02	2.71E+01	3.18E+02	2.20E+01	5.49E+02	5.32E+01	2.99E+01	3.43E+02	2.44E+02	1.06E+02	1.66E+02	5.44E+01
	Median	3.10E+03	4.71E+03	3.26E+03	5.59E+03	3.20E+03	4.12E+03	3.43E+03	3.25E+03	3.44E+03	3.56E+03	3.47E+03	3.29E+03	3.56E+03
	Rank	1	12	4	13	2	11	6	3	9	10	7	5	8
C17-F29	Mean	3.35E+03	5.33E+03	4.30E+03	5.54E+03	3.65E+03	5.17E+03	5.03E+03	3.83E+03	3.78E+03	4.47E+03	5.00E+03	4.14E+03	4.25E+03
	Best	3.33E+03	4.89E+03	3.95E+03	4.93E+03	3.50E+03	4.65E+03	4.76E+03	3.70E+03	3.70E+03	4.15E+03	4.74E+03	3.95E+03	3.88E+03
	Worst	3.37E+03	5.79E+03	4.50E+03	6.38E+03	3.78E+03	6.03E+03	5.19E+03	3.94E+03	3.89E+03	4.94E+03	5.25E+03	4.38E+03	4.60E+03
	Std	2.14E+01	4.71E+02	2.65E+02	7.74E+02	1.36E+02	7.04E+02	1.99E+02	1.11E+02	9.50E+01	3.65E+02	2.99E+02	1.93E+02	3.49E+02
	Median	3.36E+03	5.31E+03	4.36E+03	5.43E+03	3.65E+03	5.01E+03	5.07E+03	3.83E+03	3.76E+03	4.40E+03	5.01E+03	4.12E+03	4.27E+03
	Rank	1	12	7	13	2	11	10	4	3	8	9	5	6
C17-F30	Mean	5.01E+03	1.37E+09	1.36E+06	2.70E+09	7.57E+03	3.67E+07	3.75E+07	2.96E+06	6.10E+06	3.62E+07	2.16E+06	2.61E+05	6.71E+05
	Best	4.96E+03	1.01E+09	4.81E+05	1.94E+09	6.32E+03	1.26E+07	7.47E+06	5.31E+05	1.36E+06	1.94E+07	1.89E+06	7.48E+03	1.86E+05
	Worst	5.09E+03	1.50E+09	2.41E+06	2.98E+09	1.00E+04	8.58E+07	6.01E+07	4.23E+06	1.65E+07	7.59E+07	2.60E+06	9.86E+05	1.28E+06
	Std	6.42E+01	2.62E+08	8.79E+05	5.53E+08	1.89E+03	3.62E+07	2.39E+07	1.80E+06	7.59E+06	2.90E+07	3.35E+05	5.27E+05	5.82E+05
	Median	4.99E+03	1.48E+09	1.28E+06	2.94E+09	6.97E+03	2.43E+07	4.12E+07	3.53E+06	3.28E+06	2.47E+07	2.08E+06	2.45E+04	6.07E+05
	Rank	1	12	5	13	2	10	11	7	8	9	6	3	4
Sum rank		31	334	182	361	57	305	284	128	151	232	231	139	204
Mean rank		1.07E+00	1.15E+01	6.28E+00	1.24E+01	1.97E+00	1.05E+01	9.79E+00	4.41E+00	5.21E+00	8.00E+00	7.97E+00	4.79E+00	7.03E+00
Total rank		1	12	6	13	2	11	10	3	5	9	8	4	7

**Table 4:** Optimization results of CEC 2017 test suite (dimension = 50).

		LOA	WSO	AVOA	RSA	MPA	TSA	WOA	MVO	GWO	TLBO	GSA	PSO	GA
C17-F1	Mean	1.00E+02	5.67E+10	8.76E+06	8.87E+10	5.34E+06	3.61E+10	7.29E+09	3.85E+06	8.86E+09	1.96E+10	1.62E+10	2.40E+09	9.85E+09
	Best	1.00E+02	5.06E+10	1.04E+06	7.76E+10	2.06E+06	3.32E+10	4.30E+09	2.76E+06	6.39E+09	1.34E+10	1.30E+10	9.84E+08	9.38E+09
	Worst	1.00E+02	6.06E+10	2.32E+07	9.70E+10	1.35E+07	3.88E+10	1.09E+10	4.79E+06	1.21E+10	2.65E+10	1.94E+10	3.20E+09	1.06E+10
	Std	0.00E+00	4.83E+09	1.07E+07	9.19E+09	5.99E+06	2.53E+09	3.40E+09	9.13E+05	2.60E+09	6.94E+09	2.88E+09	1.06E+09	6.29E+08
	Median	1.00E+02	5.77E+10	5.41E+06	9.02E+10	2.88E+06	3.61E+10	6.98E+09	3.93E+06	8.46E+09	1.94E+10	1.63E+10	2.71E+09	9.71E+09
	Rank	1	12	4	13	3	11	6	2	7	10	9	5	8
C17-F3	Mean	3.00E+02	1.50E+05	1.39E+05	1.49E+05	1.70E+04	1.03E+05	2.21E+05	4.38E+04	1.23E+05	9.31E+04	1.68E+05	1.37E+05	2.49E+05
	Best	3.00E+02	1.29E+05	1.07E+05	1.36E+05	1.47E+04	9.08E+04	1.67E+05	3.47E+04	1.08E+05	7.04E+04	1.52E+05	1.03E+05	2.08E+05
	Worst	3.00E+02	1.73E+05	1.69E+05	1.63E+05	2.01E+04	1.10E+05	3.38E+05	5.45E+04	1.38E+05	1.06E+05	1.90E+05	1.79E+05	2.87E+05
	Std	0.00E+00	2.01E+04	3.06E+04	1.32E+04	2.62E+03	9.75E+03	8.76E+04	8.96E+03	1.33E+04	1.78E+04	2.01E+04	3.57E+04	3.51E+04
	Median	3.00E+02	1.49E+05	1.40E+05	1.50E+05	1.66E+04	1.06E+05	1.91E+05	4.30E+04	1.23E+05	9.78E+04	1.66E+05	1.33E+05	2.52E+05
	Rank	1	10	8	9	2	5	12	3	6	4	11	7	13
C17-F4	Mean	4.70E+02	1.40E+04	6.85E+02	2.25E+04	5.28E+02	7.89E+03	1.86E+03	5.58E+02	1.38E+03	2.67E+03	2.93E+03	9.87E+02	1.47E+03
	Best	4.29E+02	1.09E+04	6.70E+02	1.49E+04	4.92E+02	6.33E+03	1.19E+03	5.21E+02	1.03E+03	1.52E+03	2.45E+03	6.70E+02	1.27E+03
	Worst	5.26E+02	1.59E+04	7.10E+02	2.69E+04	5.80E+02	1.02E+04	2.22E+03	6.30E+02	1.69E+03	4.56E+03	3.11E+03	1.74E+03	1.59E+03
	Std	5.39E+01	2.46E+03	2.01E+01	5.97E+03	4.48E+01	1.78E+03	5.04E+02	5.41E+01	3.21E+02	1.45E+03	3.50E+02	5.51E+02	1.52E+02
	Median	4.64E+02	1.46E+04	6.81E+02	2.41E+04	5.19E+02	7.53E+03	2.02E+03	5.40E+02	1.41E+03	2.30E+03	3.07E+03	7.67E+02	1.51E+03
	Rank	1	12	4	13	2	11	8	3	6	9	10	5	7
C17-F5	Mean	5.05E+02	1.07E+03	8.38E+02	1.09E+03	7.23E+02	1.11E+03	9.31E+02	7.26E+02	7.13E+02	9.72E+02	7.89E+02	7.73E+02	8.71E+02
	Best	5.04E+02	1.04E+03	8.10E+02	1.08E+03	6.46E+02	9.77E+02	8.93E+02	6.56E+02	6.87E+02	9.32E+02	7.40E+02	7.22E+02	8.42E+02
	Worst	5.06E+02	1.11E+03	8.78E+02	1.11E+03	7.84E+02	1.22E+03	9.55E+02	8.33E+02	7.40E+02	9.98E+02	8.24E+02	8.34E+02	8.91E+02
	Std	1.04E+00	3.61E+01	3.19E+01	1.51E+01	6.28E+01	1.28E+02	3.01E+01	8.59E+01	3.07E+01	3.19E+01	4.32E+01	5.03E+01	2.51E+01
	Median	5.04E+02	1.06E+03	8.33E+02	1.10E+03	7.32E+02	1.13E+03	9.38E+02	7.07E+02	7.13E+02	9.78E+02	7.97E+02	7.69E+02	8.75E+02
	Rank	1	11	7	12	3	13	9	4	2	10	6	5	8
C17-F6	Mean	6.00E+02	6.90E+02	6.57E+02	6.92E+02	6.11E+02	6.85E+02	6.92E+02	6.35E+02	6.21E+02	6.61E+02	6.55E+02	6.50E+02	6.46E+02
	Best	6.00E+02	6.87E+02	6.52E+02	6.89E+02	6.08E+02	6.66E+02	6.87E+02	6.26E+02	6.16E+02	6.49E+02	6.50E+02	6.48E+02	6.34E+02
	Worst	6.00E+02	6.94E+02	6.62E+02	6.94E+02	6.14E+02	7.00E+02	7.00E+02	6.58E+02	6.31E+02	6.69E+02	6.57E+02	6.54E+02	6.58E+02
	Std	0.00E+00	3.77E+00	4.87E+00	2.50E+00	2.84E+00	1.69E+01	6.05E+00	1.67E+01	7.15E+00	9.38E+00	3.53E+00	2.71E+00	1.10E+01
	Median	6.00E+02	6.89E+02	6.56E+02	6.91E+02	6.10E+02	6.86E+02	6.91E+02	6.29E+02	6.20E+02	6.62E+02	6.56E+02	6.50E+02	6.46E+02
	Rank	1	11	8	12	2	10	13	4	3	9	7	6	5
C17-F7	Mean	7.57E+02	1.73E+03	1.61E+03	1.83E+03	1.01E+03	1.63E+03	1.65E+03	1.04E+03	1.05E+03	1.44E+03	1.37E+03	1.17E+03	1.28E+03
	Best	7.55E+02	1.71E+03	1.55E+03	1.75E+03	9.60E+02	1.49E+03	1.59E+03	1.00E+03	1.03E+03	1.32E+03	1.21E+03	1.02E+03	1.20E+03
	Worst	7.58E+02	1.76E+03	1.68E+03	1.93E+03	1.06E+03	1.77E+03	1.74E+03	1.07E+03	1.07E+03	1.50E+03	1.50E+03	1.39E+03	1.32E+03
	Std	1.69E+00	2.43E+01	6.01E+01	8.13E+01	5.23E+01	1.45E+02	7.17E+01	2.97E+01	2.03E+01	8.82E+01	1.38E+02	1.74E+02	5.87E+01
	Median	7.57E+02	1.73E+03	1.62E+03	1.82E+03	1.02E+03	1.63E+03	1.64E+03	1.04E+03	1.05E+03	1.47E+03	1.39E+03	1.14E+03	1.29E+03
	Rank	1	12	9	13	2	10	11	3	4	8	7	5	6
C17-F8	Mean	8.06E+02	1.39E+03	1.11E+03	1.41E+03	9.99E+02	1.40E+03	1.30E+03	1.01E+03	1.02E+03	1.29E+03	1.12E+03	1.04E+03	1.23E+03
	Best	8.03E+02	1.33E+03	1.06E+03	1.38E+03	9.70E+02	1.31E+03	1.17E+03	9.72E+02	9.88E+02	1.24E+03	1.11E+03	1.00E+03	1.19E+03
	Worst	8.11E+02	1.43E+03	1.15E+03	1.43E+03	1.03E+03	1.53E+03	1.40E+03	1.08E+03	1.06E+03	1.35E+03	1.14E+03	1.10E+03	1.25E+03
	Std	3.89E+00	4.69E+01	5.47E+01	2.33E+01	3.34E+01	1.04E+02	1.03E+02	5.01E+01	3.36E+01	4.79E+01	1.07E+01	5.30E+01	2.95E+01
	Median	8.04E+02	1.39E+03	1.11E+03	1.42E+03	9.99E+02	1.39E+03	1.31E+03	9.95E+02	1.02E+03	1.29E+03	1.12E+03	1.03E+03	1.24E+03
	Rank	1	11	6	13	2	12	10	3	4	9	7	5	8
C17-F9	Mean	9.00E+02	3.41E+04	1.26E+04	3.43E+04	3.18E+03	3.58E+04	3.12E+04	1.86E+04	6.53E+03	2.27E+04	1.01E+04	9.75E+03	1.21E+04
	Best	9.00E+02	3.28E+04	1.20E+04	3.22E+04	2.01E+03	3.30E+04	2.90E+04	9.96E+03	5.68E+03	1.75E+04	9.21E+03	9.03E+03	9.97E+03
	Worst	9.00E+02	3.73E+04	1.34E+04	3.60E+04	4.59E+03	3.99E+04	3.64E+04	2.46E+04	7.43E+03	2.67E+04	1.09E+04	1.11E+04	1.39E+04
	Std	1.01E-13	2.31E+03	6.61E+02	1.94E+03	1.16E+03	3.24E+03	3.84E+03	7.47E+03	9.87E+02	4.16E+03	7.68E+02	9.97E+02	2.29E+03
	Median	9.00E+02	3.32E+04	1.25E+04	3.45E+04	3.07E+03	3.51E+04	2.96E+04	1.99E+04	6.50E+03	2.33E+04	1.01E+04	9.44E+03	1.23E+04
	Rank	1	11	7	12	2	13	10	8	3	9	5	4	6
C17-F10	Mean	4.35E+03	1.25E+04	8.12E+03	1.37E+04	6.43E+03	1.14E+04	1.14E+04	7.49E+03	8.44E+03	1.35E+04	8.38E+03	7.61E+03	1.13E+04
	Best	3.56E+03	1.20E+04	7.60E+03	1.34E+04	5.59E+03	1.04E+04	1.02E+04	6.21E+03	6.50E+03	1.28E+04	7.56E+03	7.42E+03	1.08E+04
	Worst	5.10E+03	1.33E+04	8.59E+03	1.41E+04	7.04E+03	1.24E+04	1.25E+04	8.53E+03	1.33E+04	1.40E+04	9.45E+03	8.11E+03	1.20E+04
	Std	7.02E+02	6.53E+02	4.50E+02	3.50E+02	7.56E+02	9.28E+02	1.12E+03	1.08E+03	3.58E+03	6.98E+02	8.56E+02	3.62E+02	5.64E+02
	Median	4.37E+03	1.24E+04	8.14E+03	1.36E+04	6.54E+03	1.13E+04	1.14E+04	7.61E+03	6.96E+03	1.36E+04	8.25E+03	7.46E+03	1.12E+04
	Rank	1	11	5	13	2	9	10	3	7	12	6	4	8
C17-F11	Mean	1.13E+03	1.46E+04	1.58E+03	1.99E+04	1.25E+03	1.23E+04	4.88E+03	1.55E+03	5.86E+03	4.90E+03	1.35E+04	1.64E+03	2.28E+04
	Best	1.12E+03	1.35E+04	1.47E+03	1.77E+04	1.20E+03	1.06E+04	4.31E+03	1.40E+03	3.54E+03	4.60E+03	1.27E+04	1.38E+03	1.33E+04
	Worst	1.13E+03	1.53E+04	1.72E+03	2.16E+04	1.28E+03	1.47E+04	6.09E+03	1.69E+03	1.01E+04	5.44E+03	1.53E+04	1.95E+03	3.05E+04
	Std	5.92E+00	9.01E+02	1.30E+02	1.75E+03	3.65E+01	1.96E+03	8.94E+02	1.36E+02	3.31E+03	4.25E+02	1.31E+03	2.64E+02	7.74E+03
	Median	1.13E+03	1.48E+04	1.56E+03	2.02E+04	1.26E+03	1.19E+04	4.56E+03	1.54E+03	4.88E+03	4.77E+03	1.30E+04	1.62E+03	2.36E+04
	Rank	1	11	4	12	2	9	6	3	8	7	10	5	13
C17-F12	Mean	2.91E+03	4.13E+10	6.95E+07	6.74E+10	1.36E+07	2.45E+10	1.25E+09	7.51E+07	9.08E+08	4.79E+09	2.06E+09	1.52E+09	1.94E+08
	Best	2.53E+03	3.47E+10	2.95E+07	4.92E+10	1.29E+07	1.03E+10	1.03E+09	4.04E+07	1.43E+08	2.70E+09	6.77E+08	1.20E+07	6.11E+07
	Worst	3.17E+03	4.96E+10	1.07E+08	9.25E+10	1.43E+07	4.12E+10	1.70E+09	1.19E+08	1.69E+09	9.42E+09	3.70E+09	4.39E+09	2.68E+08
	Std	2.98E+02	7.29E+09	4.55E+07	2.17E+10	7.27E+05	1.39E+10	3.35E+08	3.61E+07	8.38E+08	3.43E+09	1.36E+09	2.22E+09	9.90E+07
	Median	2.96E+03	4.05E+10	7.06E+07	6.40E+10	1.37E+07	2.32E+10	1.13E+09	7.02E+07	9.01E+08	3.52E+09	1.93E+09	8.38E+08	2.23E+08
	Rank	1	12	3	13	2	11	7	4	6	10	9	8	5
C17-F13	Mean	1.34E+03	2.33E+10	1.41E+05	4.08E+10	1.55E+04	9.56E+09	9.00E+07	2.29E+05	3.39E+08	5.55E+08	1.76E+07	4.52E+08	3.93E+07
	Best	1.33E+03	1.34E+10	3.25E+04	2.06E+10	8.26E+03	5.08E+09	6.76E+07	1.43E+05	1.54E+08	4.52E+08	2.97E+04	4.84E+04	2.56E+07
	Worst	1.34E+03	3.18E+10	3.11E+05	5.87E+10	1.83E+04	1.49E+10	1.02E+08	3.57E+05	8.51E+08	7.58E+08	5.92E+07	1.14E+09	5.26E+07
	Std	4.66E+00	8.77E+09	1.30E+05	1.74E+10	5.29E+03	4.51E+09	1.66E+07	9.91E+04	3.72E+08	1.50E+08	3.07E+07	6.06E+08	1.31E+07
	Median	1.34E+03	2.40E+10	1.11E+05	4.20E+10	1.78E+04	9.14E+09	9.50E+07	2.08E+05	1.75E+08	5.04E+08	5.51E+06	3.34E+08	3.96E+07
	Rank	1	12	3	13	2	11	7	4	8	10	5	9	6
C17-F14	Mean	1.43E+03	2.46E+07	1.16E+06	4.59E+07	1.56E+03	2.55E+06	4.52E+06	1.81E+05	1.09E+06	8.21E+05	1.44E+07	5.44E+05	1.06E+07
	Best	1.43E+03	8.04E+06	3.59E+05	1.41E+07	1.54E+03	6.73E+05	4.00E+06	1.15E+05	8.51E+04	6.77E+05	3.26E+06	1.96E+05	5.23E+06
	Worst	1.43E+03	4.82E+07	2.76E+06	9.29E+07	1.58E+03	4.04E+06	5.37E+06	3.51E+05	2.11E+06	9.47E+05	2.36E+07	8.72E+05	1.83E+07
	Std	2.85E+00	1.84E+07	1.19E+06	3.65E+07	1.79E+01	1.52E+06	6.44E+05	1.24E+05	8.99E+05	1.53E+05	1.00E+07	3.01E+05	6.00E+06
	Median	1.43E+03	2.11E+07	7.59E+05	3.83E+07	1.55E+03	2.74E+06	4.36E+06	1.29E+05	1.09E+06	8.30E+05	1.53E+07	5.55E+05	9.50E+06
	Rank	1	12	7	13	2	8	9	3	6	5	11	4	10
C17-F15	Mean	1.53E+03	2.47E+09	3.61E+04	3.97E+09	2.22E+03	1.62E+09	9.41E+06	1.15E+05	5.64E+06	6.69E+07	1.87E+08	1.04E+04	8.13E+06
	Best	1.53E+03	1.75E+09	2.23E+04	3.10E+09	2.10E+03	5.56E+08	8.67E+05	4.77E+04	4.02E+04	3.92E+07	1.82E+04	2.71E+03	2.76E+06
	Worst	1.53E+03	3.24E+09	6.64E+04	4.70E+09	2.36E+03	3.52E+09	1.76E+07	1.72E+05	1.49E+07	8.71E+07	7.27E+08	2.03E+04	1.77E+07
	Std	3.19E+00	7.62E+08	2.23E+04	7.73E+08	1.53E+02	1.50E+09	7.99E+06	6.00E+04	7.04E+06	2.18E+07	3.92E+08	8.51E+03	7.17E+06
	Median	1.53E+03	2.45E+09	2.79E+04	4.04E+09	2.22E+03	1.20E+09	9.60E+06	1.21E+05	3.83E+06	7.07E+07	1.13E+07	9.24E+03	6.06E+06
	Rank	1	12	4	13	2	11	8	5	6	9	10	3	7
C17-F16	Mean	2.06E+03	6.07E+03	4.24E+03	7.30E+03	2.72E+03	4.51E+03	5.32E+03	3.26E+03	3.26E+03	4.42E+03	3.86E+03	3.28E+03	3.82E+03
	Best	1.73E+03	5.27E+03	3.91E+03	5.50E+03	2.57E+03	3.96E+03	4.37E+03	3.04E+03	2.89E+03	4.03E+03	3.54E+03	2.89E+03	3.22E+03
	Worst	2.24E+03	7.73E+03	4.64E+03	1.09E+04	2.98E+03	4.80E+03	5.95E+03	3.50E+03	3.82E+03	4.69E+03	4.25E+03	3.71E+03	4.33E+03
	Std	2.53E+02	1.25E+03	3.74E+02	2.68E+03	2.10E+02	4.14E+02	7.55E+02	2.06E+02	4.93E+02	3.05E+02	3.76E+02	4.47E+02	5.23E+02
	Median	2.14E+03	5.63E+03	4.20E+03	6.42E+03	2.66E+03	4.64E+03	5.47E+03	3.25E+03	3.17E+03	4.47E+03	3.82E+03	3.26E+03	3.86E+03
	Rank	1	12	8	13	2	10	11	4	3	9	7	5	6
C17-F17	Mean	2.02E+03	7.33E+03	3.48E+03	1.06E+04	2.53E+03	3.86E+03	4.41E+03	3.02E+03	2.92E+03	4.04E+03	3.73E+03	3.28E+03	3.50E+03
	Best	1.90E+03	5.60E+03	3.05E+03	7.75E+03	2.46E+03	3.11E+03	3.95E+03	2.49E+03	2.78E+03	3.43E+03	3.29E+03	3.07E+03	3.28E+03
	Worst	2.14E+03	8.97E+03	3.99E+03	1.37E+04	2.59E+03	4.30E+03	4.62E+03	3.48E+03	3.18E+03	4.41E+03	4.02E+03	3.60E+03	3.74E+03
	Std	1.46E+02	1.51E+03	4.82E+02	2.68E+03	5.97E+01	5.64E+02	3.40E+02	4.47E+02	1.97E+02	4.70E+02	3.45E+02	2.75E+02	2.33E+02
	Median	2.02E+03	7.38E+03	3.44E+03	1.04E+04	2.54E+03	4.01E+03	4.52E+03	3.05E+03	2.86E+03	4.16E+03	3.80E+03	3.23E+03	3.50E+03
	Rank	1	12	6	13	2	9	11	4	3	10	8	5	7
C17-F18	Mean	1.83E+03	7.19E+07	2.29E+06	1.07E+08	2.50E+04	3.33E+07	4.29E+07	2.51E+06	5.43E+06	7.78E+06	7.98E+06	7.82E+05	8.99E+06
	Best	1.82E+03	5.75E+07	2.97E+05	4.79E+07	3.65E+03	2.99E+06	1.16E+07	1.48E+06	1.04E+06	5.35E+06	3.77E+06	3.34E+05	3.22E+06
	Worst	1.84E+03	8.47E+07	4.19E+06	1.48E+08	3.73E+04	9.51E+07	7.76E+07	3.90E+06	1.08E+07	1.08E+07	1.49E+07	1.28E+06	2.16E+07
	Std	8.86E+00	1.28E+07	2.15E+06	5.35E+07	1.60E+04	4.60E+07	3.55E+07	1.26E+06	5.57E+06	2.52E+06	5.53E+06	4.74E+05	9.24E+06
	Median	1.83E+03	7.26E+07	2.33E+06	1.15E+08	2.95E+04	1.75E+07	4.11E+07	2.32E+06	4.93E+06	7.48E+06	6.62E+06	7.57E+05	5.56E+06
	Rank	1	12	4	13	2	10	11	5	6	7	8	3	9
C17-F19	Mean	1.93E+03	2.59E+09	2.47E+05	3.64E+09	2.07E+03	2.54E+09	6.49E+06	4.86E+06	1.10E+06	4.81E+07	4.29E+05	3.74E+05	9.41E+05
	Best	1.92E+03	1.23E+09	8.66E+04	2.46E+09	2.02E+03	9.28E+06	9.77E+05	3.70E+06	5.40E+05	4.09E+07	2.47E+05	2.85E+03	7.36E+05
	Worst	1.93E+03	4.32E+09	5.09E+05	4.51E+09	2.10E+03	7.41E+09	1.53E+07	6.03E+06	1.70E+06	6.11E+07	9.40E+05	9.33E+05	1.28E+06
	Std	8.61E-01	1.41E+09	1.99E+05	9.92E+08	4.32E+01	3.61E+09	6.70E+06	1.04E+06	5.26E+05	9.81E+06	3.71E+05	4.83E+05	2.77E+05
	Median	1.93E+03	2.40E+09	1.96E+05	3.81E+09	2.09E+03	1.36E+09	4.85E+06	4.86E+06	1.09E+06	4.53E+07	2.65E+05	2.79E+05	8.77E+05
	Rank	1	12	3	13	2	11	9	8	7	10	5	4	6
C17-F20	Mean	2.16E+03	3.74E+03	3.21E+03	4.00E+03	2.63E+03	3.37E+03	3.67E+03	3.22E+03	2.60E+03	3.69E+03	3.95E+03	3.23E+03	3.12E+03
	Best	2.10E+03	3.42E+03	2.65E+03	3.73E+03	2.36E+03	2.94E+03	3.38E+03	3.00E+03	2.41E+03	3.57E+03	3.68E+03	2.84E+03	3.05E+03
	Worst	2.32E+03	3.91E+03	3.72E+03	4.17E+03	2.90E+03	3.58E+03	4.23E+03	3.67E+03	2.80E+03	3.86E+03	4.21E+03	3.40E+03	3.24E+03
	Std	1.19E+02	2.45E+02	4.98E+02	2.05E+02	2.48E+02	3.20E+02	4.18E+02	3.34E+02	2.25E+02	1.33E+02	2.37E+02	2.85E+02	9.08E+01
	Median	2.11E+03	3.82E+03	3.24E+03	4.05E+03	2.64E+03	3.48E+03	3.54E+03	3.11E+03	2.60E+03	3.67E+03	3.95E+03	3.34E+03	3.09E+03
	Rank	1	11	5	13	3	8	9	6	2	10	12	7	4
C17-F21	Mean	2.31E+03	2.96E+03	2.73E+03	3.00E+03	2.44E+03	2.93E+03	2.92E+03	2.56E+03	2.51E+03	2.80E+03	2.82E+03	2.64E+03	2.73E+03
	Best	2.31E+03	2.93E+03	2.62E+03	2.90E+03	2.42E+03	2.83E+03	2.81E+03	2.53E+03	2.46E+03	2.77E+03	2.75E+03	2.57E+03	2.71E+03
	Worst	2.33E+03	2.99E+03	2.91E+03	3.08E+03	2.47E+03	3.09E+03	3.01E+03	2.60E+03	2.55E+03	2.84E+03	2.85E+03	2.74E+03	2.75E+03
	Std	1.08E+01	3.72E+01	1.39E+02	9.48E+01	2.35E+01	1.23E+02	9.36E+01	3.92E+01	4.30E+01	3.31E+01	5.19E+01	8.23E+01	2.19E+01
	Median	2.31E+03	2.96E+03	2.70E+03	3.01E+03	2.44E+03	2.90E+03	2.93E+03	2.56E+03	2.52E+03	2.79E+03	2.83E+03	2.63E+03	2.73E+03
	Rank	1	12	7	13	2	11	10	4	3	8	9	5	6
C17-F22	Mean	3.10E+03	1.44E+04	1.08E+04	1.56E+04	5.25E+03	1.32E+04	1.32E+04	8.71E+03	8.59E+03	1.51E+04	1.10E+04	9.44E+03	8.56E+03
	Best	2.30E+03	1.41E+04	8.50E+03	1.54E+04	2.32E+03	1.28E+04	1.25E+04	6.91E+03	7.56E+03	1.46E+04	1.07E+04	8.63E+03	3.95E+03
	Worst	5.48E+03	1.46E+04	1.24E+04	1.59E+04	8.24E+03	1.38E+04	1.35E+04	9.92E+03	9.10E+03	1.56E+04	1.15E+04	9.90E+03	1.30E+04
	Std	1.73E+03	2.52E+02	2.00E+03	2.96E+02	3.47E+03	4.65E+02	4.62E+02	1.40E+03	7.69E+02	5.32E+02	3.54E+02	6.47E+02	5.49E+03
	Median	2.30E+03	1.45E+04	1.11E+04	1.56E+04	5.21E+03	1.32E+04	1.33E+04	9.01E+03	8.86E+03	1.51E+04	1.10E+04	9.61E+03	8.62E+03
	Rank	1	11	7	13	2	10	9	5	4	12	8	6	3
C17-F23	Mean	2.74E+03	3.78E+03	3.27E+03	3.85E+03	2.88E+03	3.70E+03	3.70E+03	2.98E+03	3.01E+03	3.26E+03	4.67E+03	3.35E+03	3.34E+03
	Best	2.73E+03	3.70E+03	3.19E+03	3.80E+03	2.87E+03	3.50E+03	3.53E+03	2.94E+03	2.93E+03	3.17E+03	4.49E+03	3.29E+03	3.21E+03
	Worst	2.75E+03	3.87E+03	3.35E+03	3.89E+03	2.90E+03	4.03E+03	3.80E+03	3.05E+03	3.14E+03	3.33E+03	4.84E+03	3.41E+03	3.47E+03
	Std	1.09E+01	8.13E+01	8.26E+01	3.88E+01	1.50E+01	2.74E+02	1.33E+02	5.70E+01	1.01E+02	6.83E+01	1.57E+02	6.98E+01	1.16E+02
	Median	2.75E+03	3.77E+03	3.27E+03	3.85E+03	2.88E+03	3.64E+03	3.74E+03	2.96E+03	2.98E+03	3.27E+03	4.68E+03	3.36E+03	3.33E+03
	Rank	1	11	6	12	2	9	10	3	4	5	13	8	7
C17-F24	Mean	2.92E+03	4.16E+03	3.49E+03	4.43E+03	3.06E+03	3.96E+03	3.80E+03	3.13E+03	3.19E+03	3.43E+03	4.33E+03	3.44E+03	3.64E+03
	Best	2.91E+03	3.92E+03	3.38E+03	3.96E+03	3.03E+03	3.87E+03	3.69E+03	3.09E+03	3.09E+03	3.35E+03	4.29E+03	3.29E+03	3.60E+03
	Worst	2.92E+03	4.71E+03	3.67E+03	5.58E+03	3.10E+03	4.10E+03	3.85E+03	3.16E+03	3.31E+03	3.49E+03	4.38E+03	3.59E+03	3.73E+03
	Std	7.43E+00	4.03E+02	1.35E+02	8.43E+02	3.19E+01	1.14E+02	7.99E+01	3.35E+01	1.00E+02	6.69E+01	4.30E+01	1.48E+02	6.94E+01
	Median	2.92E+03	4.01E+03	3.45E+03	4.09E+03	3.06E+03	3.94E+03	3.82E+03	3.13E+03	3.17E+03	3.44E+03	4.32E+03	3.44E+03	3.61E+03
	Rank	1	11	7	13	2	10	9	3	4	5	12	6	8
C17-F25	Mean	2.98E+03	8.37E+03	3.17E+03	1.16E+04	3.06E+03	5.88E+03	4.11E+03	3.05E+03	3.99E+03	4.32E+03	4.22E+03	3.12E+03	4.00E+03
	Best	2.98E+03	6.92E+03	3.14E+03	9.32E+03	3.04E+03	4.81E+03	3.71E+03	3.02E+03	3.80E+03	3.85E+03	3.89E+03	3.07E+03	3.90E+03
	Worst	2.99E+03	9.30E+03	3.21E+03	1.30E+04	3.08E+03	6.91E+03	4.40E+03	3.07E+03	4.19E+03	4.89E+03	4.86E+03	3.16E+03	4.12E+03
	Std	6.30E+00	1.15E+03	3.36E+01	1.86E+03	1.69E+01	9.85E+02	3.19E+02	2.56E+01	2.18E+02	5.69E+02	4.96E+02	5.01E+01	9.93E+01
	Median	2.98E+03	8.64E+03	3.16E+03	1.20E+04	3.07E+03	5.91E+03	4.15E+03	3.06E+03	3.99E+03	4.27E+03	4.07E+03	3.11E+03	4.00E+03
	Rank	1	12	5	13	3	11	8	2	6	10	9	4	7
C17-F26	Mean	3.78E+03	1.37E+04	1.07E+04	1.46E+04	3.35E+03	1.23E+04	1.34E+04	5.71E+03	6.41E+03	9.49E+03	1.12E+04	7.96E+03	8.79E+03
	Best	3.75E+03	1.35E+04	1.02E+04	1.40E+04	3.15E+03	1.02E+04	1.25E+04	5.24E+03	6.03E+03	8.72E+03	1.09E+04	7.40E+03	6.99E+03
	Worst	3.79E+03	1.39E+04	1.12E+04	1.56E+04	3.62E+03	1.35E+04	1.51E+04	5.97E+03	6.76E+03	1.02E+04	1.16E+04	8.50E+03	1.11E+04
	Std	2.12E+01	2.07E+02	4.39E+02	7.14E+02	2.34E+02	1.54E+03	1.23E+03	3.59E+02	4.15E+02	6.87E+02	3.29E+02	5.35E+02	2.14E+03
	Median	3.78E+03	1.37E+04	1.07E+04	1.45E+04	3.30E+03	1.27E+04	1.30E+04	5.82E+03	6.42E+03	9.52E+03	1.12E+04	7.96E+03	8.52E+03
	Rank	2	12	8	13	1	10	11	3	4	7	9	5	6
C17-F27	Mean	3.25E+03	4.74E+03	3.83E+03	4.92E+03	3.38E+03	4.65E+03	4.41E+03	3.36E+03	3.63E+03	3.81E+03	7.90E+03	3.63E+03	4.40E+03
	Best	3.23E+03	4.43E+03	3.78E+03	4.56E+03	3.27E+03	3.97E+03	3.86E+03	3.32E+03	3.58E+03	3.62E+03	7.66E+03	3.38E+03	4.29E+03
	Worst	3.31E+03	4.95E+03	3.89E+03	5.18E+03	3.47E+03	5.13E+03	4.97E+03	3.43E+03	3.67E+03	3.97E+03	8.25E+03	3.87E+03	4.54E+03
	Std	4.54E+01	2.48E+02	5.37E+01	3.23E+02	8.98E+01	5.52E+02	5.67E+02	5.14E+01	5.21E+01	1.70E+02	3.11E+02	2.42E+02	1.14E+02
	Median	3.23E+03	4.79E+03	3.82E+03	4.97E+03	3.38E+03	4.76E+03	4.41E+03	3.34E+03	3.62E+03	3.82E+03	7.85E+03	3.64E+03	4.38E+03
	Rank	1	11	7	12	3	10	9	2	4	6	13	5	8
C17-F28	Mean	3.26E+03	8.51E+03	3.58E+03	1.09E+04	3.35E+03	7.10E+03	4.76E+03	3.28E+03	4.36E+03	5.17E+03	4.99E+03	3.85E+03	4.97E+03
	Best	3.26E+03	7.69E+03	3.50E+03	9.63E+03	3.31E+03	5.77E+03	4.17E+03	3.26E+03	4.10E+03	4.57E+03	4.93E+03	3.54E+03	4.73E+03
	Worst	3.26E+03	1.06E+04	3.67E+03	1.41E+04	3.39E+03	8.46E+03	4.98E+03	3.30E+03	4.69E+03	5.70E+03	5.11E+03	4.34E+03	5.15E+03
	Std	0.00E+00	1.52E+03	8.91E+01	2.38E+03	4.21E+01	1.48E+03	4.26E+02	2.10E+01	2.99E+02	5.03E+02	8.60E+01	3.78E+02	2.24E+02
	Median	3.26E+03	7.89E+03	3.58E+03	9.85E+03	3.34E+03	7.08E+03	4.94E+03	3.29E+03	4.32E+03	5.21E+03	4.96E+03	3.75E+03	5.00E+03
	Rank	1	12	4	13	3	11	7	2	6	10	9	5	8
C17-F29	Mean	3.26E+03	1.32E+04	5.42E+03	1.89E+04	4.06E+03	6.76E+03	8.82E+03	4.78E+03	4.81E+03	6.41E+03	7.99E+03	4.78E+03	6.04E+03
	Best	3.25E+03	8.76E+03	5.28E+03	1.00E+04	3.72E+03	6.33E+03	5.98E+03	4.35E+03	4.62E+03	5.54E+03	6.60E+03	4.56E+03	5.74E+03
	Worst	3.28E+03	1.81E+04	5.55E+03	2.98E+04	4.30E+03	7.27E+03	1.15E+04	5.36E+03	5.10E+03	7.36E+03	1.04E+04	4.86E+03	6.62E+03
	Std	1.90E+01	4.68E+03	1.22E+02	9.56E+03	2.85E+02	4.23E+02	2.48E+03	4.59E+02	2.43E+02	9.41E+02	1.88E+03	1.61E+02	4.49E+02
	Median	3.26E+03	1.30E+04	5.42E+03	1.78E+04	4.12E+03	6.72E+03	8.89E+03	4.70E+03	4.77E+03	6.37E+03	7.46E+03	4.85E+03	5.90E+03
	Rank	1	12	6	13	2	9	11	3	5	8	10	4	7
C17-F30	Mean	6.24E+05	3.11E+09	2.08E+07	5.22E+09	1.61E+06	1.58E+09	1.51E+08	6.70E+07	1.32E+08	2.85E+08	1.75E+08	4.60E+06	5.56E+07
	Best	5.82E+05	2.40E+09	1.27E+07	3.20E+09	1.22E+06	1.93E+08	1.02E+08	6.06E+07	6.41E+07	1.99E+08	1.34E+08	3.22E+06	4.48E+07
	Worst	6.56E+05	4.22E+09	2.85E+07	8.19E+09	2.60E+06	3.20E+09	2.08E+08	7.71E+07	1.97E+08	3.61E+08	2.30E+08	6.39E+06	7.80E+07
	Std	3.56E+04	8.65E+08	8.44E+06	2.34E+09	7.24E+05	1.68E+09	5.79E+07	7.80E+06	7.28E+07	7.41E+07	4.36E+07	1.70E+06	1.67E+07
	Median	6.28E+05	2.91E+09	2.10E+07	4.74E+09	1.30E+06	1.46E+09	1.47E+08	6.52E+07	1.35E+08	2.91E+08	1.69E+08	4.40E+06	4.97E+07
	Rank	1	12	4	13	2	11	8	6	7	10	9	3	5
Sum rank		30	335	166	367	63	294	269	112	144	248	254	150	207
Mean rank		1.03E+00	1.16E+01	5.72E+00	1.27E+01	2.17E+00	1.01E+01	9.28E+00	3.86E+00	4.97E+00	8.55E+00	8.76E+00	5.17E+00	7.14E+00
Total rank		1	12	6	13	2	11	10	3	4	8	9	5	7

**Table 5:** Optimization results of CEC 2017 test suite (dimension = 100).

		LOA	WSO	AVOA	RSA	MPA	TSA	WOA	MVO	GWO	TLBO	GSA	PSO	GA
C17-F1	Mean	1.00E+02	1.58E+11	3.63E+09	2.21E+11	4.93E+08	1.20E+11	5.95E+10	6.24E+07	5.42E+10	8.65E+10	1.29E+11	1.90E+10	5.32E+10
	Best	1.00E+02	1.55E+11	1.77E+09	2.17E+11	3.73E+08	1.05E+11	5.62E+10	5.20E+07	4.70E+10	8.24E+10	1.19E+11	1.28E+10	5.04E+10
	Worst	1.00E+02	1.62E+11	5.22E+09	2.23E+11	6.23E+08	1.34E+11	6.66E+10	7.31E+07	6.13E+10	9.53E+10	1.38E+11	2.59E+10	6.02E+10
	Std	1.26E-14	3.53E+09	1.55E+09	2.77E+09	1.31E+08	1.28E+10	5.21E+09	1.12E+07	7.42E+09	6.52E+09	8.97E+09	7.81E+09	5.06E+09
	Median	1.00E+02	1.58E+11	3.77E+09	2.22E+11	4.88E+08	1.20E+11	5.76E+10	6.23E+07	5.43E+10	8.42E+10	1.30E+11	1.87E+10	5.12E+10
	Rank	1	12	4	13	3	10	8	2	7	9	11	5	6
C17-F3	Mean	3.00E+02	4.06E+05	3.10E+05	3.06E+05	1.50E+05	3.45E+05	7.48E+05	4.42E+05	3.49E+05	2.81E+05	3.26E+05	5.13E+05	5.47E+05
	Best	3.00E+02	3.70E+05	3.02E+05	2.95E+05	1.15E+05	2.76E+05	6.55E+05	3.67E+05	3.20E+05	2.63E+05	3.01E+05	3.89E+05	5.25E+05
	Worst	3.00E+02	4.25E+05	3.17E+05	3.12E+05	1.81E+05	3.94E+05	8.67E+05	5.29E+05	3.82E+05	2.97E+05	3.56E+05	7.20E+05	5.65E+05
	Std	0.00E+00	2.78E+04	6.52E+03	8.76E+03	3.15E+04	5.41E+04	9.93E+04	8.97E+04	3.66E+04	1.50E+04	2.48E+04	1.67E+05	1.93E+04
	Median	3.00E+02	4.15E+05	3.10E+05	3.08E+05	1.52E+05	3.55E+05	7.36E+05	4.36E+05	3.47E+05	2.81E+05	3.22E+05	4.72E+05	5.50E+05
	Rank	1	9	5	4	2	7	13	10	8	3	6	11	12
C17-F4	Mean	6.02E+02	4.23E+04	1.50E+03	7.12E+04	9.97E+02	1.52E+04	1.04E+04	7.52E+02	4.27E+03	1.02E+04	3.24E+04	2.38E+03	8.76E+03
	Best	5.92E+02	3.89E+04	1.27E+03	6.46E+04	8.90E+02	9.96E+03	8.87E+03	7.00E+02	3.28E+03	9.73E+03	2.57E+04	1.45E+03	8.28E+03
	Worst	6.12E+02	4.63E+04	1.65E+03	7.94E+04	1.11E+03	2.02E+04	1.14E+04	8.09E+02	6.40E+03	1.10E+04	3.66E+04	2.99E+03	9.30E+03
	Std	1.27E+01	3.48E+03	1.91E+02	6.67E+03	1.15E+02	4.61E+03	1.18E+03	4.97E+01	1.56E+03	6.77E+02	5.72E+03	7.22E+02	5.19E+02
	Median	6.02E+02	4.19E+04	1.55E+03	7.05E+04	9.95E+02	1.53E+04	1.07E+04	7.50E+02	3.69E+03	1.00E+04	3.35E+04	2.53E+03	8.72E+03
	Rank	1	12	4	13	3	10	9	2	6	8	11	5	7
C17-F5	Mean	5.13E+02	1.88E+03	1.25E+03	1.85E+03	1.16E+03	2.02E+03	1.74E+03	1.17E+03	1.13E+03	1.77E+03	1.27E+03	1.34E+03	1.50E+03
	Best	5.11E+02	1.86E+03	1.24E+03	1.82E+03	1.05E+03	2.00E+03	1.65E+03	1.07E+03	1.07E+03	1.74E+03	1.24E+03	1.25E+03	1.36E+03
	Worst	5.15E+02	1.89E+03	1.26E+03	1.88E+03	1.24E+03	2.05E+03	1.88E+03	1.24E+03	1.17E+03	1.80E+03	1.30E+03	1.50E+03	1.58E+03
	Std	1.98E+00	1.35E+01	9.10E+00	3.60E+01	1.05E+02	2.55E+01	1.10E+02	8.22E+01	4.68E+01	2.36E+01	3.48E+01	1.30E+02	1.07E+02
	Median	5.13E+02	1.88E+03	1.25E+03	1.85E+03	1.18E+03	2.02E+03	1.71E+03	1.19E+03	1.13E+03	1.77E+03	1.27E+03	1.31E+03	1.53E+03
	Rank	1	12	5	11	3	13	9	4	2	10	6	7	8
C17-F6	Mean	6.00E+02	6.98E+02	6.57E+02	6.97E+02	6.34E+02	7.02E+02	6.96E+02	6.69E+02	6.37E+02	6.75E+02	6.59E+02	6.57E+02	6.58E+02
	Best	6.00E+02	6.96E+02	6.53E+02	6.92E+02	6.31E+02	6.91E+02	6.87E+02	6.63E+02	6.32E+02	6.67E+02	6.57E+02	6.50E+02	6.51E+02
	Worst	6.00E+02	7.01E+02	6.61E+02	6.99E+02	6.40E+02	7.10E+02	7.12E+02	6.75E+02	6.43E+02	6.80E+02	6.63E+02	6.62E+02	6.63E+02
	Std	0.00E+00	2.38E+00	3.45E+00	3.50E+00	4.91E+00	1.01E+01	1.22E+01	5.64E+00	4.95E+00	6.80E+00	3.03E+00	6.27E+00	6.55E+00
	Median	6.00E+02	6.98E+02	6.57E+02	6.98E+02	6.33E+02	7.04E+02	6.92E+02	6.69E+02	6.36E+02	6.77E+02	6.58E+02	6.57E+02	6.59E+02
	Rank	1	12	5	11	2	13	10	8	3	9	7	4	6
C17-F7	Mean	8.11E+02	3.39E+03	2.90E+03	3.50E+03	1.76E+03	3.23E+03	3.37E+03	1.91E+03	1.92E+03	2.92E+03	2.94E+03	2.34E+03	2.43E+03
	Best	8.10E+02	3.31E+03	2.75E+03	3.41E+03	1.70E+03	3.06E+03	3.25E+03	1.76E+03	1.75E+03	2.78E+03	2.82E+03	2.10E+03	2.34E+03
	Worst	8.13E+02	3.49E+03	3.03E+03	3.57E+03	1.83E+03	3.39E+03	3.53E+03	2.02E+03	2.05E+03	3.03E+03	3.14E+03	2.45E+03	2.64E+03
	Std	1.59E+00	7.87E+01	1.48E+02	7.32E+01	6.10E+01	1.59E+02	1.38E+02	1.20E+02	1.39E+02	1.10E+02	1.53E+02	1.85E+02	1.50E+02
	Median	8.11E+02	3.39E+03	2.91E+03	3.51E+03	1.75E+03	3.24E+03	3.34E+03	1.93E+03	1.95E+03	2.93E+03	2.90E+03	2.41E+03	2.38E+03
	Rank	1	12	7	13	2	10	11	3	4	8	9	5	6
C17-F8	Mean	8.12E+02	2.29E+03	1.66E+03	2.34E+03	1.38E+03	2.27E+03	2.20E+03	1.40E+03	1.46E+03	2.14E+03	1.74E+03	1.63E+03	1.93E+03
	Best	8.09E+02	2.24E+03	1.61E+03	2.32E+03	1.22E+03	2.21E+03	2.01E+03	1.26E+03	1.36E+03	2.08E+03	1.67E+03	1.59E+03	1.89E+03
	Worst	8.17E+02	2.35E+03	1.69E+03	2.36E+03	1.48E+03	2.35E+03	2.34E+03	1.57E+03	1.59E+03	2.19E+03	1.86E+03	1.72E+03	1.98E+03
	Std	3.70E+00	4.80E+01	3.89E+01	1.75E+01	1.23E+02	7.57E+01	1.83E+02	1.39E+02	1.12E+02	5.14E+01	9.59E+01	6.43E+01	4.44E+01
	Median	8.12E+02	2.29E+03	1.68E+03	2.35E+03	1.41E+03	2.26E+03	2.22E+03	1.39E+03	1.44E+03	2.14E+03	1.72E+03	1.61E+03	1.93E+03
	Rank	1	12	6	13	2	11	10	3	4	9	7	5	8
C17-F9	Mean	9.00E+02	8.26E+04	2.43E+04	7.08E+04	2.06E+04	1.10E+05	7.04E+04	5.43E+04	3.30E+04	6.82E+04	2.16E+04	3.01E+04	4.22E+04
	Best	9.00E+02	7.37E+04	2.02E+04	6.85E+04	1.92E+04	9.05E+04	5.47E+04	4.58E+04	2.04E+04	6.53E+04	2.01E+04	2.55E+04	3.82E+04
	Worst	9.00E+02	9.54E+04	2.74E+04	7.27E+04	2.12E+04	1.38E+05	8.87E+04	6.17E+04	4.48E+04	6.98E+04	2.28E+04	3.36E+04	4.75E+04
	Std	1.01E-13	1.02E+04	3.23E+03	2.04E+03	1.04E+03	2.16E+04	1.84E+04	7.15E+03	1.30E+04	2.19E+03	1.21E+03	3.91E+03	4.25E+03
	Median	9.00E+02	8.06E+04	2.48E+04	7.10E+04	2.10E+04	1.07E+05	6.90E+04	5.48E+04	3.33E+04	6.89E+04	2.18E+04	3.08E+04	4.15E+04
	Rank	1	12	4	11	2	13	10	8	6	9	3	5	7
C17-F10	Mean	1.10E+04	2.88E+04	1.56E+04	3.00E+04	1.36E+04	2.80E+04	2.70E+04	1.65E+04	1.49E+04	3.01E+04	1.67E+04	1.66E+04	2.49E+04
	Best	9.63E+03	2.86E+04	1.32E+04	2.92E+04	1.30E+04	2.73E+04	2.62E+04	1.59E+04	1.38E+04	2.88E+04	1.51E+04	1.49E+04	2.44E+04
	Worst	1.19E+04	2.91E+04	1.77E+04	3.05E+04	1.45E+04	2.89E+04	2.83E+04	1.71E+04	1.54E+04	3.11E+04	1.77E+04	1.77E+04	2.55E+04
	Std	1.05E+03	2.81E+02	2.17E+03	6.46E+02	6.78E+02	7.51E+02	1.04E+03	5.37E+02	8.17E+02	1.01E+03	1.31E+03	1.29E+03	4.78E+02
	Median	1.13E+04	2.88E+04	1.57E+04	3.02E+04	1.36E+04	2.79E+04	2.67E+04	1.65E+04	1.51E+04	3.02E+04	1.71E+04	1.68E+04	2.49E+04
	Rank	1	11	4	12	2	10	9	5	3	13	7	6	8
C17-F11	Mean	1.16E+03	1.53E+05	5.97E+04	1.92E+05	4.54E+03	6.09E+04	1.94E+05	4.35E+03	8.11E+04	6.68E+04	1.61E+05	4.85E+04	1.30E+05
	Best	1.14E+03	1.19E+05	5.36E+04	1.47E+05	3.59E+03	2.78E+04	1.13E+05	3.79E+03	6.74E+04	5.64E+04	1.34E+05	2.21E+04	9.89E+04
	Worst	1.22E+03	1.78E+05	7.13E+04	2.74E+05	5.41E+03	8.70E+04	3.13E+05	4.61E+03	9.14E+04	8.52E+04	1.87E+05	9.90E+04	1.79E+05
	Std	4.25E+01	2.77E+04	8.90E+03	6.21E+04	8.54E+02	2.67E+04	1.01E+05	4.07E+02	1.12E+04	1.37E+04	2.41E+04	3.74E+04	3.80E+04
	Median	1.14E+03	1.58E+05	5.69E+04	1.74E+05	4.57E+03	6.43E+04	1.75E+05	4.50E+03	8.29E+04	6.29E+04	1.61E+05	3.64E+04	1.20E+05
	Rank	1	10	5	12	3	6	13	2	8	7	11	4	9
C17-F12	Mean	5.97E+03	9.81E+10	6.12E+08	1.60E+11	2.42E+08	5.29E+10	1.23E+10	3.09E+08	1.06E+10	2.04E+10	6.22E+10	9.39E+09	1.15E+10
	Best	5.38E+03	6.97E+10	3.25E+08	1.19E+11	1.35E+08	2.71E+10	9.95E+09	1.96E+08	7.37E+09	1.60E+10	5.39E+10	1.22E+09	1.05E+10
	Worst	6.57E+03	1.09E+11	9.78E+08	1.86E+11	2.91E+08	8.77E+10	1.40E+10	4.86E+08	1.27E+10	2.81E+10	7.31E+10	1.79E+10	1.36E+10
	Std	5.38E+02	2.07E+10	3.06E+08	3.30E+10	7.84E+07	2.75E+10	1.86E+09	1.39E+08	2.48E+09	6.01E+09	8.70E+09	8.24E+09	1.54E+09
	Median	5.97E+03	1.07E+11	5.73E+08	1.67E+11	2.72E+08	4.83E+10	1.26E+10	2.77E+08	1.13E+10	1.87E+10	6.08E+10	9.24E+09	1.10E+10
	Rank	1	12	4	13	2	10	8	3	6	9	11	5	7
C17-F13	Mean	1.41E+03	2.59E+10	9.15E+04	3.97E+10	9.03E+04	1.99E+10	4.87E+08	3.30E+05	8.82E+08	2.62E+09	8.13E+09	1.64E+09	1.63E+08
	Best	1.37E+03	2.26E+10	6.47E+04	3.07E+10	3.87E+04	1.41E+10	3.46E+08	2.91E+05	7.60E+07	1.81E+09	5.00E+09	1.81E+08	1.27E+08
	Worst	1.44E+03	2.87E+10	1.25E+05	4.50E+10	2.24E+05	2.38E+10	6.58E+08	3.84E+05	2.33E+09	3.17E+09	1.04E+10	2.97E+09	1.96E+08
	Std	3.78E+01	3.51E+09	2.77E+04	7.19E+09	9.76E+04	4.46E+09	1.75E+08	4.47E+04	1.13E+09	6.74E+08	2.48E+09	1.49E+09	3.85E+07
	Median	1.41E+03	2.62E+10	8.83E+04	4.16E+10	4.91E+04	2.08E+10	4.71E+08	3.22E+05	5.60E+08	2.75E+09	8.54E+09	1.71E+09	1.64E+08
	Rank	1	12	3	13	2	11	6	4	7	9	10	8	5
C17-F14	Mean	1.47E+03	4.23E+07	6.22E+06	7.43E+07	8.48E+04	8.29E+06	1.36E+07	2.83E+06	8.97E+06	1.30E+07	1.07E+07	7.60E+05	9.79E+06
	Best	1.46E+03	3.66E+07	3.77E+06	6.77E+07	2.43E+04	3.77E+06	7.81E+06	8.54E+05	5.67E+06	9.66E+06	8.26E+06	3.61E+05	5.48E+06
	Worst	1.47E+03	4.84E+07	1.03E+07	8.13E+07	1.80E+05	1.62E+07	1.85E+07	3.89E+06	1.34E+07	1.66E+07	1.61E+07	1.58E+06	1.44E+07
	Std	6.58E+00	5.64E+06	3.14E+06	7.09E+06	7.59E+04	5.95E+06	4.80E+06	1.47E+06	3.71E+06	3.93E+06	3.93E+06	6.03E+05	4.05E+06
	Median	1.47E+03	4.22E+07	5.39E+06	7.40E+07	6.74E+04	6.61E+06	1.40E+07	3.28E+06	8.38E+06	1.28E+07	9.27E+06	5.50E+05	9.63E+06
	Rank	1	12	5	13	2	6	11	4	7	10	9	3	8
C17-F15	Mean	1.61E+03	1.44E+10	7.87E+04	2.19E+10	5.23E+04	1.12E+10	6.54E+07	1.18E+05	4.68E+08	1.11E+09	1.16E+09	3.11E+08	1.18E+07
	Best	1.55E+03	1.33E+10	6.44E+04	1.57E+10	1.52E+04	2.33E+08	3.64E+07	8.08E+04	3.07E+07	3.71E+08	4.64E+08	5.74E+04	7.63E+06
	Worst	1.65E+03	1.61E+10	9.88E+04	2.73E+10	7.94E+04	2.11E+10	1.26E+08	1.74E+05	1.40E+09	2.37E+09	1.48E+09	1.23E+09	2.02E+07
	Std	4.80E+01	1.35E+09	1.79E+04	6.30E+09	2.95E+04	9.84E+09	4.43E+07	4.45E+04	6.90E+08	9.55E+08	5.12E+08	6.65E+08	6.19E+06
	Median	1.62E+03	1.40E+10	7.59E+04	2.24E+10	5.73E+04	1.18E+10	4.97E+07	1.09E+05	2.19E+08	8.50E+08	1.34E+09	8.05E+06	9.77E+06
	Rank	1	12	3	13	2	11	6	4	8	9	10	7	5
C17-F16	Mean	2.71E+03	1.79E+04	6.84E+03	2.13E+04	5.34E+03	1.38E+04	1.53E+04	6.34E+03	5.88E+03	1.09E+04	1.05E+04	6.24E+03	1.00E+04
	Best	2.17E+03	1.66E+04	5.75E+03	1.68E+04	5.25E+03	1.14E+04	1.25E+04	5.63E+03	5.32E+03	1.04E+04	9.11E+03	5.99E+03	9.06E+03
	Worst	3.40E+03	1.84E+04	7.53E+03	2.38E+04	5.47E+03	1.65E+04	1.70E+04	6.81E+03	6.51E+03	1.19E+04	1.21E+04	6.44E+03	1.08E+04
	Std	5.55E+02	8.89E+02	8.41E+02	3.47E+03	1.07E+02	2.29E+03	2.17E+03	5.68E+02	6.74E+02	7.72E+02	1.47E+03	2.03E+02	8.46E+02
	Median	2.64E+03	1.82E+04	7.04E+03	2.23E+04	5.32E+03	1.36E+04	1.59E+04	6.46E+03	5.84E+03	1.07E+04	1.04E+04	6.26E+03	1.01E+04
	Rank	1	12	6	13	2	10	11	5	3	9	8	4	7
C17-F17	Mean	2.72E+03	3.94E+06	5.63E+03	7.75E+06	4.52E+03	2.04E+05	1.61E+04	4.82E+03	5.32E+03	8.34E+03	4.35E+04	5.87E+03	6.86E+03
	Best	2.28E+03	1.15E+06	5.42E+03	2.10E+06	4.29E+03	9.70E+03	9.93E+03	4.39E+03	4.31E+03	8.21E+03	2.86E+04	5.62E+03	6.70E+03
	Worst	3.43E+03	8.96E+06	6.07E+03	1.78E+07	4.72E+03	5.43E+05	2.71E+04	5.14E+03	6.86E+03	8.51E+03	7.06E+04	6.09E+03	7.02E+03
	Std	5.60E+02	4.00E+06	3.31E+02	8.05E+06	2.31E+02	2.53E+05	8.42E+03	4.10E+02	1.23E+03	1.58E+02	2.02E+04	2.19E+02	1.46E+02
	Median	2.58E+03	2.82E+06	5.52E+03	5.53E+06	4.53E+03	1.33E+05	1.36E+04	4.87E+03	5.05E+03	8.32E+03	3.74E+04	5.88E+03	6.86E+03
	Rank	1	12	5	13	2	11	9	3	4	8	10	6	7
C17-F18	Mean	1.90E+03	5.45E+07	2.63E+06	9.62E+07	2.17E+05	1.39E+07	1.12E+07	4.58E+06	1.02E+07	1.51E+07	1.10E+07	6.01E+06	5.64E+06
	Best	1.88E+03	2.47E+07	1.31E+06	3.73E+07	1.51E+05	5.21E+06	8.33E+06	3.39E+06	3.22E+06	1.11E+07	5.06E+06	3.71E+06	4.52E+06
	Worst	1.92E+03	9.86E+07	4.16E+06	1.76E+08	3.91E+05	2.84E+07	1.33E+07	7.70E+06	1.65E+07	2.14E+07	2.44E+07	8.66E+06	8.16E+06
	Std	2.11E+01	3.43E+07	1.40E+06	6.35E+07	1.26E+05	1.14E+07	2.45E+06	2.27E+06	5.96E+06	4.78E+06	9.92E+06	2.50E+06	1.86E+06
	Median	1.91E+03	4.74E+07	2.53E+06	8.58E+07	1.63E+05	1.10E+07	1.16E+07	3.62E+06	1.06E+07	1.40E+07	7.22E+06	5.83E+06	4.93E+06
	Rank	1	12	3	13	2	10	9	4	7	11	8	6	5
C17-F19	Mean	1.97E+03	1.19E+10	2.69E+06	2.09E+10	2.62E+05	4.72E+09	1.25E+08	1.55E+07	3.37E+08	6.25E+08	1.48E+09	2.52E+08	1.20E+07
	Best	1.97E+03	1.05E+10	1.03E+06	1.53E+10	5.51E+04	2.09E+09	4.97E+07	9.07E+06	2.67E+06	2.71E+08	2.66E+08	4.19E+07	6.10E+06
	Worst	1.98E+03	1.40E+10	4.95E+06	2.60E+10	4.43E+05	9.37E+09	2.11E+08	2.47E+07	1.01E+09	1.44E+09	2.79E+09	5.45E+08	2.16E+07
	Std	4.94E+00	1.72E+09	1.80E+06	4.83E+09	1.75E+05	3.50E+09	8.13E+07	8.40E+06	5.13E+08	5.96E+08	1.37E+09	2.65E+08	7.49E+06
	Median	1.97E+03	1.15E+10	2.39E+06	2.11E+10	2.74E+05	3.70E+09	1.20E+08	1.42E+07	1.66E+08	3.96E+08	1.43E+09	2.10E+08	1.00E+07
	Rank	1	12	3	13	2	11	6	5	8	9	10	7	4
C17-F20	Mean	3.19E+03	7.03E+03	5.99E+03	7.27E+03	4.42E+03	6.79E+03	6.80E+03	5.65E+03	5.90E+03	7.00E+03	6.13E+03	5.24E+03	6.08E+03
	Best	2.81E+03	6.84E+03	5.66E+03	7.16E+03	4.35E+03	6.19E+03	6.40E+03	5.36E+03	4.73E+03	6.22E+03	5.71E+03	4.54E+03	5.50E+03
	Worst	3.66E+03	7.22E+03	6.25E+03	7.36E+03	4.47E+03	7.53E+03	7.17E+03	6.15E+03	6.77E+03	7.32E+03	6.37E+03	6.07E+03	6.53E+03
	Std	4.78E+02	1.74E+02	3.09E+02	9.01E+01	5.49E+01	6.30E+02	3.68E+02	3.76E+02	1.10E+03	5.64E+02	3.19E+02	7.14E+02	5.37E+02
	Median	3.15E+03	7.04E+03	6.03E+03	7.28E+03	4.42E+03	6.72E+03	6.83E+03	5.55E+03	6.05E+03	7.22E+03	6.22E+03	5.18E+03	6.15E+03
	Rank	1	12	6	13	2	9	10	4	5	11	8	3	7
C17-F21	Mean	2.34E+03	4.16E+03	3.58E+03	4.27E+03	2.80E+03	4.00E+03	4.10E+03	3.18E+03	2.93E+03	3.62E+03	4.56E+03	3.50E+03	3.35E+03
	Best	2.34E+03	4.11E+03	3.38E+03	4.20E+03	2.76E+03	3.87E+03	3.82E+03	3.11E+03	2.86E+03	3.46E+03	4.03E+03	3.32E+03	3.31E+03
	Worst	2.35E+03	4.22E+03	3.71E+03	4.33E+03	2.83E+03	4.09E+03	4.32E+03	3.30E+03	2.98E+03	3.79E+03	4.98E+03	3.83E+03	3.39E+03
	Std	3.66E+00	5.75E+01	1.54E+02	5.95E+01	3.45E+01	1.22E+02	2.44E+02	9.18E+01	5.94E+01	1.50E+02	4.27E+02	2.52E+02	3.79E+01
	Median	2.34E+03	4.15E+03	3.61E+03	4.28E+03	2.80E+03	4.02E+03	4.13E+03	3.15E+03	2.95E+03	3.61E+03	4.61E+03	3.42E+03	3.34E+03
	Rank	1	11	7	12	2	9	10	4	3	8	13	6	5
C17-F22	Mean	1.17E+04	3.04E+04	1.97E+04	3.20E+04	1.82E+04	2.95E+04	2.80E+04	1.69E+04	2.26E+04	3.19E+04	2.05E+04	2.13E+04	2.77E+04
	Best	1.11E+04	2.96E+04	1.84E+04	3.17E+04	1.70E+04	2.84E+04	2.65E+04	1.60E+04	1.81E+04	3.09E+04	1.99E+04	1.99E+04	2.67E+04
	Worst	1.26E+04	3.09E+04	2.14E+04	3.26E+04	1.98E+04	3.06E+04	2.91E+04	1.76E+04	3.31E+04	3.24E+04	2.09E+04	2.28E+04	2.84E+04
	Std	7.10E+02	6.29E+02	1.49E+03	4.70E+02	1.29E+03	9.79E+02	1.26E+03	8.66E+02	7.72E+03	7.06E+02	5.13E+02	1.29E+03	9.16E+02
	Median	1.16E+04	3.06E+04	1.95E+04	3.19E+04	1.81E+04	2.95E+04	2.81E+04	1.71E+04	1.96E+04	3.21E+04	2.07E+04	2.12E+04	2.78E+04
	Rank	1	11	4	13	3	10	9	2	7	12	5	6	8
C17-F23	Mean	2.88E+03	5.19E+03	4.04E+03	5.19E+03	3.27E+03	5.30E+03	5.01E+03	3.45E+03	3.58E+03	4.13E+03	7.60E+03	4.75E+03	4.18E+03
	Best	2.87E+03	4.95E+03	3.96E+03	4.94E+03	3.26E+03	4.58E+03	4.88E+03	3.36E+03	3.54E+03	4.08E+03	7.03E+03	4.26E+03	4.12E+03
	Worst	2.88E+03	5.47E+03	4.12E+03	5.39E+03	3.30E+03	6.28E+03	5.15E+03	3.56E+03	3.62E+03	4.21E+03	8.00E+03	5.01E+03	4.24E+03
	Std	5.67E+00	2.53E+02	8.09E+01	2.05E+02	2.19E+01	8.27E+02	1.42E+02	9.21E+01	3.71E+01	5.79E+01	4.75E+02	3.71E+02	7.40E+01
	Median	2.88E+03	5.17E+03	4.04E+03	5.22E+03	3.26E+03	5.18E+03	5.01E+03	3.44E+03	3.57E+03	4.12E+03	7.68E+03	4.87E+03	4.18E+03
	Rank	1	10	5	11	2	12	9	3	4	6	13	8	7
C17-F24	Mean	3.33E+03	8.25E+03	5.26E+03	1.01E+04	3.70E+03	6.49E+03	6.22E+03	3.93E+03	4.24E+03	4.68E+03	1.04E+04	5.82E+03	5.27E+03
	Best	3.30E+03	6.47E+03	5.06E+03	6.83E+03	3.65E+03	6.03E+03	5.82E+03	3.87E+03	4.02E+03	4.45E+03	9.80E+03	5.46E+03	5.18E+03
	Worst	3.36E+03	9.47E+03	5.44E+03	1.23E+04	3.76E+03	6.80E+03	6.83E+03	4.04E+03	4.45E+03	4.90E+03	1.21E+04	6.28E+03	5.43E+03
	Std	3.22E+01	1.56E+03	1.84E+02	2.89E+03	5.64E+01	3.60E+02	4.80E+02	8.80E+01	2.42E+02	1.97E+02	1.19E+03	3.94E+02	1.22E+02
	Median	3.33E+03	8.54E+03	5.28E+03	1.07E+04	3.69E+03	6.57E+03	6.11E+03	3.92E+03	4.25E+03	4.68E+03	9.91E+03	5.77E+03	5.23E+03
	Rank	1	11	6	12	2	10	9	3	4	5	13	8	7
C17-F25	Mean	3.19E+03	1.46E+04	4.09E+03	2.04E+04	3.66E+03	1.01E+04	7.09E+03	3.40E+03	6.26E+03	8.60E+03	1.06E+04	4.09E+03	7.64E+03
	Best	3.14E+03	1.39E+04	3.73E+03	1.89E+04	3.49E+03	9.46E+03	6.49E+03	3.33E+03	6.11E+03	7.43E+03	9.80E+03	3.83E+03	6.96E+03
	Worst	3.26E+03	1.63E+04	4.43E+03	2.36E+04	3.78E+03	1.05E+04	7.45E+03	3.46E+03	6.64E+03	1.02E+04	1.21E+04	4.50E+03	8.33E+03
	Std	6.52E+01	1.23E+03	3.13E+02	2.44E+03	1.32E+02	5.06E+02	4.70E+02	5.90E+01	2.79E+02	1.36E+03	1.09E+03	3.45E+02	7.74E+02
	Median	3.17E+03	1.41E+04	4.10E+03	1.95E+04	3.68E+03	1.02E+04	7.20E+03	3.40E+03	6.14E+03	8.41E+03	1.03E+04	4.02E+03	7.63E+03
	Rank	1	12	4	13	3	10	7	2	6	9	11	5	8
C17-F26	Mean	5.76E+03	3.77E+04	2.36E+04	4.33E+04	1.13E+04	3.18E+04	3.24E+04	1.15E+04	1.63E+04	2.29E+04	3.23E+04	1.99E+04	2.21E+04
	Best	5.65E+03	3.72E+04	2.09E+04	4.09E+04	1.06E+04	3.06E+04	2.91E+04	1.02E+04	1.45E+04	1.88E+04	3.10E+04	1.79E+04	2.05E+04
	Worst	5.84E+03	3.82E+04	2.64E+04	4.48E+04	1.20E+04	3.26E+04	3.52E+04	1.38E+04	1.78E+04	2.81E+04	3.41E+04	2.18E+04	2.31E+04
	Std	9.13E+01	4.56E+02	2.55E+03	2.05E+03	7.54E+02	9.11E+02	3.30E+03	1.70E+03	1.52E+03	4.22E+03	1.40E+03	1.81E+03	1.21E+03
	Median	5.77E+03	3.77E+04	2.36E+04	4.38E+04	1.13E+04	3.21E+04	3.27E+04	1.10E+04	1.64E+04	2.23E+04	3.21E+04	2.00E+04	2.23E+04
	Rank	1	12	8	13	2	9	11	3	4	7	10	5	6
C17-F27	Mean	3.31E+03	9.02E+03	4.12E+03	1.18E+04	3.52E+03	6.44E+03	5.87E+03	3.61E+03	4.04E+03	4.28E+03	1.35E+04	4.04E+03	5.38E+03
	Best	3.28E+03	7.62E+03	3.96E+03	8.89E+03	3.49E+03	6.15E+03	5.20E+03	3.57E+03	3.88E+03	4.01E+03	1.32E+04	3.84E+03	5.13E+03
	Worst	3.34E+03	1.04E+04	4.39E+03	1.49E+04	3.56E+03	6.80E+03	6.62E+03	3.70E+03	4.17E+03	4.72E+03	1.38E+04	4.23E+03	5.75E+03
	Std	3.09E+01	1.67E+03	2.06E+02	3.51E+03	3.07E+01	3.03E+02	8.31E+02	6.76E+01	1.56E+02	3.40E+02	2.90E+02	2.33E+02	2.91E+02
	Median	3.31E+03	9.02E+03	4.07E+03	1.18E+04	3.53E+03	6.40E+03	5.83E+03	3.58E+03	4.06E+03	4.19E+03	1.35E+04	4.04E+03	5.31E+03
	Rank	1	11	6	12	2	10	9	3	5	7	13	4	8
C17-F28	Mean	3.32E+03	2.00E+04	4.64E+03	2.70E+04	3.75E+03	1.51E+04	1.00E+04	3.45E+03	8.97E+03	1.08E+04	1.80E+04	7.43E+03	1.11E+04
	Best	3.32E+03	1.86E+04	4.35E+03	2.42E+04	3.63E+03	1.19E+04	8.58E+03	3.37E+03	7.62E+03	8.45E+03	1.56E+04	5.08E+03	1.01E+04
	Worst	3.33E+03	2.26E+04	4.85E+03	3.05E+04	3.83E+03	1.75E+04	1.10E+04	3.53E+03	1.09E+04	1.28E+04	1.99E+04	1.14E+04	1.22E+04
	Std	4.77E+00	1.93E+03	2.31E+02	2.87E+03	9.29E+01	2.95E+03	1.10E+03	7.10E+01	1.51E+03	2.22E+03	1.96E+03	3.13E+03	1.20E+03
	Median	3.32E+03	1.94E+04	4.68E+03	2.66E+04	3.77E+03	1.55E+04	1.03E+04	3.45E+03	8.68E+03	1.09E+04	1.83E+04	6.61E+03	1.10E+04
	Rank	1	12	4	13	3	10	7	2	6	8	11	5	9
C17-F29	Mean	4.45E+03	1.74E+05	9.35E+03	3.31E+05	6.75E+03	1.77E+04	1.59E+04	8.46E+03	8.10E+03	1.20E+04	2.39E+04	8.42E+03	1.14E+04
	Best	4.17E+03	9.93E+04	8.13E+03	1.78E+05	5.96E+03	1.36E+04	1.33E+04	7.58E+03	7.92E+03	1.12E+04	1.97E+04	7.79E+03	1.12E+04
	Worst	4.83E+03	2.38E+05	1.01E+04	4.60E+05	7.49E+03	2.24E+04	1.82E+04	9.07E+03	8.39E+03	1.26E+04	3.12E+04	9.27E+03	1.19E+04
	Std	3.07E+02	6.41E+04	9.19E+02	1.31E+05	6.82E+02	3.97E+03	2.63E+03	7.04E+02	2.26E+02	6.49E+02	5.83E+03	7.60E+02	3.29E+02
	Median	4.40E+03	1.80E+05	9.60E+03	3.44E+05	6.78E+03	1.74E+04	1.61E+04	8.59E+03	8.04E+03	1.21E+04	2.22E+04	8.31E+03	1.13E+04
	Rank	1	12	6	13	2	10	9	5	3	8	11	4	7
C17-F30	Mean	5.41E+03	2.19E+10	2.62E+07	3.57E+10	4.44E+06	1.27E+10	1.42E+09	9.73E+07	1.74E+09	3.58E+09	6.95E+09	5.72E+08	6.29E+08
	Best	5.34E+03	1.92E+10	1.49E+07	3.33E+10	1.98E+06	7.71E+09	1.16E+09	5.99E+07	7.13E+08	1.35E+09	4.96E+09	1.39E+08	5.25E+08
	Worst	5.56E+03	2.38E+10	4.61E+07	3.86E+10	7.25E+06	1.57E+10	1.92E+09	1.20E+08	2.27E+09	6.64E+09	8.41E+09	1.77E+09	6.75E+08
	Std	1.10E+02	2.10E+09	1.52E+07	2.45E+09	2.65E+06	3.80E+09	3.73E+08	2.90E+07	7.62E+08	2.89E+09	1.59E+09	8.72E+08	7.63E+07
	Median	5.37E+03	2.23E+10	2.19E+07	3.54E+10	4.27E+06	1.36E+10	1.30E+09	1.05E+08	1.98E+09	3.17E+09	7.21E+09	1.88E+08	6.59E+08
	Rank	1	12	3	13	2	11	7	4	8	9	10	5	6
Sum rank		29	336	140	355	65	293	265	114	156	249	272	162	203
Mean rank		1.00E+00	1.16E+01	4.83E+00	1.22E+01	2.24E+00	1.01E+01	9.14E+00	3.93E+00	5.38E+00	8.59E+00	9.38E+00	5.59E+00	7.00E+00
Total rank		1	12	4	13	2	11	9	3	5	8	10	6	7

**Table 6:** Wilcoxon rank sum test results.

Compared Algorithm	Objective Function Type			
	CEC 2017			
	D = 10	D = 30	D = 50	D = 100
LOA vs. WSO	1.73E-21	1.73E-21	1.73E-21	1.73E-21
LOA vs. AVOA	3.32E-19	2.66E-21	1.73E-21	1.73E-21
LOA vs. RSA	1.73E-21	1.73E-21	1.73E-21	1.73E-21
LOA vs. MPA	1.76E-18	1.37E-16	5.83E-18	1.73E-21
LOA vs. TSA	8.36E-21	1.73E-21	1.73E-21	1.73E-21
LOA vs. WOA	8.36E-21	1.73E-21	1.73E-21	1.73E-21
LOA vs. MVO	7.95E-19	1.87E-21	1.73E-21	1.73E-21
LOA vs. GWO	4.60E-21	1.73E-21	1.73E-21	1.73E-21
LOA vs. TLBO	3.25E-21	1.73E-21	1.73E-21	1.73E-21
LOA vs. GSA	1.41E-18	1.78E-21	1.73E-21	1.73E-21
LOA vs. PSO	1.36E-19	2.07E-21	1.73E-21	1.73E-21
LOA vs. GA	2.38E-19	1.73E-21	1.73E-21	1.73E-21

**Table 7:** Optimization results of CEC 2011 test suite.

		LOA	WSO	AVOA	RSA	MPA	TSA	WOA	MVO	GWO	TLBO	GSA	PSO	GA
C11-F1	Mean	5.92E+00	1.77E+01	1.30E+01	2.20E+01	7.57E+00	1.85E+01	1.33E+01	1.40E+01	1.09E+01	1.85E+01	2.17E+01	1.80E+01	2.34E+01
	Best	2.00E-10	1.54E+01	8.87E+00	2.02E+01	3.72E-01	1.76E+01	8.24E+00	1.17E+01	1.12E+00	1.71E+01	1.96E+01	1.05E+01	2.23E+01
	Worst	1.23E+01	2.06E+01	1.69E+01	2.45E+01	1.27E+01	1.99E+01	1.74E+01	1.61E+01	1.74E+01	2.00E+01	2.33E+01	2.43E+01	2.56E+01
	Std	7.20E+00	2.70E+00	4.69E+00	2.24E+00	5.95E+00	1.09E+00	4.44E+00	2.33E+00	7.31E+00	1.25E+00	1.61E+00	6.72E+00	1.56E+00
	Median	5.69E+00	1.75E+01	1.31E+01	2.17E+01	8.62E+00	1.82E+01	1.38E+01	1.42E+01	1.25E+01	1.85E+01	2.20E+01	1.86E+01	2.29E+01
	Rank	1	7	4	12	2	9	5	6	3	10	11	8	13
C11-F2	Mean	−2.63E+01	−1.45E+01	−2.11E+01	−1.17E+01	−2.51E+01	−1.14E+01	−1.87E+01	−8.93E+00	−2.27E+01	−1.10E+01	−1.56E+01	−2.27E+01	−1.30E+01
	Best	−2.71E+01	−1.58E+01	−2.16E+01	−1.21E+01	−2.57E+01	−1.51E+01	−2.21E+01	−1.09E+01	−2.47E+01	−1.22E+01	−2.07E+01	−2.41E+01	−1.53E+01
	Worst	−2.54E+01	−1.33E+01	−2.04E+01	−1.12E+01	−2.38E+01	−9.23E+00	−1.47E+01	−7.41E+00	−1.91E+01	−9.95E+00	−1.16E+01	−2.04E+01	−1.14E+01
	Std	7.39E-01	1.34E+00	5.79E-01	5.04E-01	9.52E-01	2.91E+00	3.97E+00	1.62E+00	2.62E+00	9.84E-01	4.33E+00	1.69E+00	1.96E+00
	Median	−2.64E+01	−1.44E+01	−2.12E+01	−1.17E+01	−2.54E+01	−1.06E+01	−1.90E+01	−8.68E+00	−2.34E+01	−1.09E+01	−1.51E+01	−2.32E+01	−1.27E+01
	Rank	1	8	5	10	2	11	6	13	4	12	7	3	9
C11-F4	Mean	1.15E-05	1.15E-05	1.15E-05	1.15E-05	1.15E-05	1.15E-05	1.15E-05	1.15E-05	1.15E-05	1.15E-05	1.15E-05	1.15E-05	1.15E-05
	Best	1.15E-05	1.15E-05	1.15E-05	1.15E-05	1.15E-05	1.15E-05	1.15E-05	1.15E-05	1.15E-05	1.15E-05	1.15E-05	1.15E-05	1.15E-05
	Worst	1.15E-05	1.15E-05	1.15E-05	1.15E-05	1.15E-05	1.15E-05	1.15E-05	1.15E-05	1.15E-05	1.15E-05	1.15E-05	1.15E-05	1.15E-05
	Std	2.00E-19	2.18E-11	2.49E-09	4.89E-11	1.22E-15	2.34E-14	5.97E-19	9.76E-13	3.65E-15	7.68E-14	1.96E-19	6.05E-20	2.70E-18
	Median	1.15E-05	1.15E-05	1.15E-05	1.15E-05	1.15E-05	1.15E-05	1.15E-05	1.15E-05	1.15E-05	1.15E-05	1.15E-05	1.15E-05	1.15E-05
	Rank	1	11	13	12	6	8	4	10	7	9	3	2	5
C11-F4	Mean	0.00E+00	0.00E+00	0.00E+00	0.00E+00	0.00E+00	0.00E+00	0.00E+00	0.00E+00	0.00E+00	0.00E+00	0.00E+00	0.00E+00	0.00E+00
	Best	0.00E+00	0.00E+00	0.00E+00	0.00E+00	0.00E+00	0.00E+00	0.00E+00	0.00E+00	0.00E+00	0.00E+00	0.00E+00	0.00E+00	0.00E+00
	Worst	0.00E+00	0.00E+00	0.00E+00	0.00E+00	0.00E+00	0.00E+00	0.00E+00	0.00E+00	0.00E+00	0.00E+00	0.00E+00	0.00E+00	0.00E+00
	Std	0.00E+00	0.00E+00	0.00E+00	0.00E+00	0.00E+00	0.00E+00	0.00E+00	0.00E+00	0.00E+00	0.00E+00	0.00E+00	0.00E+00	0.00E+00
	Median	0.00E+00	0.00E+00	0.00E+00	0.00E+00	0.00E+00	0.00E+00	0.00E+00	0.00E+00	0.00E+00	0.00E+00	0.00E+00	0.00E+00	0.00E+00
	Rank	1	1	1	1	1	1	1	1	1	1	1	1	1
C11-F5	Mean	−3.41E+01	−2.50E+01	−2.82E+01	−2.02E+01	−3.33E+01	−2.72E+01	−2.77E+01	−2.71E+01	−3.16E+01	−1.11E+01	−2.75E+01	−8.97E+00	−9.82E+00
	Best	−3.47E+01	−2.61E+01	−2.93E+01	−2.23E+01	−3.39E+01	−3.16E+01	−2.79E+01	−3.18E+01	−3.42E+01	−1.32E+01	−3.16E+01	−1.25E+01	−1.12E+01
	Worst	−3.34E+01	−2.40E+01	−2.78E+01	−1.79E+01	−3.20E+01	−2.20E+01	−2.73E+01	−2.47E+01	−2.77E+01	−9.51E+00	−2.43E+01	−7.28E+00	−8.18E+00
	Std	5.90E-01	9.31E-01	7.64E-01	2.48E+00	9.20E-01	4.15E+00	2.88E-01	3.48E+00	2.93E+00	1.65E+00	3.33E+00	2.58E+00	1.42E+00
	Median	−3.42E+01	−2.48E+01	−2.79E+01	−2.03E+01	−3.36E+01	−2.77E+01	−2.79E+01	−2.60E+01	−3.23E+01	−1.09E+01	−2.70E+01	−8.06E+00	−9.93E+00
	Rank	1	9	4	10	2	7	5	8	3	11	6	13	12
C11-F6	Mean	−2.41E+01	−1.42E+01	−1.91E+01	−1.32E+01	−2.26E+01	−7.80E+00	−2.00E+01	−9.74E+00	−1.97E+01	−2.63E+00	−2.19E+01	−3.48E+00	−4.38E+00
	Best	−2.74E+01	−1.47E+01	−2.06E+01	−1.38E+01	−2.58E+01	−1.66E+01	−2.30E+01	−1.75E+01	−2.25E+01	−2.99E+00	−2.66E+01	−6.41E+00	−9.51E+00
	Worst	−2.30E+01	−1.39E+01	−1.73E+01	−1.22E+01	−2.14E+01	−4.56E+00	−1.31E+01	−2.51E+00	−1.81E+01	−2.51E+00	−1.80E+01	−2.51E+00	−2.51E+00
	Std	2.32E+00	3.90E-01	1.55E+00	8.01E-01	2.23E+00	6.21E+00	4.93E+00	8.52E+00	2.22E+00	2.54E-01	3.90E+00	2.05E+00	3.60E+00
	Median	−2.30E+01	−1.40E+01	−1.93E+01	−1.34E+01	−2.17E+01	−5.00E+00	−2.20E+01	−9.47E+00	−1.91E+01	−2.51E+00	−2.16E+01	−2.51E+00	−2.75E+00
	Rank	1	7	6	8	2	10	4	9	5	13	3	12	11
C11-F7	Mean	8.61E-01	1.59E+00	1.28E+00	1.90E+00	9.28E-01	1.29E+00	1.73E+00	8.81E-01	1.06E+00	1.70E+00	1.08E+00	1.12E+00	1.72E+00
	Best	5.82E-01	1.53E+00	1.14E+00	1.66E+00	7.54E-01	1.12E+00	1.61E+00	8.21E-01	8.19E-01	1.52E+00	8.78E-01	8.35E-01	1.33E+00
	Worst	1.03E+00	1.70E+00	1.42E+00	2.08E+00	1.01E+00	1.65E+00	1.90E+00	9.53E-01	1.28E+00	1.84E+00	1.27E+00	1.36E+00	1.93E+00
	Std	2.12E-01	8.30E-02	1.62E-01	1.83E-01	1.25E-01	2.56E-01	1.29E-01	6.69E-02	2.01E-01	1.50E-01	1.86E-01	2.80E-01	2.82E-01
	Median	9.18E-01	1.57E+00	1.27E+00	1.92E+00	9.74E-01	1.20E+00	1.70E+00	8.74E-01	1.08E+00	1.72E+00	1.08E+00	1.14E+00	1.82E+00
	Rank	1	9	7	13	3	8	12	2	4	10	5	6	11
C11-F8	Mean	2.20E+02	2.84E+02	2.40E+02	3.24E+02	2.22E+02	2.57E+02	2.65E+02	2.24E+02	2.27E+02	2.24E+02	2.46E+02	4.65E+02	2.22E+02
	Best	2.20E+02	2.58E+02	2.24E+02	2.83E+02	2.20E+02	2.20E+02	2.45E+02	2.20E+02	2.20E+02	2.20E+02	2.20E+02	2.48E+02	2.20E+02
	Worst	2.20E+02	3.18E+02	2.57E+02	3.68E+02	2.25E+02	3.52E+02	3.11E+02	2.36E+02	2.34E+02	2.36E+02	2.92E+02	5.64E+02	2.30E+02
	Std	0.00E+00	2.77E+01	1.50E+01	3.63E+01	2.92E+00	6.74E+01	3.20E+01	8.43E+00	8.76E+00	8.43E+00	3.60E+01	1.58E+02	5.15E+00
	Median	2.20E+02	2.80E+02	2.40E+02	3.22E+02	2.22E+02	2.27E+02	2.53E+02	2.20E+02	2.27E+02	2.20E+02	2.36E+02	5.25E+02	2.20E+02
	Rank	1	10	6	11	2	8	9	4	5	4	7	12	3
C11-F9	Mean	8.79E+03	5.49E+05	3.73E+05	1.05E+06	2.00E+04	6.53E+04	3.69E+05	1.31E+05	4.25E+04	4.02E+05	8.11E+05	1.07E+06	1.91E+06
	Best	5.46E+03	3.67E+05	3.29E+05	6.83E+05	1.10E+04	4.69E+04	2.04E+05	7.45E+04	1.83E+04	3.33E+05	6.94E+05	8.55E+05	1.83E+06
	Worst	1.40E+04	6.30E+05	4.01E+05	1.23E+06	2.83E+04	8.29E+04	6.25E+05	1.99E+05	7.41E+04	5.16E+05	8.73E+05	1.31E+06	2.02E+06
	Std	3.89E+03	1.31E+05	3.30E+04	2.59E+05	8.11E+03	1.61E+04	2.02E+05	5.40E+04	2.48E+04	8.49E+04	8.36E+04	2.53E+05	9.91E+04
	Median	7.83E+03	5.99E+05	3.80E+05	1.14E+06	2.04E+04	6.58E+04	3.23E+05	1.26E+05	3.88E+04	3.80E+05	8.38E+05	1.05E+06	1.90E+06
	Rank	1	9	7	11	2	4	6	5	3	8	10	12	13
C11-F10	Mean	−2.15E+01	−1.41E+01	−1.70E+01	−1.24E+01	−1.91E+01	−1.45E+01	−1.30E+01	−1.48E+01	−1.42E+01	−1.15E+01	−1.33E+01	−1.16E+01	−1.13E+01
	Best	−2.18E+01	−1.53E+01	−1.72E+01	−1.28E+01	−1.95E+01	−1.89E+01	−1.37E+01	−2.12E+01	−1.47E+01	−1.16E+01	−1.38E+01	−1.16E+01	−1.13E+01
	Worst	−2.08E+01	−1.35E+01	−1.66E+01	−1.22E+01	−1.87E+01	−1.22E+01	−1.25E+01	−1.16E+01	−1.31E+01	−1.14E+01	−1.25E+01	−1.15E+01	−1.12E+01
	Std	4.99E-01	8.54E-01	2.81E-01	2.99E-01	4.20E-01	3.18E+00	4.91E-01	4.54E+00	8.09E-01	9.21E-02	6.57E-01	4.73E-02	6.46E-02
	Median	−2.17E+01	−1.38E+01	−1.71E+01	−1.24E+01	−1.91E+01	−1.35E+01	−1.29E+01	−1.32E+01	−1.46E+01	−1.15E+01	−1.34E+01	−1.16E+01	−1.13E+01
	Rank	1	7	3	10	2	5	9	4	6	12	8	11	13
C11-F11	Mean	5.72E+05	5.71E+06	9.84E+05	8.72E+06	1.64E+06	5.85E+06	1.20E+06	1.30E+06	3.78E+06	5.13E+06	1.40E+06	5.14E+06	6.03E+06
	Best	2.61E+05	5.45E+06	7.64E+05	8.42E+06	1.53E+06	4.87E+06	1.09E+06	6.14E+05	3.59E+06	5.10E+06	1.25E+06	5.12E+06	5.98E+06
	Worst	8.29E+05	6.08E+06	1.17E+06	8.91E+06	1.78E+06	7.08E+06	1.37E+06	2.69E+06	4.12E+06	5.16E+06	1.57E+06	5.17E+06	6.10E+06
	Std	2.61E+05	3.09E+05	1.84E+05	2.19E+05	1.28E+05	9.57E+05	1.23E+05	9.93E+05	2.50E+05	2.85E+04	1.39E+05	2.58E+04	5.31E+04
	Median	5.99E+05	5.67E+06	1.00E+06	8.77E+06	1.63E+06	5.73E+06	1.18E+06	9.37E+05	3.70E+06	5.13E+06	1.38E+06	5.14E+06	6.02E+06
	Rank	1	10	2	13	6	11	3	4	7	8	5	9	12
C11-F12	Mean	1.20E+06	8.27E+06	3.34E+06	1.30E+07	1.27E+06	4.96E+06	5.74E+06	1.33E+06	1.42E+06	1.41E+07	5.71E+06	2.29E+06	1.43E+07
	Best	1.16E+06	7.93E+06	3.24E+06	1.21E+07	1.20E+06	4.70E+06	5.33E+06	1.18E+06	1.26E+06	1.33E+07	5.43E+06	2.13E+06	1.41E+07
	Worst	1.25E+06	8.57E+06	3.41E+06	1.38E+07	1.35E+06	5.10E+06	5.94E+06	1.47E+06	1.56E+06	1.47E+07	5.92E+06	2.50E+06	1.44E+07
	Std	4.72E+04	2.81E+05	7.77E+04	7.51E+05	7.08E+04	1.97E+05	2.98E+05	1.24E+05	1.30E+05	6.47E+05	2.21E+05	1.61E+05	1.10E+05
	Median	1.20E+06	8.29E+06	3.36E+06	1.31E+07	1.27E+06	5.03E+06	5.84E+06	1.33E+06	1.43E+06	1.42E+07	5.75E+06	2.28E+06	1.43E+07
	Rank	1	10	6	11	2	7	9	3	4	12	8	5	13
C11-F13	Mean	1.54E+04	1.59E+04	1.54E+04	1.63E+04	1.55E+04	1.55E+04	1.55E+04	1.55E+04	1.55E+04	1.59E+04	1.26E+05	1.55E+04	2.98E+04
	Best	1.54E+04	1.57E+04	1.54E+04	1.59E+04	1.55E+04	1.55E+04	1.55E+04	1.55E+04	1.55E+04	1.56E+04	9.15E+04	1.55E+04	1.55E+04
	Worst	1.54E+04	1.63E+04	1.54E+04	1.73E+04	1.55E+04	1.55E+04	1.56E+04	1.55E+04	1.55E+04	1.65E+04	1.74E+05	1.55E+04	7.23E+04
	Std	9.09E-03	3.13E+02	9.16E-01	7.18E+02	2.89E+00	1.15E+01	4.93E+01	2.82E+01	8.66E+00	4.07E+02	3.90E+04	2.54E+01	2.98E+04
	Median	1.54E+04	1.57E+04	1.54E+04	1.60E+04	1.55E+04	1.55E+04	1.55E+04	1.55E+04	1.55E+04	1.58E+04	1.20E+05	1.55E+04	1.56E+04
	Rank	1	9	2	11	3	4	8	7	6	10	13	5	12
C11-F14	Mean	1.83E+04	1.11E+05	1.85E+04	2.26E+05	1.86E+04	1.95E+04	1.92E+04	1.94E+04	1.92E+04	3.06E+05	1.91E+04	1.91E+04	1.91E+04
	Best	1.82E+04	8.45E+04	1.84E+04	1.66E+05	1.85E+04	1.93E+04	1.91E+04	1.93E+04	1.91E+04	3.01E+04	1.88E+04	1.90E+04	1.88E+04
	Worst	1.84E+04	1.55E+05	1.86E+04	3.25E+05	1.87E+04	2.00E+04	1.93E+04	1.95E+04	1.94E+04	5.90E+05	1.93E+04	1.93E+04	1.94E+04
	Std	7.16E+01	3.32E+04	1.05E+02	7.48E+04	7.22E+01	3.81E+02	1.31E+02	7.99E+01	1.51E+02	2.83E+05	2.23E+02	1.32E+02	2.46E+02
	Median	1.83E+04	1.02E+05	1.85E+04	2.06E+05	1.86E+04	1.94E+04	1.92E+04	1.94E+04	1.92E+04	3.02E+05	1.91E+04	1.91E+04	1.91E+04
	Rank	1	11	2	12	3	10	7	9	8	13	4	6	5
C11-F15	Mean	3.29E+04	8.94E+05	1.07E+05	1.89E+06	3.29E+04	5.42E+04	2.16E+05	3.31E+04	3.31E+04	1.52E+07	2.96E+05	3.33E+04	7.81E+06
	Best	3.28E+04	3.69E+05	4.30E+04	7.89E+05	3.29E+04	3.30E+04	3.30E+04	3.30E+04	3.30E+04	3.18E+06	2.62E+05	3.33E+04	3.56E+06
	Worst	3.30E+04	2.25E+06	1.78E+05	4.92E+06	3.30E+04	1.17E+05	3.09E+05	3.32E+04	3.31E+04	2.26E+07	3.19E+05	3.33E+04	1.34E+07
	Std	7.69E+01	9.52E+05	7.62E+04	2.13E+06	6.43E+01	4.43E+04	1.31E+05	6.62E+01	4.92E+01	9.30E+06	2.79E+04	9.30E+00	4.74E+06
	Median	3.29E+04	4.80E+05	1.03E+05	9.16E+05	3.30E+04	3.32E+04	2.61E+05	3.31E+04	3.31E+04	1.75E+07	3.01E+05	3.33E+04	7.15E+06
	Rank	1	10	7	11	2	6	8	4	3	13	9	5	12
C11-F16	Mean	1.34E+05	9.33E+05	1.35E+05	1.92E+06	1.38E+05	1.45E+05	1.42E+05	1.42E+05	1.46E+05	8.75E+07	1.84E+07	7.83E+07	7.52E+07
	Best	1.31E+05	2.87E+05	1.34E+05	4.68E+05	1.36E+05	1.42E+05	1.36E+05	1.33E+05	1.43E+05	8.53E+07	9.36E+06	6.48E+07	6.08E+07
	Worst	1.36E+05	2.20E+06	1.36E+05	4.77E+06	1.41E+05	1.47E+05	1.47E+05	1.50E+05	1.51E+05	9.00E+07	3.33E+07	9.36E+07	9.62E+07
	Std	2.39E+03	9.05E+05	1.08E+03	2.03E+06	2.70E+03	2.37E+03	4.83E+03	7.52E+03	3.89E+03	2.09E+06	1.09E+07	1.31E+07	1.58E+07
	Median	1.33E+05	6.22E+05	1.36E+05	1.22E+06	1.37E+05	1.45E+05	1.42E+05	1.42E+05	1.44E+05	8.74E+07	1.55E+07	7.75E+07	7.19E+07
	Rank	1	8	2	9	3	6	5	4	7	13	10	12	11
C11-F17	Mean	1.93E+06	8.82E+09	2.28E+09	1.53E+10	2.29E+06	1.26E+09	9.54E+09	3.09E+06	3.00E+06	2.20E+10	1.10E+10	2.05E+10	2.15E+10
	Best	1.92E+06	7.52E+09	2.07E+09	1.10E+10	1.96E+06	1.04E+09	6.81E+09	2.29E+06	2.04E+06	2.11E+10	9.71E+09	1.81E+10	2.01E+10
	Worst	1.94E+06	9.78E+09	2.49E+09	1.87E+10	2.89E+06	1.44E+09	1.27E+10	3.71E+06	4.83E+06	2.29E+10	1.17E+10	2.37E+10	2.43E+10
	Std	1.20E+04	1.05E+09	1.96E+08	3.47E+09	4.41E+05	2.17E+08	2.60E+09	6.90E+05	1.33E+06	7.79E+08	9.46E+08	2.66E+09	2.00E+09
	Median	1.92E+06	9.00E+09	2.28E+09	1.57E+10	2.15E+06	1.28E+09	9.34E+09	3.18E+06	2.57E+06	2.19E+10	1.14E+10	2.01E+10	2.08E+10
	Rank	1	7	6	10	2	5	8	4	3	13	9	11	12
C11-F18	Mean	9.42E+05	5.41E+07	6.47E+06	1.17E+08	9.71E+05	2.03E+06	9.45E+06	9.87E+05	1.03E+06	3.05E+07	1.10E+07	1.33E+08	1.13E+08
	Best	9.38E+05	3.72E+07	3.90E+06	8.05E+07	9.50E+05	1.78E+06	4.07E+06	9.64E+05	9.67E+05	2.42E+07	8.19E+06	1.12E+08	1.09E+08
	Worst	9.45E+05	6.16E+07	1.11E+07	1.33E+08	1.03E+06	2.37E+06	1.66E+07	9.98E+05	1.20E+06	3.30E+07	1.38E+07	1.47E+08	1.17E+08
	Std	2.77E+03	1.20E+07	3.52E+06	2.59E+07	4.03E+04	2.99E+05	5.55E+06	1.69E+04	1.17E+05	4.45E+06	2.65E+06	1.69E+07	3.56E+06
	Median	9.43E+05	5.89E+07	5.45E+06	1.26E+08	9.53E+05	1.99E+06	8.57E+06	9.94E+05	9.78E+05	3.25E+07	1.09E+07	1.36E+08	1.13E+08
	Rank	1	10	6	12	2	5	7	3	4	9	8	13	11
C11-F19	Mean	1.03E+06	5.33E+07	6.57E+06	1.14E+08	1.14E+06	2.44E+06	1.01E+07	1.47E+06	1.36E+06	3.51E+07	6.19E+06	1.70E+08	1.13E+08
	Best	9.68E+05	4.55E+07	6.00E+06	9.86E+07	1.07E+06	2.20E+06	2.04E+06	1.13E+06	1.23E+06	2.46E+07	2.37E+06	1.54E+08	1.10E+08
	Worst	1.17E+06	6.78E+07	7.95E+06	1.44E+08	1.29E+06	2.88E+06	1.82E+07	1.94E+06	1.54E+06	4.37E+07	8.12E+06	1.97E+08	1.16E+08
	Std	9.97E+04	1.06E+07	9.77E+05	2.20E+07	1.09E+05	3.14E+05	8.01E+06	3.60E+05	1.37E+05	8.73E+06	2.74E+06	1.93E+07	2.67E+06
	Median	9.83E+05	5.00E+07	6.16E+06	1.07E+08	1.09E+06	2.34E+06	1.00E+07	1.40E+06	1.33E+06	3.60E+07	7.13E+06	1.65E+08	1.13E+08
	Rank	1	10	7	12	2	5	8	4	3	9	6	13	11
C11-F20	Mean	9.41E+05	5.67E+07	5.82E+06	1.23E+08	9.60E+05	1.81E+06	7.18E+06	9.72E+05	9.98E+05	3.41E+07	1.41E+07	1.57E+08	1.13E+08
	Best	9.36E+05	4.99E+07	5.13E+06	1.08E+08	9.57E+05	1.63E+06	6.77E+06	9.63E+05	9.77E+05	3.33E+07	9.35E+06	1.43E+08	1.08E+08
	Worst	9.47E+05	6.71E+07	6.55E+06	1.47E+08	9.62E+05	2.11E+06	7.74E+06	9.83E+05	1.01E+06	3.49E+07	2.18E+07	1.70E+08	1.18E+08
	Std	5.01E+03	7.72E+06	6.20E+05	1.73E+07	2.30E+03	2.41E+05	4.35E+05	9.61E+03	1.66E+04	6.79E+05	5.70E+06	1.58E+07	4.28E+06
	Median	9.41E+05	5.49E+07	5.79E+06	1.19E+08	9.61E+05	1.75E+06	7.11E+06	9.72E+05	1.00E+06	3.40E+07	1.26E+07	1.57E+08	1.14E+08
	Rank	1	10	6	12	2	5	7	3	4	9	8	13	11
C11-F21	Mean	1.27E+01	4.97E+01	2.15E+01	7.55E+01	1.59E+01	2.96E+01	3.84E+01	2.73E+01	2.22E+01	9.94E+01	4.03E+01	1.04E+02	1.01E+02
	Best	9.97E+00	4.10E+01	2.01E+01	5.63E+01	1.37E+01	2.62E+01	3.52E+01	2.43E+01	2.04E+01	4.78E+01	3.55E+01	9.02E+01	5.81E+01
	Worst	1.50E+01	5.90E+01	2.33E+01	9.47E+01	1.82E+01	3.11E+01	4.24E+01	3.03E+01	2.46E+01	1.46E+02	4.32E+01	1.16E+02	1.24E+02
	Std	2.41E+00	8.18E+00	1.44E+00	1.78E+01	2.18E+00	2.37E+00	3.35E+00	3.58E+00	1.93E+00	4.24E+01	3.60E+00	1.34E+01	3.20E+01
	Median	1.30E+01	4.94E+01	2.13E+01	7.55E+01	1.58E+01	3.05E+01	3.80E+01	2.73E+01	2.20E+01	1.02E+02	4.12E+01	1.06E+02	1.12E+02
	Rank	1	9	3	10	2	6	7	5	4	11	8	13	12
C11-F22	Mean	1.61E+01	4.64E+01	2.73E+01	6.28E+01	1.90E+01	3.19E+01	4.59E+01	3.21E+01	2.49E+01	1.01E+02	4.62E+01	1.05E+02	9.14E+01
	Best	1.15E+01	4.03E+01	2.20E+01	4.56E+01	1.61E+01	2.78E+01	3.96E+01	2.46E+01	2.39E+01	6.56E+01	3.87E+01	8.82E+01	9.04E+01
	Worst	1.96E+01	5.19E+01	3.25E+01	7.23E+01	2.12E+01	3.45E+01	5.06E+01	3.71E+01	2.56E+01	1.20E+02	5.50E+01	1.16E+02	9.31E+01
	Std	4.20E+00	5.20E+00	5.23E+00	1.24E+01	2.57E+00	3.00E+00	5.24E+00	5.84E+00	8.40E-01	2.57E+01	7.05E+00	1.33E+01	1.20E+00
	Median	1.67E+01	4.66E+01	2.73E+01	6.66E+01	1.94E+01	3.26E+01	4.67E+01	3.32E+01	2.50E+01	1.10E+02	4.56E+01	1.08E+02	9.11E+01
	Rank	1	9	4	10	2	5	7	6	3	12	8	13	11
Sum rank		22	191	109	231	55	146	145	118	97	222	157	198	224
Mean rank		1.00E+00	8.68E+00	4.95E+00	1.05E+01	2.50E+00	6.64E+00	6.59E+00	5.36E+00	4.41E+00	1.01E+01	7.14E+00	9.00E+00	1.02E+01
Total rank		1	2	12	4	13	3	11	9	6	7	10	5	8
Wilcoxon: *p*-value			3.85E-12	6.82E-15	1.37E-15	1.54E-03	4.30E-15	4.62E-15	1.41E-11	1.69E-12	2.94E-15	7.07E-15	1.37E-15	2.01E-15

**Table 8:** Performance of optimization algorithms on pressure vessel design problem.

Algorithm	Optimum Variables				Optimum Cost
	*T_s_*	*T_h_*	*R*	*L*	
LOA	0.7780271	0.3845792	40.312284	200	5882.9013
WSO	0.7780271	0.3845792	40.312284	200	5882.9013
AVOA	0.7780312	0.3845812	40.312495	199.99706	5882.9083
RSA	1.235533	0.6652352	62.515681	33.265801	7942.1971
MPA	0.7780271	0.3845792	40.312284	200	5882.9013
TSA	0.7796425	0.3859395	40.39373	200	5911.9472
WOA	0.9244877	0.4575332	46.805861	127.41082	6308.5588
MVO	0.8399069	0.4194976	43.500091	160.39975	6015.6018
GWO	0.778502	0.3859326	40.321438	199.96097	5890.9207
TLBO	1.6384603	0.4907006	48.413435	117.32657	11285.858
GSA	1.1642139	1.2327499	44.479077	189.89246	12577.279
PSO	1.6250756	0.6463035	65.357519	35.189096	10398.551
GA	1.4674754	0.8220162	59.996638	61.718062	11409.75

**Table 9:** Statistical results of optimization algorithms on pressure vessel design problem.

Algorithm	Mean	Best	Worst	Std	Median	Rank
LOA	5884.8955	5882.9013	5885.8955	1.87E-12	5883.8955	1
WSO	5892.0349	5882.9013	5973.0173	24.377865	5882.9017	3
AVOA	6252.2485	5882.9083	7159.3658	386.59588	6063.7079	5
RSA	13043.76	7942.1971	21362.677	3430.9106	11939.739	9
MPA	5882.9013	5882.9013	5882.9013	4.04E-06	5882.9013	2
TSA	6308.8549	5911.9472	7051.9078	365.53019	6168.9479	6
WOA	8204.2156	6308.5588	13478.021	1845.3213	7744.4986	8
MVO	6579.8221	6015.6018	7163.8481	351.44321	6639.1657	7
GWO	6024.9473	5890.9207	6747.5707	262.64571	5900.0698	4
TLBO	30448.932	11285.858	65600.284	15136.124	26830.649	12
GSA	22077.832	12577.279	34653.395	7363.816	21185.622	10
PSO	32000.589	10398.551	55068.219	14170.253	35315.967	13
GA	27326.87	11409.75	49383.377	11881.012	24170.859	11

**Table 10:** Performance of optimization algorithms on speed reducer design problem.

Algorithm	Optimum Variables							Optimum Cost
	b	*M*	*p*	*l*_1_	*l*_2_	*d*_1_	*d*_2_	
LOA	3.5	0.7	17	7.3	7.8	3.3502147	5.2866832	2996.3482
WSO	3.5000004	0.7	17	7.3000096	7.8000004	3.3502148	5.2866833	2996.3483
AVOA	3.5	0.7	17	7.3000007	7.8	3.3502147	5.2866832	2996.3482
RSA	3.5890909	0.7	17	8.1909092	8.2454546	3.3554815	5.4767291	3176.6022
MPA	3.5	0.7	17	7.3	7.8	3.3502147	5.2866832	2996.3482
TSA	3.5124675	0.7	17	7.3	8.2454546	3.3505297	5.2900982	3013.2903
WOA	3.5845497	0.7	17	7.3	8.0023373	3.3612308	5.2867533	3036.8503
MVO	3.5021766	0.7	17	7.3	8.0600548	3.3689471	5.2868752	3007.8372
GWO	3.5006198	0.7	17	7.3049714	7.8	3.3634887	5.2887389	3001.3413
TLBO	3.5542231	0.7038638	26.012217	8.0746042	8.1336704	3.6529699	5.3375981	5194.3129
GSA	3.5221446	0.7026613	17.356813	7.8031437	7.886617	3.4068193	5.3826203	3163.9329
PSO	3.5079104	0.7000696	18.05906	7.3957302	7.8657581	3.58726	5.3421086	3292.3111
GA	3.5754084	0.7053795	17.786641	7.7277995	7.8539789	3.6898264	5.3443414	3.34E+03

**Table 11:** Statistical results of optimization algorithms on speed reducer design problem.

Algorithm	Mean	Best	Worst	Std	Median	Rank
LOA	2996.3482	2996.3482	2996.3482	9.33E-13	2996.3482	1
WSO	2996.6222	2996.3483	2998.7174	0.5653183	2996.3638	3
AVOA	3000.7054	2996.3482	3010.5833	3.8354885	3000.6092	4
RSA	3267.418	3176.6022	3323.7798	55.595014	3281.7991	9
MPA	2996.3482	2996.3482	2996.3482	3.08E-06	2996.3482	2
TSA	3030.9376	3013.2903	3044.21	9.8011089	3032.6658	7
WOA	3144.9205	3036.8503	3430.127	102.74833	3112.6957	8
MVO	3028.7049	3007.8372	3067.7126	12.814709	3029.1301	6
GWO	3004.3452	3001.3413	3010.1109	2.4235536	3003.8446	5
TLBO	6.723E+13	5194.3129	4.866E+14	1.119E+14	2.633E+13	12
GSA	3439.3435	3163.9329	4039.6332	253.44874	3314.0241	10
PSO	9.922E+13	3292.3111	5.026E+14	1.198E+14	7.097E+13	13
GA	4.777E+13	3335.1496	3.084E+14	7.525E+13	1.914E+13	11

**Table 12:** Performance of optimization algorithms on welded beam design problem.

Algorithm	Optimum Variables				Optimum Cost
	*h*	*l*	*t*	*b*	
LOA	0.2057296	3.4704887	9.0366239	0.2057296	1.7246798
WSO	0.2057299	3.4704894	9.0366202	0.2057319	1.7248523
AVOA	0.2049905	3.486517	9.0365208	0.2057343	1.7258836
RSA	0.1969993	3.5325121	9.8949045	0.2173906	1.9669902
MPA	0.2057296	3.4704887	9.0366239	0.2057296	1.7248523
TSA	0.2042474	3.4945379	9.0632589	0.206142	1.7335408
WOA	0.2134582	3.3344888	8.9759393	0.2204819	1.8180608
MVO	0.2059842	3.4650021	9.0444134	0.2060446	1.728246
GWO	0.2055967	3.4735386	9.0362531	0.2057964	1.7255009
TLBO	0.3115492	4.3894035	6.8734148	0.4176705	2.9796461
GSA	0.2908557	2.7470518	7.4758714	0.3044843	2.0722963
PSO	0.3668896	3.4262313	7.4019025	0.5614787	3.9449745
GA	0.2236798	6.7978876	7.806539	0.3010265	2.7258444

**Table 13:** Statistical results of optimization algorithms on welded beam design problem.

Algorithm	Mean	Best	Worst	Std	Median	Rank
LOA	1.7246798	1.7246798	1.7246798	2.28E-16	1.7246798	1
WSO	1.7248526	1.7248523	1.7248577	1.208E-06	1.7248523	3
AVOA	1.75998	1.7258836	1.8386827	0.0352307	1.7465598	7
RSA	2.166118	1.9669902	2.5019493	0.1392497	2.1419303	8
MPA	1.7248523	1.7248523	1.7248523	3.24E-09	1.7248523	2
TSA	1.7425281	1.7335408	1.7514086	0.0054155	1.7426211	6
WOA	2.290829	1.8180608	3.9673497	0.6199333	2.0735215	9
MVO	1.740667	1.728246	1.7733447	0.0132901	1.736735	5
GWO	1.7271711	1.7255009	1.7310777	0.0013165	1.7269342	4
TLBO	3.213E+13	2.9796461	3.101E+14	7.837E+13	5.556825	12
GSA	2.4193009	2.0722963	2.7174014	0.1850149	2.4478414	10
PSO	4.431E+13	3.9449745	2.682E+14	8.463E+13	6.5600701	13
GA	1.088E+13	2.7258444	1.177E+14	3.339E+13	5.5245557	11

**Table 14:** Performance of optimization algorithms on tension/compression spring design problem.

Algorithm	Optimum Variables			Optimum Cost
	*d*	*D*	*P*	
LOA	0.0516891	0.3567177	11.288966	0.0126019
WSO	0.0516871	0.3566717	11.291665	0.0126652
AVOA	0.0512086	0.345282	11.996535	0.01267
RSA	0.0501843	0.3156108	14.59516	0.0131412
MPA	0.0516907	0.3567569	11.28667	0.0126652
TSA	0.0510126	0.3406634	12.31184	0.0126814
WOA	0.051184	0.3447029	12.034448	0.0126705
MVO	0.0501843	0.3212096	13.796911	0.0127469
GWO	0.0519471	0.3629418	10.937856	0.0126705
TLBO	0.0671857	0.8735239	3.0133407	0.0173143
GSA	0.0549942	0.4382505	7.9387706	0.0130596
PSO	0.0671061	0.8705176	3.0133407	0.0172159
GA	0.067637	0.881056	3.0133407	0.0176948

**Table 15:** Statistical results of optimization algorithms on tension/compression spring design problem.

Algorithm	Mean	Best	Worst	Std	Median	Rank
LOA	0.0126019	0.0126019	0.0126019	6.88E-18	0.0126019	1
WSO	0.0126759	0.0126652	0.0128186	3.417E-05	0.0126656	3
AVOA	0.0133112	0.01267	0.0140833	0.0005314	0.0132462	8
RSA	0.0132192	0.0131412	0.0133564	6.614E-05	0.0131991	6
MPA	0.0126652	0.0126652	0.0126652	2.72E-09	0.0126652	2
TSA	0.0129486	0.0126814	0.013486	0.0002303	0.0128784	5
WOA	0.013244	0.0126705	0.0144134	0.000576	0.0130551	7
MVO	0.0162965	0.0127469	0.0176669	0.0015702	0.0171696	9
GWO	0.0127203	0.0126705	0.0129331	5.272E-05	0.0127179	4
TLBO	0.017821	0.0173143	0.0183997	0.0003413	0.0177789	10
GSA	0.019108	0.0130596	0.0311597	0.0040605	0.0187018	11
PSO	1.994E+13	0.0172159	3.539E+14	7.918E+13	0.0172159	13
GA	1.558E+12	0.0176948	1.611E+13	4.652E+12	0.0249529	12

## Data Availability

Not applicable.
